# Bacillimidazoles A−F, Imidazolium-Containing Compounds Isolated from a Marine *Bacillus*

**DOI:** 10.3390/md20010043

**Published:** 2022-01-01

**Authors:** Jia-Xuan Yan, Qihao Wu, Eric J. N. Helfrich, Marc G. Chevrette, Doug R. Braun, Heino Heyman, Gene E. Ananiev, Scott R. Rajski, Cameron R. Currie, Jon Clardy, Tim S. Bugni

**Affiliations:** 1 Pharmaceutical Sciences Division, University of Wisconsin-Madison, 777 Highland Ave, Madison, WI 53705, USA; jiaxuan.yan@merck.com (J.-X.Y.); qihao.wu@yale.edu (Q.W.); drbraun1@wisc.edu (D.R.B.); scott.rajski@wisc.edu (S.R.R.); 2 Harvard Medical School, Harvard University, 240 Longwood Ave, Boston, MA 02115, USA; eric.helfrich@bio.uni-frankfurt.de (E.J.N.H.); jon_clardy@hms.harvard.edu (J.C.); 3 Department of Bacteriology, University of Wisconsin-Madison, 1550 Linden Ave, Madison, WI 53706, USA; chevrette@wisc.edu (M.G.C.); currie@bact.wisc.edu (C.R.C.); 4 Bruker Daltonics, Bruker Scientific LLC., 40 Manning Rd, Billerica, MA 01821, USA; heino@metabolon.com; 5 The Small Molecule Screening Facility (SMSF), University of Wisconsin-Madison, 600 Highland Ave, Madison, WI 53792, USA; geananiev@wisc.edu

**Keywords:** marine-derived *Bacillus*, antibacterial, biosynthetic gene cluster, isotopic enrichment, heterocycles, imidazolium

## Abstract

Chemical investigations of a marine sponge-associated *Bacillus* revealed six new imidazolium-containing compounds, bacillimidazoles A–F (**1**–**6**). Previous reports of related imidazolium-containing natural products are rare. Initially unveiled by timsTOF (trapped ion mobility spectrometry) MS data, extensive HRMS and 1D and 2D NMR analyses enabled the structural elucidation of **1**–**6**. In addition, a plausible biosynthetic pathway to bacillimidazoles is proposed based on isotopic labeling experiments and invokes the highly reactive glycolytic adduct 2,3-butanedione. Combined, the results of structure elucidation efforts, isotopic labeling studies and bioinformatics suggest that **1**–**6** result from a fascinating intersection of primary and secondary metabolic pathways in *Bacillus* sp. WMMC1349. Antimicrobial assays revealed that, of **1**–**6**, only compound **six** displayed discernible antibacterial activity, despite the close structural similarities shared by all six natural products.

## 1. Introduction

Heterocyclic scaffolds are commonly encountered in natural products isolated from both terrestrial and marine organisms [1,2,3,4]. Their vast structural diversity, drug-like features, and biological properties have inspired both intensive efforts to discover new heterocyclic compounds, as well as imaginative total syntheses [4]. Nitrogen-containing heterocycles, such as pyrroles, imidazoles, oxazoles, pyridines, and quinolones, exhibit a diverse array of biological activities; these include, but are by no means limited to, antibacterial [5], antifungal [6], and anticancer [7] activities.

Marine-derived imidazole alkaloids have been one of the most fruitful families of bioactive compounds giving rise to many pharmaceutical leads [8]. Historically, marine-derived imidazole alkaloids have been most often isolated from sponges; imidazole alkaloids [9] featuring bromopyrrole-imidazoles [10], indole-containing imidazoles [11], and 2-aminoimidazoles are among the species most often identified from marine sponges [12]. Furthermore, in recent years, marine microorganisms have come to be viewed as sustainable and productive sources of new bioactive imidazole-containing natural products [9]. However, reports of positively charged imidazolium natural products remain relatively rare. Most reports of imidazolium-containing compounds feature 1,3-dimethyl-5-methylthiol [13,14,15] or 2-aminoimidazolium containing structures (guanidinium-like) [10,11]. A wide array of applications in ionic liquids [16], important biological activities [17], and their amenability to further structural modifications have made imidazolium salts an attractive target of contemporary research [18]. Therefore, discovering new imidazolium-based species and gaining further insight into their biosynthetic origins has become an interesting, yet challenging, task for natural product scientists.

## 2. Results & Discussion

**Figure 1:**
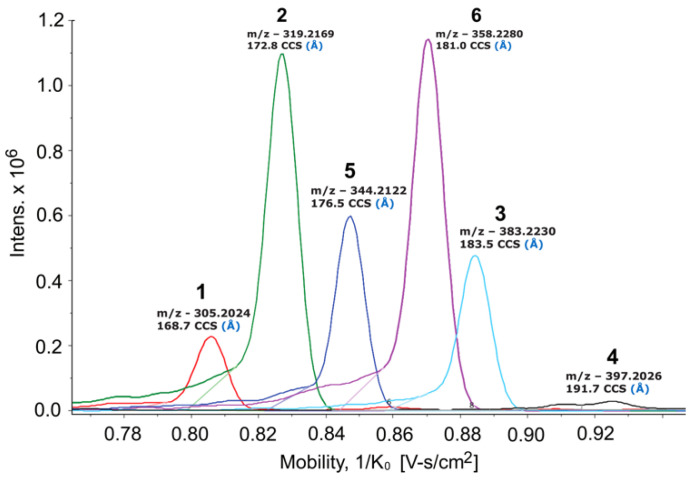
Bruker timsTOF MS spectrum of the bioactive wells against MRSA. Peak/compound assignments are shown above each relevant signal.

**Figure 2:**
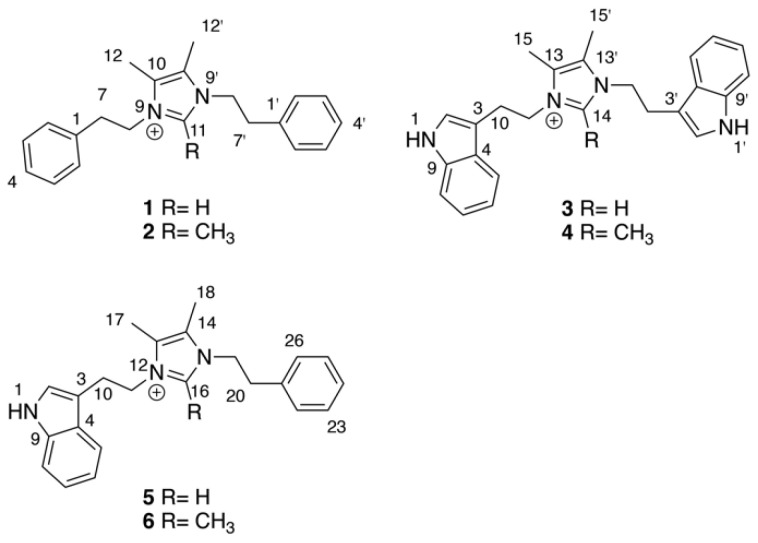
Structures of bacillimidazoles A–F (**1**–**6**) with central imidazolium numberings indicated for each subgroup.

As part of our ongoing efforts to discover new natural products from marine invertebrate-associated bacteria [19], we developed a streamlined discovery platform that includes strain prioritization by metabolomics [20] and an LC/MS fractionation platform to generate screening libraries [21]. Strain WMMC1349, a marine *Bacillus* sp. cultivated from the sponge *Cinachyrella apion*, drew our attention since one of its fractions displayed activity against methicillin-resistant *Staphylococcus aureus* (MRSA). Further purification of the active fraction by HPLC resulted in enrichment of an active subfraction. Interestingly, subsequent analytical HPLC revealed this fraction to be a represented by a broad peak (Figure S1) despite the clear presence of a mixture of compounds as revealed by ^1^H NMR (Figure S1). Fortunately, Bruker timsTOF (trapped ion mobility spectrometry) MS data for this subfraction indicated a series of new molecules with *m*/*z* values of 305.2, 319.2, 344.4, 358.2, 383.2, and 397.2, respectively (Figure 1). Exhaustive attempts to separate each different *m*/*z* species (see Figures S1–S55), as initially visualized by timsTOF MS, ultimately afforded HPLC conditions amenable to clean separation and isolation of each discreet compound. The six new imidazolium-containing compounds, now differentiated from each other, were termed bacillimidazoles A–F (**1**–**6**, Figure 2), and all were assessed for in vitro activity against MRSA, *B. subtilis,* and *E. coli*; only compound **6** was found to be active (MRSA). Isotopic labelling of these metabolites using isotopically enriched culture media, and bioinformatic analysis were conducted to decipher the means by which the bacillimidazoles are biosynthesized.

**Figure 3:**
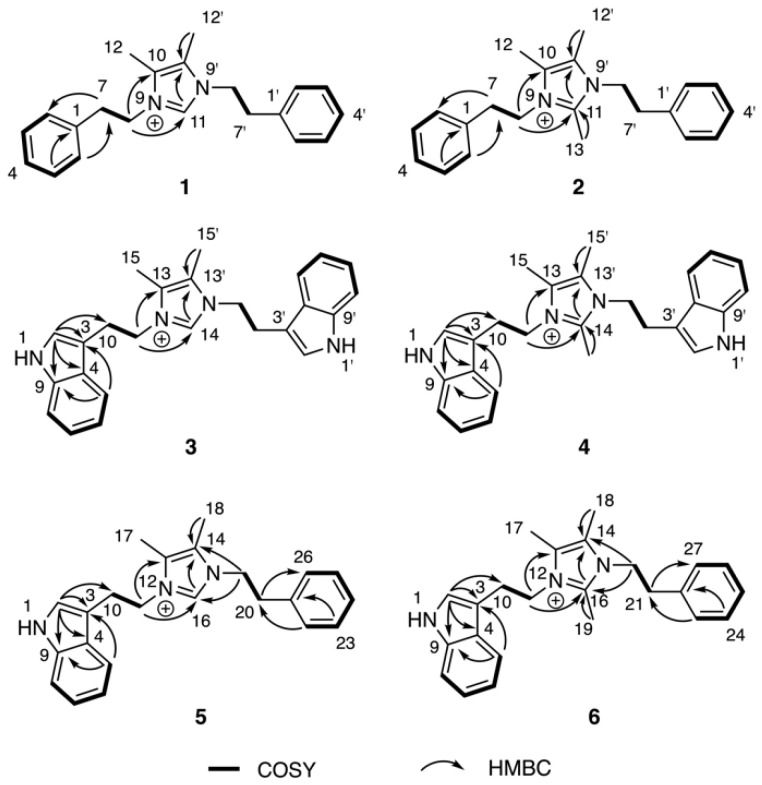
^1^H–^1^H COSY and key HMBC correlations of compounds **1**–**6**.

The molecular formulae of bacillimidazoles A (**1**) and B (**2**) were determined to be C_21_H_25_N_2_^+^ (*m*/*z* = 305.2029, M^+^, calcd 305.2012, Figure S37) and C_22_H_27_N_2_^+^ (*m*/*z* = 319.2171, M^+^, calcd 319.2169, Figure S38), respectively, based on HRESIMS data. In the ^13^C NMR spectra of **1** and **2** in Table 1, only 11 and 12 carbon signals were observed, respectively, suggesting symmetric scaffolds for both **1** and **2**. Furthermore, comparisons of ^1^H and ^13^C NMR data revealed a high degree of similarity between compound **1** and lepidiline A, an imidazolium-containing alkaloid isolated from the South American plants *Lepidium meyenii* Walp [22]. These similarities suggested the presence of a 4,5-dimethyl imidazolium cyclic structure and two phenyl-ring containing substituents in compound **1**. More highly refined datasets revealed that H_2_-7 (*δ*_H_ 3.06) and H_2_-8 (*δ*_H_ 4.35) showed COSY correlations (Figure 3) to each other. The HMBC correlations (Figure 3) were also observed from H_2_-7 to C-2 (*δ*_C_ 130.0) and from H_2_-8 (*δ*_H_ 4.35) to C-10 (*δ*_C_ 128.5), C-11 (*δ*_C_ 135.5), suggesting that –CH_2_CH_2_– groups linked the central imidazolium ring to two terminal phenyl rings, one on either side of the imidazolium. Therefore, the structure of **1** was assigned as a 1,3-difunctionalized imidazolium-containing structure. The NMR dataset of **2** was compared to that obtained for **1** and the only observable difference was an additional methyl group (*δ*_H_, 2.01, H_3_-13; *δ*_C_ 9.7, CH_3_) substitution on C-11 (*δ*_C_ 144.0), which was determined by careful interpretation of the well resolved HMBC correlation from H_3_-13 to C-11 (Figure 3).

The molecular formulae of bacillimidazole C (**3**) and bacillimidazole D (**4**) were identified as C_25_H_27_N_4_^+^ (*m*/*z* = 383.2228, M^+^, calcd 383.2230) and C_26_H_29_N_4_^+^ (*m*/*z* = 397.2385, M^+^, calcd 397.2387), respectively, by analyzing their HRMS data (Figures S39 and S40). Analysis of their ^13^C NMR data also suggested symmetrical structures for both **3** and **4**. In particular, detailed 1D NMR data analyses of **3** and **4** suggested that the central portion of each molecule bore a common imidazolium functionality. Overall, five sets of unassigned aromatic protons (H-2, *δ*_H_ 6.96; H-5, *δ*_H_ 7.32; H-6, *δ*_H_ 7.03; H-7, *δ*_H_ 7.15; H-8, *δ*_H_ 7.39) and eight unassigned aromatic carbons (C-2, *δ*_C_ 124.4; C-3, *δ*_C_ 110.5; C-4, *δ*_C_ 128.3; C-5, *δ*_C_ 118.4; C-6, *δ*_C_ 120.1; C-7, *δ*_C_ 122.8; C-8, *δ*_C_ 112.6; C-9, *δ*_C_ 138.1) were characteristic of the 3-indoyl structural motifs in **3**; the validity of this idea was verified by the observation of HMBC correlations from H-2 to C-3/C-4/C-9 and from H-5 to C-3/C-9 (Figure 3). Similar to **1** and **2**, the connection between –CH_2_CH_2_– groups and the aromatic building blocks in **3**, as well as the central imidazolium ring, was deduced by HMBC correlations from H-3 to C-10 and from H_2_-11 to C-13/C-14 (Figure 3), respectively, establishing the full structural assignment of bacillimidazole C (**3**). Finally, in a fashion similar to that applied to **3**, the structure of **4**, with its additional imidazolium-linked methyl group, was elucidated. 

Approaches employed to solve the structures of **1**–**4** also were applied to determine the structures of **5** and **6** in Table 2. Analysis of HRMS data for bacillimidazole E (**5**) and bacillimidazole F (**6**) made clear their molecular formulae as C_23_H_26_N_3_^+^ (*m*/*z* = 344.2134, M^+^, calcd 344.2121) and C_24_H_28_N_3_^+^ (*m*/*z* = 358.2283, M^+^, calcd 358.2278), respectively. In addition, review of their ^13^C NMR data suggested asymmetrical structures for both **5** and **6** since 23 ^13^C NMR signals were observed in **5** (24 signals for **6**). Additionally, comparisons of NMR data for **5** to those of **1** and **3** suggested the presence of an imidazolium ring, a phenyl ring, and a 3-indole ring in **5**. Furthermore, H-2 (*δ*_H_ 7.03), assigned to the indole ring, showed an HMBC correlation to C-10 (*δ*_H_ 26.5); and H-22 (*δ*_H_ 7.04), assigned to the phenyl ring, showed an HMBC correlation to C-20 (*δ*_C_ 36.7) (Figure 3). COSY correlations were observed between H_2_-10 (*δ*_H_ 3.23) and H_2_-11 (*δ*_H_ 4.36), H_2_-19 (*δ*_H_ 4.18) and H_2_-20 (*δ*_H_ 2.84). H_2_-11 and H_2_-19 both showed HMBC correlations to C-16 (*δ*_C_ 135.4), which was assigned to the imidazolium ring (Figure 3). Therefore, **5** was believed to contain an indole ring, an imidazolium ring, and a phenyl moiety (Figure 2). In addition, two –CH_2_CH_2_– groups were found to intervene the three different cyclic structural motifs. In applying the established correlation data, we thus elucidated structure **5** as shown in Figure 2. The structure of **6** was determined in a fashion similar to that employed for **5**, and was ultimately identified as an analog of **5** bearing a methyl group on the central imidazolium ring.

Following their structural elucidation, bacillimidazoles A–F (**1**–**6**) were tested for antibacterial activity against MRSA, *B. subtilis,* and *E. coli* (Table S1). All compounds failed to show any significant activity against *B. subtilis* and *E. coli*, although bacillimidazole F (**6**) did display weak activity against MRSA with an MIC of 38.3 μM.

**Figure 4:**
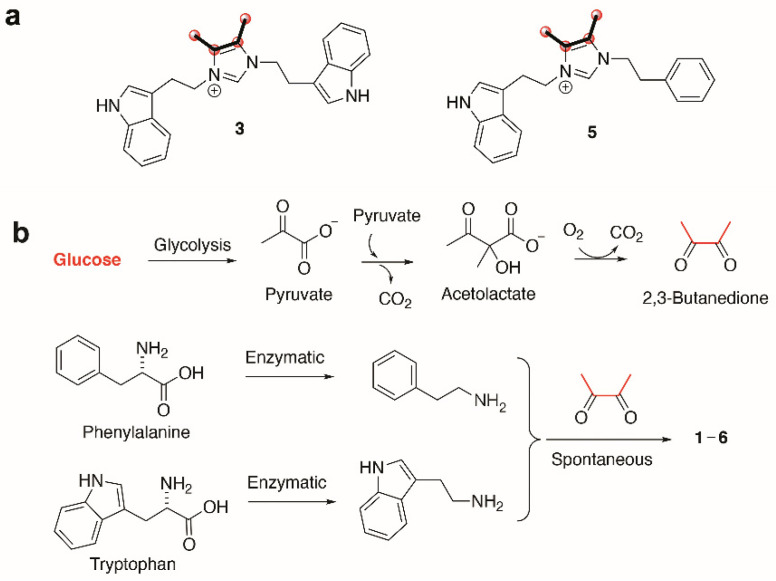
Biosynthesis of bacillimidazoles. (**a**) Isotopic labeling of bacillimidazoles C (**3**) and E (**5**). Carbons highlighted with bold bonds and red spheres showed high levels of ^13^C incorporation. (**b**) Proposed biosynthetic pathway to compounds **1**–**6** calls for enzymatic production of tryptamine, phenethylamine and 2,3-butanedione (from glucose); all subsequent steps may proceed spontaneously.

To better understand the biosynthetic mechanisms involved in generating the uncommon imidazolium structures found in the bacillimidazoles, isotopic enrichment studies were carried out. In particular, we employed ^13^C enriched culture media to glean vital insight into how the bacillimidazoles are constructed. By substituting two carbon sources, starch and d-glucose, with ^13^C_6_-d-glucose, we found that the four carbons composing the vicinal dimethyl olefin of bacillimidazoles C (**3**) and E (**5**) (Figures S43–S48) underwent substantial ^13^C enrichment relative to all other carbons (Figure 4a). On the basis of these findings, we theorized that all carbons of the basic framework originate from amino acids whereas the dimethyl olefin elements of the bacillimidazoles are derived from glucose, presumably via a glycolytic process (Figure 4b). This logic is, of course, buoyed by the resemblance of the bacillimidazole sidechains to those found in phenylalanine and tryptophan. On the basis of these findings, we investigated the possibility of a biosynthetic pathway, as shown in Figure 4. It is well known that glucose is readily converted to 2,3-butadione along the canonical glycolytic pathway to acetolactate and subsequent processing of acetolactate to the essential 2,3-butadione, which can spontaneously react with either tryptamine or phenethylamine, both of which are common bacterial metabolites formed from the aromatic amino acids tryptophan and phenylalanine. These three components—two amines and a dione—provide most of the atoms of the imidazole moiety tethered through both nitrogens to various side chains. This diimine could undergo further spontaneous reactions with either one or two carbon carboxylic acids, or derivatives, and appropriate redox agents (or enzymes) to form the central imidazolium ring. Acetyl-CoA, or an equivalent, would produce the methylated compounds and formate, or a derivative, would produce the unmethylated bacillimidazoles. There is an alternative possibility in which the relevant amino acids condense with the butadione moiety prior to decarboxylation, and that the decarboxylation(s) of such intermediates might expedite imidazolium ring formation. While it is not possible at this stage to propose a detailed stepwise biosynthesis for the bacillimidazoles, an overall path with both enzymatic and spontaneous steps is likely, and the ^13^C-glucose feeding studies clearly indicate the importance of glucose processing en route to imidazole assembly. That the production of bacillimidazoles by WMMC1349 is driven by secondary metabolism biosynthetic machineries and is not relegated only to primary metabolic events and/or extract workup conditions, is supported by the fact that, of six *bacilli* strains evaluated, only two (including WMMC1349) proved to be bacillimidazole producers (Figures S4–S6, S49–S54 and Table S2).

Based on the biosynthetic insights gained from labelling experiments, we set out to identify the gene cluster responsible for the biosynthesis of **1**–**6**. We sequenced and assembled the genome of the producer, *Bacillus* sp. WMMC1349 [23]. The genome sequence was analyzed with state-of-the-art biosynthetic pipelines, yet no likely biosynthetic pathway for the biosynthesis of **1**–**6** was identified. We therefore mined the genome for the presence of the genes involved in acetoin biosynthesis, of which 2,3-butanedione, the proposed building block of **1**–**6**, is a precursor. We identified all of the genes responsible for acetoin biosynthesis in the genome of *Bacillus* sp. WMMC1349. Acetoin is biosynthesized from two molecules of pyruvate that are condensed to generate acetolactate. Acetolactate can either be enzymatically transformed into acetoin by a decarboxylase or can undergo spontaneous decarboxylation to yield the building block of **1**–**6**, 2,3-butanedione. A diacetyl reductase subsequently converts 2,3-butanedione into acetoin. Whole genome alignments of different *Bacillus* spp. revealed that the region upstream of the acetoin biosynthetic genes differs significantly from other *Bacillus* spp., including the model strain *B. subtilis* 168 (insertion of a large low-density coding region in *Bacillus* sp. WMMC1349), while the downstream region is homologous in all analyzed genomes. To our surprise, we were not able to identify a copy of the gene family encoding butanediol-dehydrogenases which was present in all other analyzed *Bacillus* spp. The absence of genes involved in acetoin catabolism would be expected to increase the concentration of the precursor 2,3-butanedione. Genome mining (Section 4.5 below) of the bacillimidazole producer revealed the presence of a gene encoding an aromatic-l-amino acid decarboxylase, which is the proposed second enzyme essential for **1**–**6** biosynthesis en route to building blocks tryptamine and phenethylamine, respectively. No homologs of the aromatic-l-amino acid decarboxylase gene were identified in any other *Bacillus* genome analyzed, including the model strain *Bacillus subtilis* 168, indicating that the gene is facultative for the genus *Bacillus*. Relative to the biosynthetic machineries of most bacterial secondary metabolites, genes involved in acetoin biosynthesis and the aromatic-l-amino acid decarboxylase gene are not clustered. This observation suggests that the bacillimidazoles may form spontaneously from high-abundance primary metabolites with congruent reactivities. The biosynthesis of the bacillimidazoles is yet another example of the growing number of natural products that are produced partly by genetically coded instructions and partly by spontaneous reactivity—the joining of reactive intermediates, or products, from different pathways [24,25,26]. The initial formation of the bis-imines represents, for example, the unsurprising coupling of highly reactive primary amines with a very reactive alpha-diketone.

## 3. Conclusions

The isolation and structural elucidation of bacillimidazoles A–F, six imidazolium-containing heterocycles from marine *Bacillus* sp. WMMC1349 represent the discovery of a new class of marine-derived heterocyclic natural products. Isotopic labeling of **1**–**6** using ^13^C enriched culture media, biomimetic synthetic approaches and bioinformatic analyses were performed in order to gain insights into the biosynthetic assembly of these interesting compounds. To the best of our knowledge, there are only a few reports of natural products containing 1,3-difunctionalized imidazolium moieties, and this is the first report of naturally occurring imidazolium-containing heterocycles. We contend, based on these findings, that the discovery of **1**–**6** contributes to our advancing knowledge of significantly underexplored biosynthetic pathways, specifically those that intersect primary and secondary metabolic pathways. We anticipate that the lessons learned here will help to expedite efforts to more fully understand and exploit the full biosynthetic potential of *Bacillus* spp.

## 4. Materials and Methods

### 4.1. General Experimental Procedures

UV spectra were recorded on an Aminco/OLIS UV-Vis spectrophotometer. IR spectra were measured with a Bruker Equinox 55/S FT–IR spectrophotometer. NMR spectra were obtained in CD_3_OD (*δ*_H_ 3.34 ppm, *δ*_C_ 49.0 ppm) with a Bruker Avance 600 III MHz (Billerica, MA, USA) spectrometer equipped with a ^1^H{^13^C/^15^N/^31^P} cryoprobe, a Bruker Avance III 500 MHz (Billerica, MA, USA) spectrometer equipped with a ^13^C/^15^N{^1^H} cryoprobe, and a Bruker Avance III HD 400 MHz (Billerica, MA, USA) spectrometer. HRMS data were acquired with a Bruker MaXis™ 4G ESI-QTOF (Billerica, MA, USA) mass spectrometer. RP HPLC was performed using a Shimadzu Prominence HPLC system and a Phenomenex Gemini C18 column (250 × 30 mm). UHPLC-HRMS was acquired using a Bruker MaXis™ 4G ESI-QTOF (Billerica, MA, USA) mass spectrometer coupled with a Waters Acquity UPLC system operated by Bruker Hystar software and a C18 column (Phenomenex Kinetex 2.6 μm, 2.1 mm × 100 mm). Bruker timsTOF Pro instrument (Billerica, MA, USA) was used for the trapped ion mobility MS analysis using direct infusion with 0.003 mL/min of flow rate and ESI+ ionization source. Nebulizer gas 0.4 bar, dry gas 3.5 L/min, source temperature 220 °C, ESI voltage 4200V (+). MS spectra were collected using the following parameters: tims ramp time = 350 ms, PASEF on, scan range (*m*/*z*, 20–1000; 1/k_0_, 0.70–1.00 V·s/cm^2^. 

### 4.2. Biological Material

Sponge specimens were collected on 27 May 2015 near the west shore of Ramrod Key (24°39′38.1″ N, 81°25′25.0″ W) in Florida. A voucher specimen is housed at the University of Wisconsin−Madison. For cultivation, a sample of sponge (1 cm^3^) was ground in 500 μL sterile seawater, and dilutions were made using 500 μL sterile seawater. Subsequently, 400 μL of diluted sponge sample was added to 200 μL of sterile artificial seawater and 100 μL was plated using a sterile L-shaped spreader. Diluted samples were plated on Gauze 1 media supplemented with artificial seawater. Each medium was supplemented with 50 μg/mL cycloheximide, 25 μg/mL nystatin, and 25 μg/mL nalidixic acid. Plates were incubated at 28 °C and colonies were isolated over the course of two months.

### 4.3. Fermentation, Extraction and Isolation

Two 10 mL seed cultures (25 × 150 mm tubes) in medium DSC (20 g soluble starch, 10 g glucose, 5 g peptone, 5 g yeast extract per liter of artificial seawater) were inoculated with strain WMMC-1349 and shaken (200 RPM, 28 °C) for seven days. Two-liter flasks (1 × 500 mL) containing ASW-A (20 g soluble starch, 10 g d-glucose, 5 g peptone, 5 g yeast extract, 5 g CaCO_3_ per liter of artificial seawater. For ^13^C enriched ASW-A media, 10 g d-glucose (U-^13^C_6_, 99%) was used instead of soluble starch and d-glucose) were inoculated with 20 mL seed culture and were incubated (200 RPM, 28 °C) for seven days. Four-liter flasks (10 × 1 L) containing medium ASW-A with Diaion HP20 (7% by weight) were inoculated with 50 mL from the 500 mL culture and shaken (200 RPM, 28 °C) for seven days. For producing artificial sea water, solutions I (415.2 g NaCl, 69.54 g Na_2_SO_4_, 11.74 g KCl, 3.40 g NaHCO_3_, 1.7 g KBr, 0.45 g H_3_BO_3_, 0.054 g NaF) and II (187.9 g MgCl_2_·6H_2_O, 22.72 g CaCl_2_·2H_2_O, 0.428 g SrCl_2_·6H_2_O) were made up separately using distilled water and combined to give a total volume of 20 L.

Filtered HP20 and cells were washed with H_2_O and extracted with acetone. The acetone extract was subjected to liquid-liquid partitioning using 30% aqueous MeOH and CHCl_3_ (1:1). The CHCl_3_-soluble partition (3.12 g) was fractionated by Sephadex LH20 column chromatography (column size 500 × 40 mm, CHCl_3_:MeOH = 1:1, 20 mL for each fraction). Fractions containing **1**–**6** (1.1 g) were subjected to RP HPLC (20%/80% to 100%/0% MeOH/H_2_O (with 0.1% acetic acid), 23.5 min, 20 mL/min) using a Phenomenex Gemini C18 column (250 × 30 mm). The fraction collected between 16–18 min was further fractionated by RP HPLC (22%/78% to 51%/49% MeCN/H_2_O (with 0.05% trifluoroacetic acid), 29.5 min, 20 mL/min) using a Phenomenex Gemini C18 column (250 × 30 mm), yielding **1** (50.2 mg, *t*_R_ 28.2 min), **2** (35.8 mg, *t*_R_ 27.6 min), **3** (2.2 mg, *t*_R_ 27.3 min), **4** (3.1 mg, *t*_R_ 28.9 min), **5** (40.5 mg, *t*_R_ 28.6 min), **6** (32.3 mg, *t*_R_ 29.3 min).

Bacillimidazole A (**1**): light yellow solid, UV-Vis (MeOH): λ_max_ (log ε) 211 nm (3.83), 259 nm (2.65), 279 nm (2.55), 291 nm (2.55); IR (ATR): υ_max_ 3385, 3144, 3033, 2935, 2873, 2834, 1781, 1679, 1563, 1498, 1456, 1399, 1357, 1199, 1129, 1083, 1029, 831, 801, 751, 720, 702 cm^−1^; HRMS M^+^ *m*/*z* = 305.2029 (calcd. for C_21_H_25_N_2_^+^ 305.2012). ^1^H NMR (600 MHz, MeOD) *δ*_H_ 8.51 (s, 1H), 7.34 (t, *J* = 7.2 Hz, 4H), 7.31 (d, *J* = 7.2 Hz, 2H), 7.12 (d, *J* = 7.0 Hz, 4H), 4.35 (t, *J* = 7.0 Hz, 4H), 3.06 (t, *J* = 6.9 Hz, 4H), 2.08 (s, 6H). ^13^C NMR (126 MHz, MeOD) *δ*_C_ 137.83, 135.53, 130.02, 129.97, 128.45, 128.44, 49.28, 37.07, 7.94.

Bacillimidazole B (**2**): light yellow solid, UV-Vis (MeOH): λ_max_ (log ε) 217 nm (3.85), 274 nm (3.02), 282 nm (3.04), 290 (2.97); IR (ATR): υ_max_ 3383, 3065, 3034, 2934, 2870, 1681, 1564, 1525, 1497, 1440, 1400, 1355, 1200, 1128, 1030, 934, 840, 801, 749, 722, 703 cm^−1^; HRMS M^+^ *m*/*z* = 319.2171 (calcd. for C_22_H_27_N_2_^+^ 319.2169). ^1^H NMR (600 MHz, MeOD) *δ*_H_ 7.36–7.33 (m, 6H), 7.11 (d, *J* = 6.4 Hz, 4H), 4.31 (t, *J* = 6.7 Hz, 4H), 3.02 (t, *J* = 6.7 Hz, 4H), 2.08 (s, 6H), 2.01 (s, 3H). ^13^C NMR (126 MHz, MeOD) *δ*_C_ 144.04, 138.15, 130.22, 130.08, 128.56, 127.14, 47.87, 36.33, 9.67, 8.21.

Bacillimidazole C (**3**): light yellow solid, UV-Vis (MeOH): λ_max_ (log ε) 223 nm (4.25), 274 nm (3.67), 282 nm (3.69), 290 nm (3.61); IR (ATR): υ_max_ 3357, 2946, 2835, 1679, 1564, 1449, 1432, 1341, 1203, 1185, 1137, 1024, 838, 802, 746, 722 cm^−1^; HRMS M^+^ *m*/*z* = 383.2228 (calcd. for C_25_H_27_N_4_^+^ 383.2230). ^1^H NMR (600 MHz, MeOD) *δ*_H_ 8.12 (s, 1H), 7.39 (d, *J* = 8.1 Hz, 2H), 7.32 (d, *J* = 7.9 Hz, 2H), 7.15 (t, *J* = 7.4 Hz, 2H), 7.03 (t, *J* = 7.4 Hz, 2H), 6.96 (s, 2H), 4.25 (t, *J* = 6.7 Hz, 4H), 3.07 (t, *J* = 6.7 Hz, 4H), 2.09 (s, 9H). ^13^C NMR (126 MHz, MeOD) *δ*_C_ 138.05, 135.37, 128.30, 128.19, 124.42, 122.83, 120.13, 118.43, 112.64, 110.49, 26.81, 7.94.

Bacillimidazole D (**4**): light yellow solid, UV-Vis (MeOH): λ_max_ (log ε) 224 nm (3.98), 281 nm (3.44), 290 nm (3.38); IR (ATR): υ_max_ 3360, 3292, 2925, 2854, 1729, 1648, 1561, 1456, 1411, 1342, 1256, 1235, 1181, 1105, 1073, 1025, 926, 744 cm^−1^; HRMS M^+^ *m*/*z* = 397.2385 (calcd. for C_26_H_29_N_4_^+^ 397.2387). ^1^H NMR (500 MHz, MeOD) *δ*_H_ 7.39 (d, *J* = 8.0 Hz, 1H), 7.24 (d, *J* = 7.9 Hz, 1H), 7.14 (t, *J* = 7.5 Hz, 1H), 7.03 (t, *J* = 7.5 Hz, 1H), 6.98 (s, 1H), 4.19 (t, *J* = 5.9 Hz, 2H), 3.03 (t, *J* = 5.7 Hz, 2H), 2.18 (s, 3H), 1.64 (s, 1H). ^13^C NMR (126 MHz, MeOD) *δ*_C_ 143.95, 138.00, 128.40, 126.96, 124.50, 122.88, 120.21, 118.23, 112.67, 110.76, 47.50, 25.97, 9.30, 8.29.

Bacillimidazole E (**5**): light yellow solid, UV-Vis (MeOH): λ_max_ (log ε) 221 nm (3.87), 274 nm (3.17), 282 nm (3.18), 290 (3.11); IR (ATR): υ_max_ 3376, 2991, 2950, 2836, 1677, 1564, 1497, 1456, 1398, 1356, 1341, 1201, 1134, 1078, 1025, 934, 834, 801, 747, 721, 703 cm^−1^; HRMS M^+^ *m*/*z* = 344.2134 (calcd. for C_23_H_26_N_3_^+^ 344.2121). ^1^H NMR (600 MHz, MeOD) *δ*_H_ 8.27 (s, 1H), 7.40 (d, *J* = 8.2 Hz, 1H), 7.34 (d, *J* = 7.9 Hz, 1H), 7.32–7.26 (m, 3H), 7.15 (t, *J* = 7.4 Hz, 1H), 7.07–7.02 (m, 4H), 4.38 (t, *J* = 6.5 Hz, 2H), 4.18 (t, *J* = 7.2 Hz, 2H), 3.23 (t, *J* = 6.5 Hz, 2H), 2.84 (t, *J* = 7.2 Hz, 2H), 2.12 (s, 3H), 2.04 (s, 3H). ^13^C NMR (126 MHz, MeOD) *δ*_C_ 138.08, 137.82, 135.44, 129.95, 129.87, 128.38, 128.34, 128.29, 128.27, 124.55, 122.85, 120.17, 118.37, 112.68, 110.42, 37.03, 26.76, 7.97, 7.89.

Bacillimidazole F (**6**): light yellow solid, UV-Vis (MeOH): λ_max_ (log ε) 225 nm (3.96), 274 nm (3.55), 282 nm (3.56), 290 nm (3.5^1^); IR (ATR): υ_max_ 3356, 3275, 3062, 3001, 2971, 2928, 2830, 1647, 1562, 1497, 1454, 1401, 1353, 1234, 1203, 1179, 1108, 1077, 1028, 924, 745, 703 cm^−1^; HRMS M^+^ *m/z* = 358.2283 (calcd. for C_24_H_28_N_3_^+^ 358.2278). ^1^H NMR (500 MHz, MeOD) *δ*_H_ 7.37 (d, *J* = 8.0 Hz, 1H), 7.28 (m, 3H), 7.23 (d, *J* = 7.9 Hz, 1H), 7.11 (t, *J* = 7.5 Hz, 1H), 7.06 – 6.97 (m, 3H), 4.31 (t, *J* = 5.5 Hz, 2H), 4.11 (t, *J* = 6.6 Hz, 2H), 3.17 (t, *J* = 5.5 Hz, 2H), 2.76 (t, *J* = 6.6 Hz, 2H), 2.15 (s, 2H), 2.07 (s, 3H), 1.77 (s, 3H). ^13^C NMR (126 MHz, MeOD) *δ*_C_ 143.99, 138.09, 138.03, 130.07, 130.02, 128.47, 128.45, 127.06, 127.00, 124.64, 122.91, 120.28, 118.18, 112.72, 110.76, 47.74, 47.59, 36.27, 26.00, 9.43, 8.32, 8.18.

### 4.4. Antibacterial Testing

Bacillimidazoles A–F (**1**–**6**) were tested for antibacterial activity against *E. coli* (ATCC #25922), *B. subtilis* strain NRS-231, and Methicillin-resistant *Staphylococcus aureus* (MRSA) (ATCC #33591), and MICs were determined using a dilution antimicrobial susceptibility test for aerobic bacteria. Compounds **1**–**6** were dissolved in DMSO and serially diluted to 10 concentrations (0.25–128 μg/mL) in 96-well plates. Vancomycin was used as a positive control against *B. subtilis* and MRSA, and exhibited MIC values of 0.25 μg/mL. Gentamicin was used as a positive control against *E. coli*, and exhibited an MIC of 4 μg/mL. Bacillimidazoles, vancomycin, and gentamicin were tested in triplicate. On each plate, there were six untreated media controls. The plates were incubated at 37 °C for 18 h. The MICs were determined as the lowest concentration that inhibited visible growth of bacteria.

### 4.5. Sequencing and Identification of Candidate Bacillimidazole Biosynthetic Genes

16S rDNA sequencing was conducted as previously described [27]. WMMC1349 was identified as a *Bacillus* sp. The 16S sequence for WMMC1349 was deposited in GenBank (accession number MK892477). PacBio sequencing data were converted from BAM to FASTQ format using bedtools [28], and this fastq file was then corrected, trimmed, and assembled using Canu v1.8 [29], with an estimated genome size of 4.5 megabases (Mb). The resulting assembly was 4.677Mb over 10 contigs. It has an N50 of 4.03Mb and an L50 of 1.

Genome sequence (accession number JABJUQ000000000) was subjected to antiSMASH 5.0 analysis [30]. Results were analyzed by BLAST analysis. Acetoin and amino acid biosynthetic genes (KEGG) were identified in the bacillimidazole producer and selected model *Bacillus* spp. By BLAST analysis using Geneious 11.1.3 [31], and verified using additional online platforms such as Phyre2 [32]. Genomes of the producer of **1**–**6** and selected model *Bacillus* spp. were aligned using the MAUVE algorithm [33]. Promoter regions were identified using the BPROM algorithm [34].

## Figures and Tables

**Table 1:** Summary of ^1^H and ^13^C NMR data for **1**–**4** (600 MHz for ^1^H (500 MHz for **4**), 125 MHz for ^13^C, CD_3_OD).

Position	1		2		3		4	
	*δ*_C_, Type	*δ*_H_, (*J* in Hz)	*δ*_C_, Type	*δ*_H_, (*J* in Hz)	*δ*_C_, Type	*δ*_H_, (*J* in Hz)	*δ*_C_, Type	*δ*_H_, (*J* in Hz)
1, 1′	137.8, qC		138.2, qC					
2, 2′	130.0, CH	7.12, dd (8.0, 1.8)	130.2, CH	7.11, dd (8.0, 1.8)	124.4, CH	6.96, s	124.5, CH	6.98, s
3, 3′	130.0, CH	7.34, t (7.4)	130.1, CH	7.34, t (7.8)	110.5, qC		110.8, qC	
4, 4′	128.4, CH	7.30, t (7.4)	128.6, CH	7.32, t (7.8)	128.3, qC		128.4, qC	
5, 5′	130.0, CH	7.34, t (7.4)	130.1, CH	7.34, t (7.8)	118.4, CH	7.32, d (7.6)	118.2, CH	7.24, d (8.0)
6, 6′	130.0, CH	7.12, dd (8.0, 1.8)	130.2, CH	7.11, dd (8.0, 1.8)	120.1, CH	7.03, t (7.6)	120.2, CH	7.03, t (8.0)
7, 7′	37.1, CH_2_	3.06, t (6.6)	36.3, CH_2_	3.02, t (6.8)	122.8, CH	7.15, t (7.6)	122.9, CH	7.14, t (8.0)
8, 8′	49.3, CH_2_	4.35, t (6.6)	47.9, CH_2_	4.31, t (6.8)	112.6, CH	7.39, d (7.8)	112.7, CH	7.39, d (8.0)
9, 9′					138.1, qC		138.0, qC	
10, 10′	128.5, qC		127.1, qC		26.8, CH_2_	3.07, t (6.8)	26.0, CH_2_	3.03, t (6.0)
11, 11′	135.5 CH	8.51, s	144.0, qC		48.7, CH_2_	4.25, t (6.8)	47.5, CH_2_	4.19, t (6.0)
12, 12′	7.9, CH_3_	2.08, s	8.2, CH_3_	2.08, s				
13, 13′			9.7, CH_3_	2.01, s	128.2, qC		127.0, qC	
14					135.4, CH	8.12, s	144.0, qC	
15, 15′					7.9, CH_3_	2.09, s	8.3, CH_3_	2.18, s
16							9.3, CH_3_	1.64, s

**Table 2:** Summary of ^1^H and ^13^C NMR data for **5** and **6** (600 MHz for ^1^H. (500 MHz for **6**), 125 MHz for ^13^C, CD_3_OD).

Position	5		6	
	*δ*_C_, Type	*δ*_H_, (*J* in Hz)	*δ*_C_, Type	*δ*_H_, (*J* in Hz)
1				
2	124.6, CH	7.03, s	124.6, CH	7.02, s
3	110.4, qC		110.8, qC	
4	128.4, qC		128.5, qC	
5	112.7, CH	7.40, dd (8.2, 1.0)	112.7, CH	7.37, d (7.8)
6	120.2, CH	7.04, dt (7.6, 1.2)	120.3, CH	7.00, t (7.8)
7	122.9, CH	7.15, dt (8.0, 1.0)	123.9, CH	7.12, t (7.8)
8	118.4, CH	7.34, dd (8.0, 1.0)	118.2, CH	7.23, d (7.8)
9	138.1, qC		138.1, qC	
10	26.5, CH_2_	3.23, t (6.6)	26.0, CH_2_	3.17, t (6.2)
11	48.9, CH_2_	4.38, t (6.6)	47.7, CH_2_	4.30, t (6.2)
12				
13	128.3, qC		127.1, qC	
14	128.3, qC		127.0, qC	
15				
16	135.4, CH	8.27, s	144.0, qC	
17	8.0, CH_3_	2.12, s	8.3, CH_3_	2.15, s
18	7.9, CH_3_	2.04, s	8.2, CH_3_	2.07, s
19	48.7, CH_2_	4.18, t (7.2)	9.4, CH_3_	1.77, s
20	36.7, CH_2_	2.84, t (7.2)	47.6, CH_2_	4.11, t (6.8)
21	137.8, qC		36.3, CH_2_	2.77, t (6.8)
22	130.0, CH	7.04, d (7.2)	138.0, qC	
23	129.9, CH	7.29, m	130.1, CH	7.03, dd (7.3, 1.6)
24	128.3, CH	7.28, m	130.0, CH	7.30, m
25	129.9, CH	7.29, m	128.5, CH	7.26, m
26	130.0, CH	7.04, d (7.2)	130.0, CH	7.30, m
27			130.1, CH	7.03, dd (7.3, 1.6)

## Supplementary Materials

The following are available online at https://www.mdpi.com/article/10.3390/md20010043/s1. Detailed experimental discussions of isotopic enrichment studies and validation of bacillimidazoles as genuine natural products as well as Tables S1 & S2 summarizing all antibacterial data (Table S1), and bacillimidazole productivity of six *Bacilli* strains (Table S2). Also: Figure S1. Analytical HPLC analysis and ^1^H NMR analysis of active subfractions. Figure S2. LC-MS analysis (EIC *m/z* 383) of 3. Figure S3. LC-MS analysis (EIC *m/z* 397) of 4. Figure S4. LC-MS analysis of chemical reaction of tryptamine (10.0 mg/mL) and 2,3 butanedione (0.5 equivalent) in MeOH solution without HP-20 resin for 2 h. Figure S5. LC-MS analysis of chemical reaction of tryptamine (10.0 mg/mL) and 2,3 butanedione (0.5 equivalent) in MeOH solution with HP-20 resin for 2 h. Figure S6. LC-MS analysis of chemical reaction of tryptamine (10.0 mg/mL) and 2,3 butanedione (0.5 equivalent) in CHCl_3_:MeOH = 1:1 solution for 24 h. Figure S7. ^1^H NMR Spectrum of Bacillimidazole A (1, 600 MHz, CD_3_OD). Figure S8. ^13^C NMR Spectrum of Bacillimidazole A (1, 125 MHz, CD_3_OD). Figure S9. gCOSY Spectrum of Bacillimidazole A (1, 600 MHz, CD_3_OD). Figure S10. gHSQC Spectrum of Bacillimidazole A (1, 600 MHz, CD_3_OD). Figure S11. gHMBC Spectrum of Bacillimidazole A (1, 600 MHz, CD_3_OD). Figure S12. ^1^H NMR Spectrum of Bacillimidazole B (2, 600 MHz, CD_3_OD). Figure S13. ^13^C NMR Spectrum of Bacillimidazole B (2, 125 MHz, CD_3_OD). Figure S14. gCOSY Spectrum of Bacillimidazole B (2, 600 MHz, CD_3_OD). Figure S15. gHSQC Spectrum of Bacillimidazole B (2, 600 MHz, CD_3_OD). Figure S16. gHMBC Spectrum of Bacillimidazole B (2, 600 MHz, CD_3_OD). Figure S17. ^1^H NMR Spectrum of Bacillimidazole C (3, 600 MHz, CD_3_OD). Figure S18. ^13^C NMR Spectrum of Bacillimidazole C (3, 125 MHz, CD_3_OD). Figure S19. gCOSY Spectrum of Bacillimidazole C (3, 600 MHz, CD_3_OD). Figure S20. gHSQC Spectrum of Bacillimidazole C (3, 600 MHz, CD_3_OD). Figure S21. gHMBC Spectrum of Bacillimidazole C (3, 600 MHz, CD_3_OD). Figure S22. ^1^H NMR Spectrum of Bacillimidazole D (4, 500 MHz, CD_3_OD). Figure S23. ^13^C NMR Spectrum of Bacillimidazole D (4, 125 MHz, CD_3_OD). Figure S24. gCOSY Spectrum of Bacillimidazole D (4, 500 MHz, CD_3_OD). Figure S25. gHSQC Spectrum of Bacillimidazole D (4, 500 MHz, CD_3_OD). Figure S26. gHMBC Spectrum of Bacillimidazole D (4, 500 MHz, CD_3_OD). Figure S27. ^1^H NMR Spectrum of Bacillimidazole E (5, 600 MHz, CD_3_OD). Figure S28. ^13^C NMR Spectrum of Bacillimidazole E (5, 125 MHz, CD_3_OD). Figure S29. gCOSY Spectrum of Bacillimidazole E (5, 600 MHz, CD_3_OD). Figure S30. gHSQC Spectrum of Bacillimidazole E (5, 600 MHz, CD_3_OD). Figure S31. gHMBC Spectrum of Bacillimidazole E (5, 600 MHz, CD_3_OD). Figure S32. ^1^H NMR Spectrum of Bacillimidazole F (6, 500 MHz, CD_3_OD). Figure S33. ^13^C NMR Spectrum of Bacillimidazole F (6, 125 MHz, CD_3_OD). Figure S34. gCOSY Spectrum of Bacillimidazole F (6, 500 MHz, CD_3_OD). Figure S35. gHSQC Spectrum of Bacillimidazole F (6, 500 MHz, CD_3_OD). Figure S36. gHMBC Spectrum of Bacillimidazole F (6, 500 MHz, CD_3_OD). Figure S37. Positive Ion HRESIMS of Bacillimidazole A (1). Figure S38. Positive Ion HRESIMS of Bacillimidazole B (2). Figure S39. Positive Ion HRESIMS of Bacillimidazole C (3). Figure S40. Positive Ion HRESIMS of Bacillimidazole D (4). Figure S41. Positive Ion HRESIMS of Bacillimidazole E (5). Figure S42. Positive Ion HRESIMS of Bacillimidazole F (6). Figure S43. ^1^H NMR Spectrum of ^13^C Labeled Bacillimidazole C (3, 500 MHz, CD_3_OD). Figure S44. ^13^C NMR Spectrum of ^13^C Labeled Bacillimidazole C (3, 125 MHz, CD_3_OD). Figure S45. ^1^H NMR Spectrum of ^13^C Labeled Bacillimidazole E (5, 500 MHz, CD_3_OD). Figure S46. ^13^C NMR Spectrum of ^13^C Labeled Bacillimidazole E (5, 125 MHz, CD_3_OD). Figure S47. Positive Ion HRESIMS of ^13^C Enriched Bacillimidazole C (3). Figure S48. Positive Ion HRESIMS of ^13^C Enriched Bacillimidazole E (5). Figure S49. LC-MS analysis of strain *Bacillus* sp. WMMC325. Figure S50. LC-MS analysis of strain *Bacillus* sp. WMMC331. Figure S51. LC-MS analysis of strain *Bacillus* sp. WMMC1349. Figure S52. LC-MS analysis of strain *Bacillus* sp. WMMC1350. Figure S53. LC-MS analysis of strain *Bacillus* sp. WMMC1351. Figure S54. LC-MS analysis of strain *Bacillus* sp. WMMC1352. Figure S55. Culture broth of six different marine *Bacillus* strains. Figure S56. Pictures for sponge hosts of six different marine *Bacillus* strains.
