# Supplementary material for: Bacillimidazoles A−F, Imidazolium-Containing Compounds Isolated from a Marine Bacillus
Source: Mar Drugs. 2022 Jan 1;20(1):43. doi: 10.3390/md20010043 (PMC8779896; doi:10.3390/md20010043)
Supplement: Supplementary file 1 [file marinedrugs-20-00043-s001.zip › marinedrugs-1520632-supplementary.pdf]

# Supporting Information

## Bacillimidazoles A−F, Imidazolium-Containing Compounds Isolated from a Marine *Bacillus*


**Jia-Xuan Yan [1†,‡] , Qihao Wu [1,†,‡], Eric J. N. Helfrich[2,†], Marc G. Chevrette [3,†] , Doug R. Braun [1], Heino Heyman [4,†], Gene E. Ananiev [5], Scott R. Rajski [1], Cameron R. Currie [3], Jon Clardy [2] and Tim S. Bugni [1,5,*]**

[1] Pharmaceutical Sciences Division, University of Wisconsin-Madison, 777 Highland Ave, Madison, WI, 53705-2222, United States

[†] Current addresses: Merck & Co. LLC. 126 E. Lincoln Ave, Rahway, NJ, 07065, United States (J.-X.Y.); Departments of Chemistry & Microbial Pathogenesis, Institute of Biomolecular Design & Discovery, Yale University, West Haven, CT, 06516-7392, United States (Q.W.)

[2] Harvard Medical School, Harvard University, 240 Longwood Ave, Boston, MA, 02115, United States

[†] Current address (E.J.N.H.): Institute of Molecular Bioscience at Goethe University Frankfurt & LOEWE Centre for Translational Biodiversity Genomics, Frankfurt am Main, Germany

[3] Department of Bacteriology, University of Wisconsin-Madison, 1550 Linden Ave, Madison, WI, 53706, United States

[†] Current address (M.G.C.): Wisconsin Institute for Discovery and Department of Plant Pathology, University of Wisconsin-Madison, 425 G Henry Mall, Madison, WI, 53706, United States

[4] Bruker Daltonics, Bruker Scientific LLC., 40 Manning Rd, Billerica, MA, 01821, United States

† Current address: Metabolon Inc., 617 Davis Dr., Ste. 100, Morrisville, NC, 27560, United States

[5] The Small Molecule Screening Facility (SMSF), University of Wisconsin-Madison, 600 Highland Ave, Madison, WI, 53792, United States

* Correspondence: tim.bugni@wisc.edu; Tel.: +1-608-263-2519; Fax: +1-608-262-5345.

‡ J.-X.Y and Q.W. contributed equally to this work.

# Table of contents


**Content** **Page/s**

**Biosynthetic Study of Bacillimidazoles**
Isotopic Enrichment Studies………………………………………………………………………………… **S6**
Determination of Bacillimidazoles as Genuine Natural Products………………………………………..………… **S6–7**

**Table S1.** Antibacterial MIC values (μM) of **1−6**……………………………………………………………………… **S5**
**Table S2**. Summary of bacillimidazole production from six different marine sponge-associated *Bacillus* isolates…… **S5**

**Figure S1.** Analytical HPLC analysis and $^{1}$H NMR analysis of active subfractions…………………………………… **S7**
**Figure S2.** LC-MS analysis (EIC *m/z* 383) of **3**.. …………………………………………………………………… **S8**
**Figure S3.** LC-MS analysis (EIC *m/z* 397) of **4**………………………………………………………………….. **S8**
**Figure S4.** LC-MS analysis of chemical reaction of tryptamine (10.0 mg/mL) and 2,3 butanedione (0.5 equivalent) in MeOH solution without HP-20 resin for 2 h…………………...…… **S8**
**Figure S5.** LC-MS analysis of chemical reaction of tryptamine (10.0 mg/mL) and 2,3 butanedione (0.5 equivalent) in MeOH solution with HP-20 resin for 2 h…………………...……… **S9**
**Figure S6.** LC-MS analysis of chemical reaction of tryptamine (10.0 mg/mL) and 2,3 butanedione (0.5 equivalent) in $CHCl_3$:MeOH= 1:1 solution for 24 h……………………....……… **S10**
**Figure S7.** $^{1}$H NMR Spectrum of Bacillimidazole A (**1**, 600 MHz, $CD_3OD$) …………………………………………… **S11**
**Figure S8.** $^{13}$C NMR Spectrum of Bacillimidazole A (**1**, 125 MHz, $CD_3OD$) ……………………………………….. **S12**
**Figure S9.** gCOSY Spectrum of Bacillimidazole A (**1**, 600 MHz, $CD_3OD$) …………………………………………….. **S13**
**Figure S10.** gHSQC Spectrum of Bacillimidazole A (**1**, 600 MHz, $CD_3OD$) ……………………...……………… **S14**
**Figure S11.** gHMBC Spectrum of Bacillimidazole A (**1**, 600 MHz, $CD_3OD$) ……………………..………………….. **S15**
**Figure S12.** $^{1}$H NMR Spectrum of Bacillimidazole B (**2**, 600 MHz, $CD_3OD$) ……………………..………………… **S16**
**Figure S13.** $^{13}$C NMR Spectrum of Bacillimidazole B (**2**, 125 MHz, $CD_3OD$) ………………………………………….. **S17**
**Figure S14.** gCOSY Spectrum of Bacillimidazole B (**2**, 600 MHz, $CD_3OD$) …………………………………………… **S18**
**Figure S15.** gHSQC Spectrum of Bacillimidazole B (**2**, 600 MHz, $CD_3OD$) …………………………………………… **S19**
**Figure S16.** gHMBC Spectrum of Bacillimidazole B (**2**, 600 MHz, $CD_3OD$) ……………………………………………**S20**

**Figure S17.** $^{1}H$ NMR Spectrum of Bacillimidazole C (**3**, 600 MHz, $CD_3OD$) ........ **S21**
**Figure S18.** $^{13}C$ NMR Spectrum of Bacillimidazole C (**3**, 125 MHz, $CD_3OD$) ........ **S22**
**Figure S19.** gCOSY Spectrum of Bacillimidazole C (**3**, 600 MHz, $CD_3OD$) ........ **S23**
**Figure S20.** gHSQC Spectrum of Bacillimidazole C (**3**, 600 MHz, $CD_3OD$) ........ **S24**
**Figure S21.** gHMBC Spectrum of Bacillimidazole C (**3**, 600 MHz, $CD_3OD$) ........ **S25**
**Figure S22.** $^{1}H$ NMR Spectrum of Bacillimidazole D (**4**, 500 MHz, $CD_3OD$) ........ **S26**
**Figure S23.** $^{13}C$ NMR Spectrum of Bacillimidazole D (**4**, 125 MHz, $CD_3OD$) ........ **S27**
**Figure S24.** gCOSY Spectrum of Bacillimidazole D (**4**, 500 MHz, $CD_3OD$) ........ **S28**
**Figure S25.** gHSQC Spectrum of Bacillimidazole D (**4**, 500 MHz, $CD_3OD$) ........ **S29**
**Figure S26.** gHMBC Spectrum of Bacillimidazole D (**4**, 500 MHz, $CD_3OD$) ........ **S30**
**Figure S27.** $^{1}H$ NMR Spectrum of Bacillimidazole E (**5**, 600 MHz, $CD_3OD$) ........ **S31**
**Figure S28.** $^{13}C$ NMR Spectrum of Bacillimidazole E (**5**, 125 MHz, $CD_3OD$) ........ **S32**
**Figure S29.** gCOSY Spectrum of Bacillimidazole E (**5**, 600 MHz, $CD_3OD$) ........ **S33**
**Figure S30.** gHSQC Spectrum of Bacillimidazole E (**5**, 600 MHz, $CD_3OD$) ........ **S34**
**Figure S31.** gHMBC Spectrum of Bacillimidazole E (**5**, 600 MHz, $CD_3OD$) ........ **S35**
**Figure S32.** $^{1}H$ NMR Spectrum of Bacillimidazole F (**6**, 500 MHz, $CD_3OD$) ........ **S36**
**Figure S33.** $^{13}C$ NMR Spectrum of Bacillimidazole F (**6**, 125 MHz, $CD_3OD$) ........ **S37**
**Figure S34.** gCOSY Spectrum of Bacillimidazole F (**6**, 500 MHz, $CD_3OD$) ........ **S38**
**Figure S35.** gHSQC Spectrum of Bacillimidazole F (**6**, 500 MHz, $CD_3OD$) ........ **S39**
**Figure S36.** gHMBC Spectrum of Bacillimidazole F (**6**, 500 MHz, $CD_3OD$) ........ **S40**
**Figure S37.** Positive Ion HRESIMS of Bacillimidazole A (**1**) ........ **S41**
**Figure S38.** Positive Ion HRESIMS of Bacillimidazole B (**2**) ........ **S42**
**Figure S39.** Positive Ion HRESIMS of Bacillimidazole C (**3**) ........ **S43**
**Figure S40.** Positive Ion HRESIMS of Bacillimidazole D (**4**) ........ **S44**
**Figure S41.** Positive Ion HRESIMS of Bacillimidazole E (**5**) ........ **S45**
**Figure S42.** Positive Ion HRESIMS of Bacillimidazole F (**6**) ........ **S46**
**Figure S43.** $^{1}H$ NMR Spectrum of $^{13}C$ Labeled Bacillimidazole C (**3**, 500 MHz, $CD_3OD$) ........ **S47**
**Figure S44.** $^{13}C$ NMR Spectrum of $^{13}C$ Labeled Bacillimidazole C (**3**, 125 MHz, $CD_3OD$) ........ **S48**
**Figure S45.** $^{1}H$ NMR Spectrum of $^{13}C$ Labeled Bacillimidazole E (**5**, 500 MHz, $CD_3OD$) ........ **S49**
**Figure S46.** $^{13}C$ NMR Spectrum of $^{13}C$ Labeled Bacillimidazole E (**5**, 125 MHz, $CD_3OD$) ........ **S50**
**Figure S47.** Positive Ion HRESIMS of $^{13}C$ Enriched Bacillimidazole C (**3**) ........ **S51**

**Figure S48.** Positive Ion HRESIMS of $^{13}C$ Enriched Bacillimidazole E (**5**)…………………………………………… **S52**
**Figure S49.** LC-MS analysis of strain *Bacillus* sp. WMMC325………………………………………………………… **S53**
**Figure S50.** LC-MS analysis of strain *Bacillus* sp. WMMC331………………………………………………………… **S54**
**Figure S51.** LC-MS analysis of strain *Bacillus* sp. WMMC1349………………………………………………………… **S55**
**Figure S52.** LC-MS analysis of strain *Bacillus* sp. WMMC1350………………………………………………………… **S56**
**Figure S53.** LC-MS analysis of strain *Bacillus* sp. WMMC1351………………………………………………………… **S57**
**Figure S54.** LC-MS analysis of strain *Bacillus* sp. WMMC1352………………………………………………………… **S58**
**Figure S55.** Culture broth of six different marine *Bacillus* strains………………………………………………….. **S59**
**Figure S56.** Pictures for sponge hosts of six different marine *Bacillus* strains…………………………………………… **S60**

**References**…………………………………………………………………………………………………………… **S61**

**Table S1.** Antibacterial MIC values (μM) of **1**−**6**.

| Compounds | MRSA | *B. subtilis* | *E. coli* |
|---|---|---|---|
| **1** | –[b] | – | – |
| **2** | – | – | – |
| **3** | – | – | – |
| **4** | – | – | – |
| **5** | – | – | – |
| **6** | 38.3 | – | – |
| Control[a] | 0.168 | 0.168 | 7.74 |

[a]Vancomycin hydrochloride was used as positive controls against MRSA and *B. subtilis*. Gentamicin sulfate was used as a positive control against *E. coli*. [b]MIC values over 50 μM.

**Table S2.** Summary of bacillimidazole production from six different marine sponge-associated *Bacillus* isolates (based partly on data of **Figures S49–S54**).

| | WMMC325 | WMMC331 | WMMC1349 | WMMC1350 | WMMC1351 | WMMC1352 |
|---|---|---|---|---|---|---|
| Sponge host | *Ecteinascidia turbinata* | *Ecteinascidia turbinata* | *Cinachyrella apion* | *Microcosmus exasperatus* | *Geodia gibberosa* | *Chalinula molitba* |
| Sponge collection location | Florida Keys, 24°39′39.3″ N, 81°26′26.8″ W | Florida Keys, 24°39′39.3″ N, 81°26′26.8″ W | Florida Keys, 24°39′38.1″ N, 81°25′25.0″ W | Florida Keys, 24°39′39.8″ N, 81°26′27.8″ W | Florida Keys, 24°39′38.1″ N, 81°25′25.0″ W | Florida Keys, 24°39′39.8″ N, 81°26′27.8″ W |
| Sponge collection year | 2014 | 2014 | 2015 | 2015 | 2015 | 2015 |
| Bacillimidazole production | – | – | + | +[a] | – | – |
| Broth color | white | white | brown | brown | white | white |

[a]Bacillimidazole D (*m/z*= 397) was not detected in strain WMMC1350.

# 1. Biosynthetic Study of Bacillimidazoles

## 1.1 Isotopic Enrichment Studies

Isotopic labeling of the bacillimidazoles using $^{13}C$ enriched culture media was performed to verify bacillimidazoles were produced by the producing organism. By substituting two carbon sources, soluble starch and D-glucose, with $^{13}C_6$-D-glucose, only four carbons on the imidazolium ring were labeled with high level of $^{13}C$ incorporation for bacillimidazoles C (**3**) and E (**5**) (**Figures S43−S48**), which substantiated that the bacillimidazoles are biologically synthesized by *Bacillus* sp. WMMC1349. Only low levels of $^{13}C$ incorporation was observed for the rest of the molecule, suggesting the biological source of those carbons are different from glucose.

## 1.2 Determination of bacillimidazoles as genuine natural products

Biosynthetic and structural analysis of these bacillimidazoles revealed the possibility that imidazole formation might result from the chemical workup procedures during extraction and isolation. To address this question, systematic chemical reactions with two building blocks, tryptamine and 2,3 butanedione, which were proposed to be the precursors of bacillimidazoles C and D (**3** and **4**) were conducted. To exactly mimic the extraction and isolation procedure, high concentrations (10.0 mg/mL) of tryptamine and 2,3 butanedione (0.5 equivalent) were exposed in MeOH with/without HP-20 resin for 2 h (extraction procedure took about 2 h before LC-MS analysis and isolation) and $CHCl_3$: MeOH= 1:1 solution for 24 h (isolation procedure and concentration of samples usually took less than 12 h), respectively. The resulting solutions were then analyzed by LC-MS. The results showed that no corresponding bacillimidazoles could be

detected (**Figures S4−S6**). Besides, no acidic conditions were applied during cultivation, extraction and LH-20 separation. TFA was only applied for the further purification of bacillimidazoles by Prep-HPLC after successful separation of bacillimidazoles from the building blocks. In conclusion, bacillimidazoles were shown to be natural products produced by marine *Bacillus* sp. WMMC1349 during cultivation; serendipitous bacillimidazole formation resulting from (or during) workup was deemed to be negligible on the basis of these control reactions.

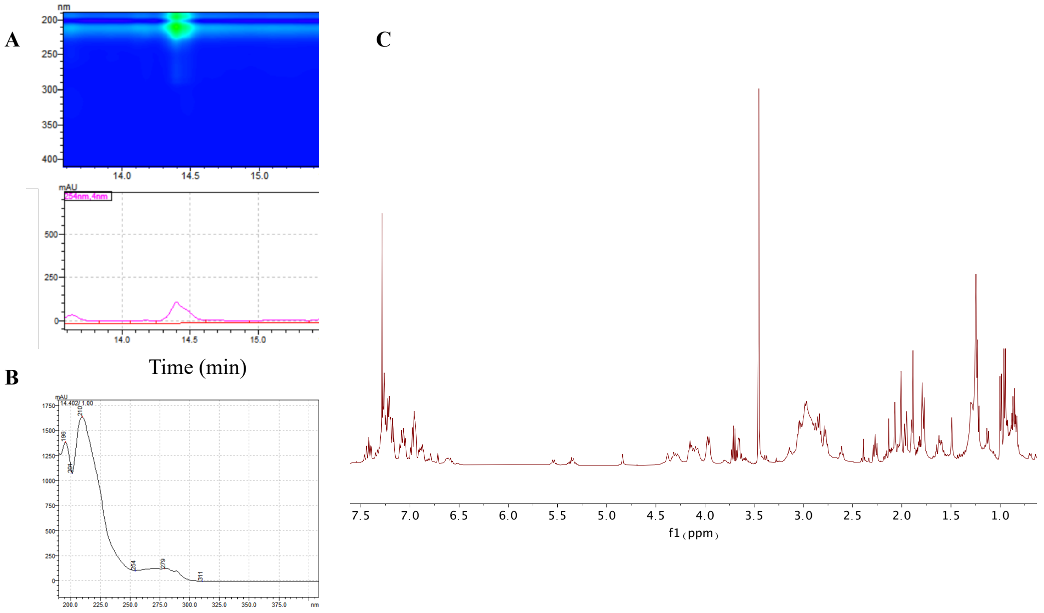


**Figure S1.** Analytical HPLC analysis and $^{1}H$ NMR analysis of active subfractions. A: Analytical HPLC analysis showed a broad chromatographic peak. B: The UV spectrum showed good consistency of the broad chromatographic peak ($t_R$=14.3−14.6 min). C: $^{1}H$ NMR spectrum demonstrated the fraction was a mixture.

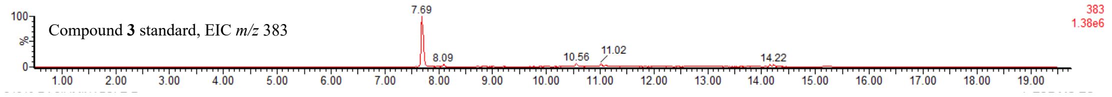


**Figure S2.** LC-MS analysis (EIC *m/z* 383) of **3**.

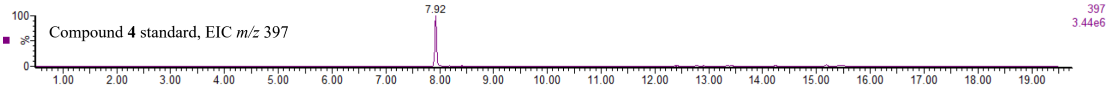


**Figure S3.** LC-MS analysis (EIC *m/z* 397) of **4**.

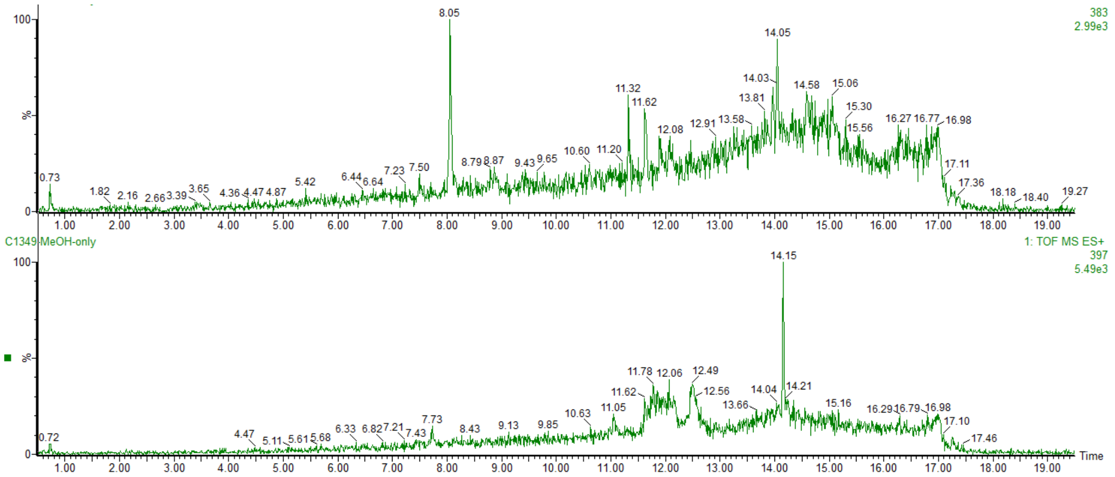


**Figure S4.** LC-MS analysis of chemical reaction of tryptamine (10.0 mg/mL) and 2,3 butanedione (0.5 equivalent) in MeOH solution without HP-20 resin for 2 h. EIC for 383 (compound **3**, up) and 397 (compound **4**, down). EIC for standards see **Figures S2** and **S3**.

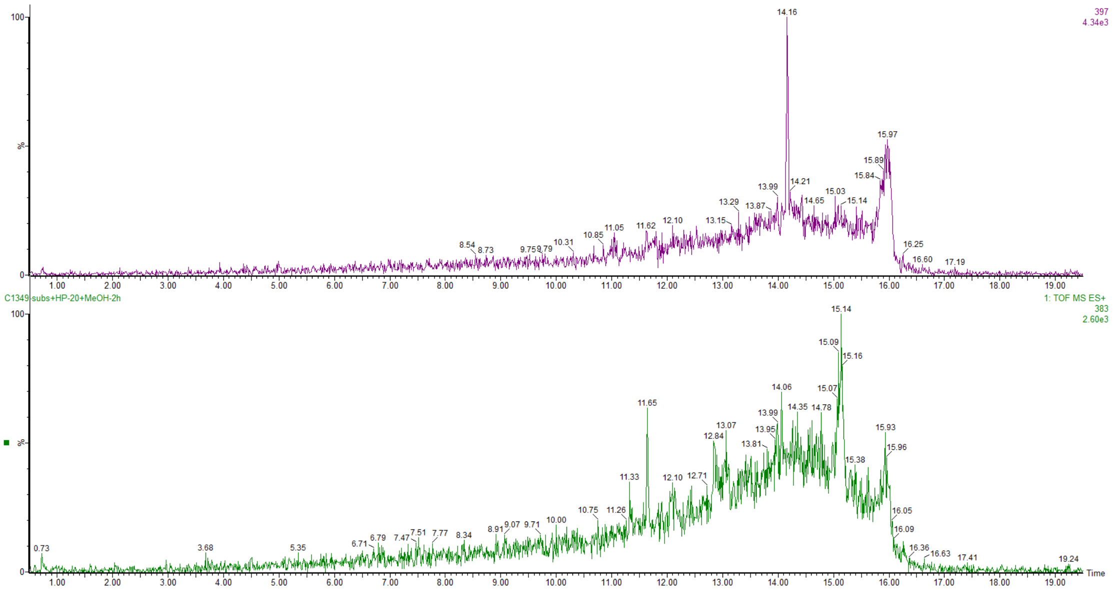


**Figure S5.** LC-MS analysis of chemical reaction of tryptamine (10.0 mg/mL) and 2,3 butanedione (0.5 equivalent) in MeOH solution with HP-20 resin for 2 h. EIC for 383 (compound **3**, down) and 397 (compound **4**, up). EIC for standards see **Figures S2** and **S3**.

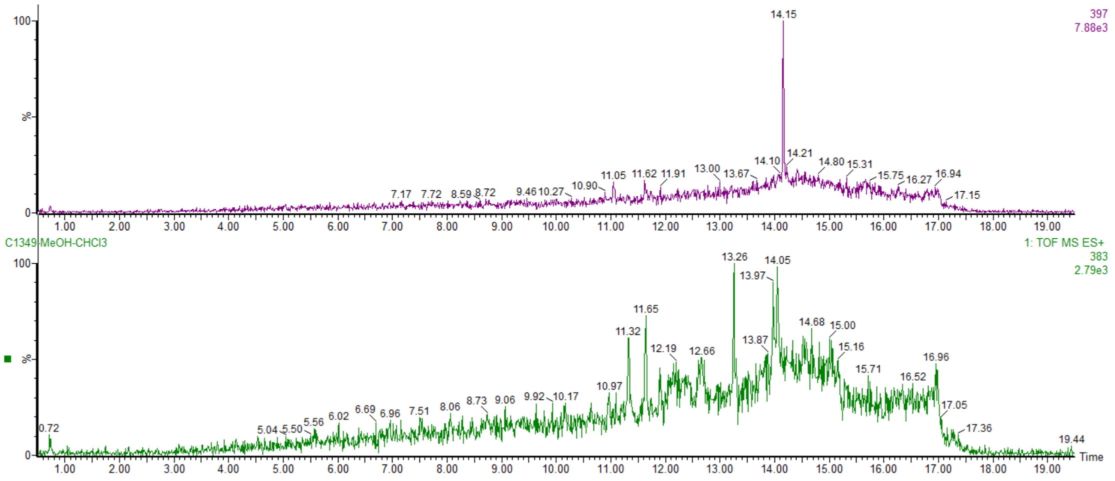


**Figure S6.** LC-MS analysis of chemical reaction of tryptamine (10.0 mg/mL) and 2,3 butanedione (0.5 equivalent) in $CHCl_3$:MeOH= 1:1 solution for 24 h. EIC for 383 (compound **3**, down) and 397 (compound **4**, up). EIC for standards see **Figures S2** and **S3**.

# 3. NMR and MS spectra for Bacillimidazoles

**Figure S7.** $^{1}$H NMR Spectrum of Bacillimidazole A (**1**, 600 MHz, $CD_3OD$)

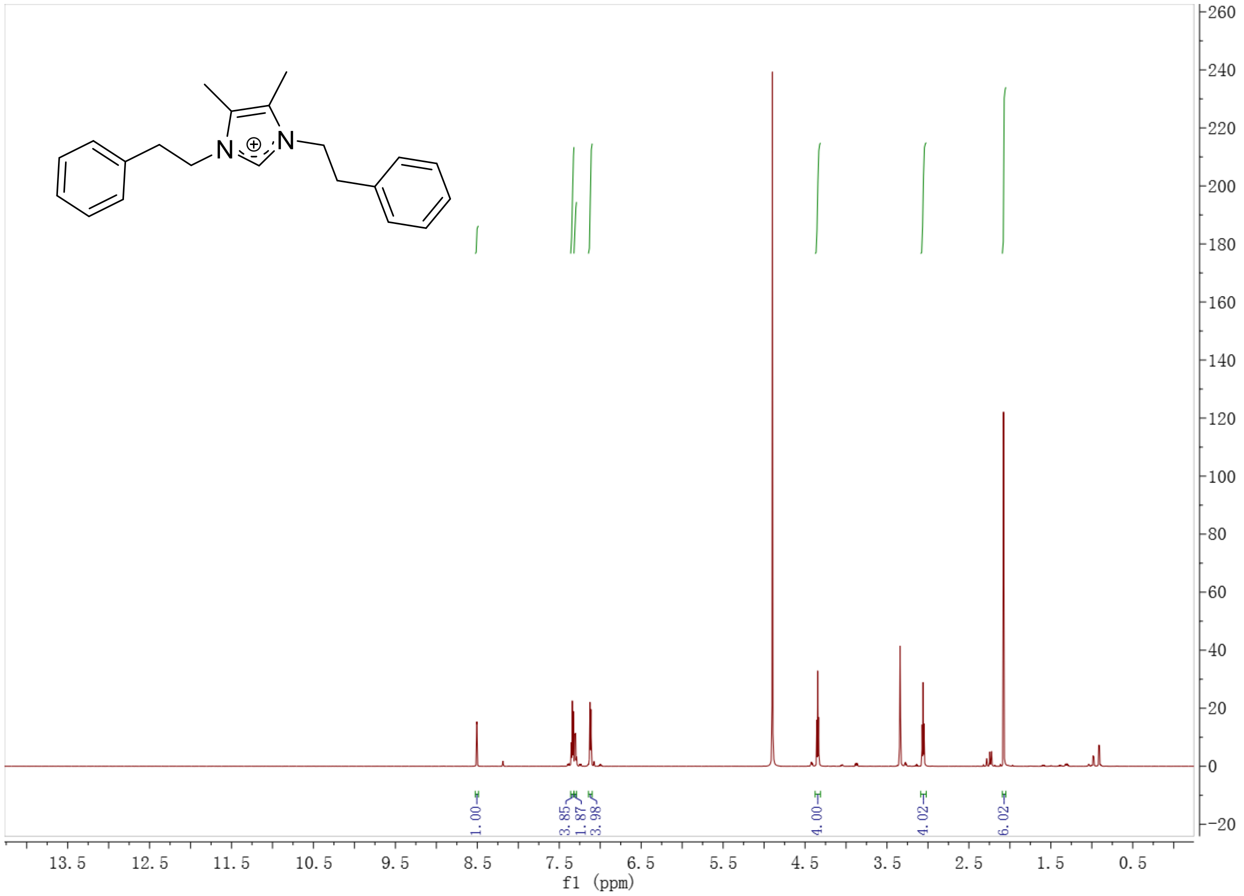

**Figure S8.** $^{13}$C NMR Spectrum of Bacillimidazole A (**1**, 125 MHz, $CD_3OD$)

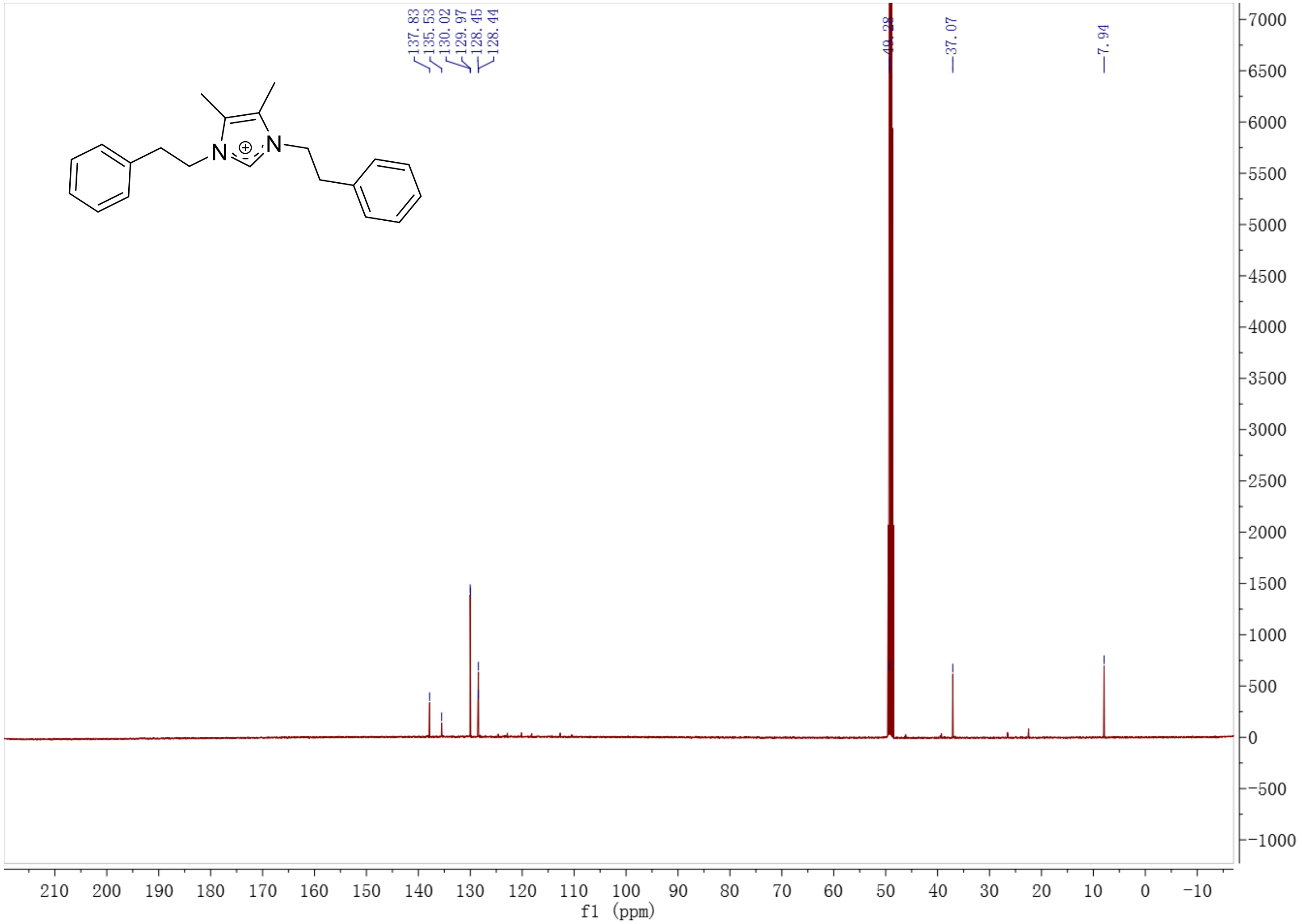

**Figure S9.** gCOSY Spectrum of Bacillimidazole A (**1**, 600 MHz, $CD_3OD$)

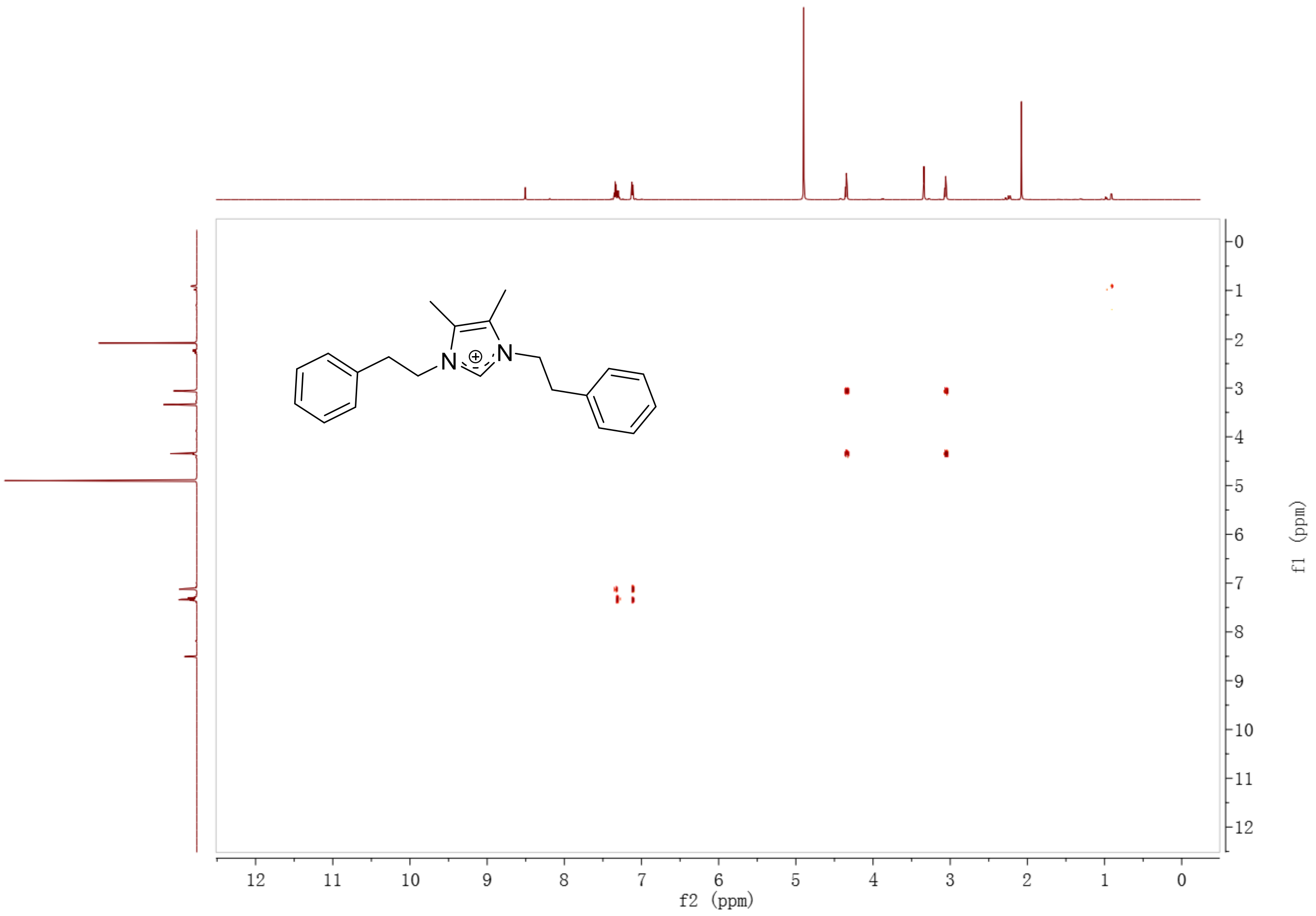

**Figure S10.** gHSQC Spectrum of Bacillimidazole A (**1**, 600 MHz, $CD_3OD$)

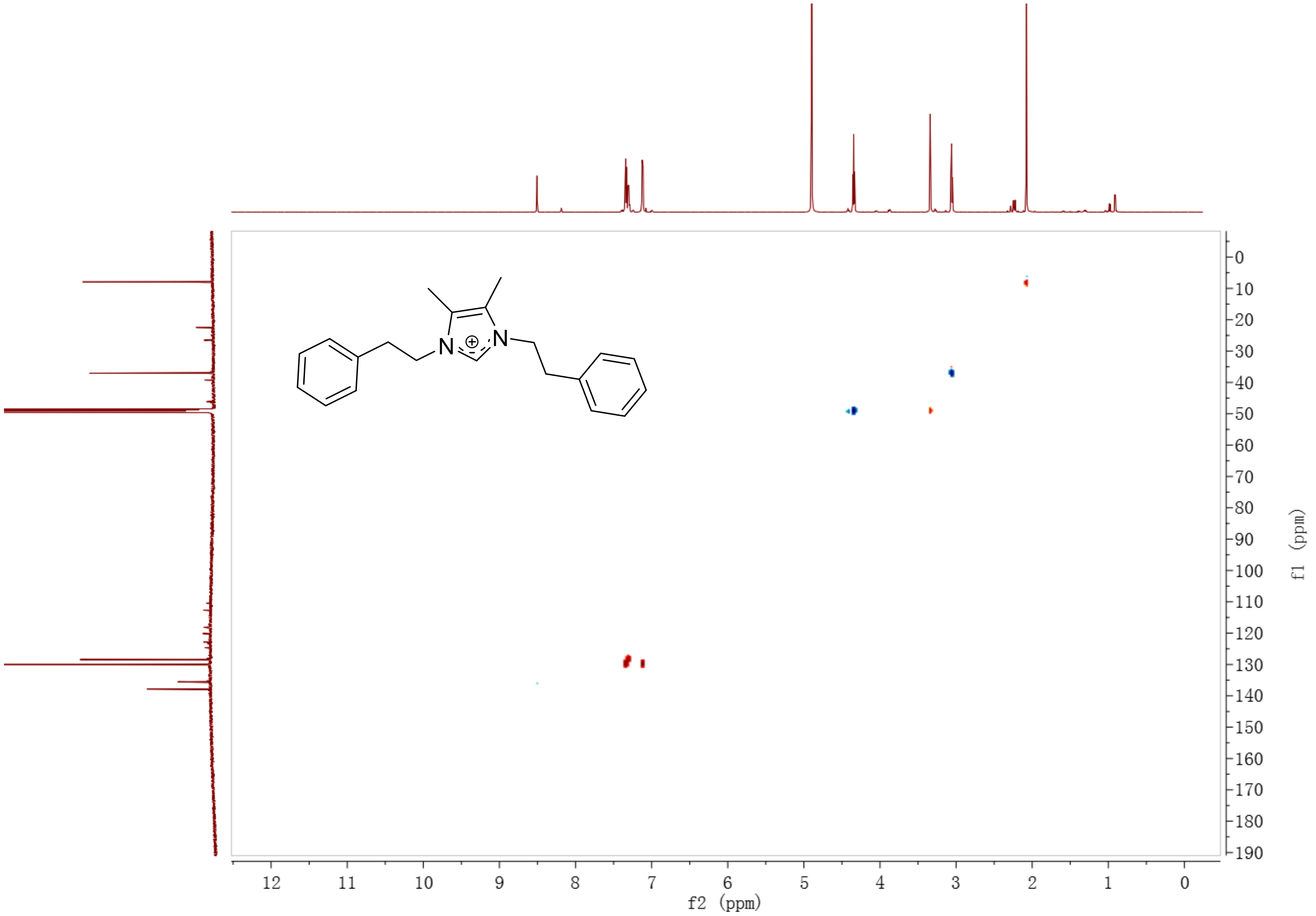

**Figure S11.** gHMBC Spectrum of Bacillimidazole A (**1**, 600 MHz, $CD_3OD$)

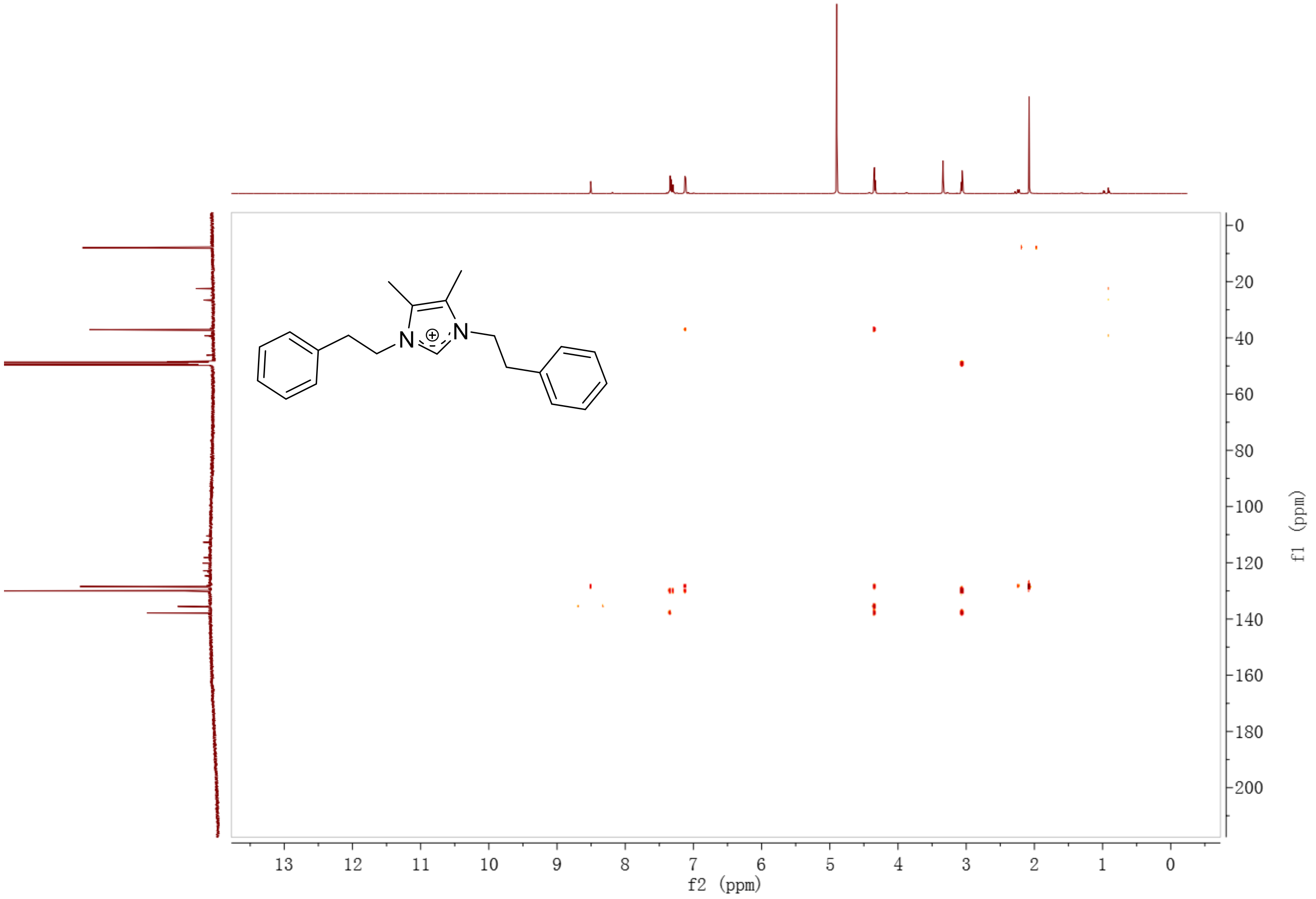

**Figure S12.** $^{1}$H NMR Spectrum of Bacillimidazole B (**2**, 600 MHz, $CD_3OD$)

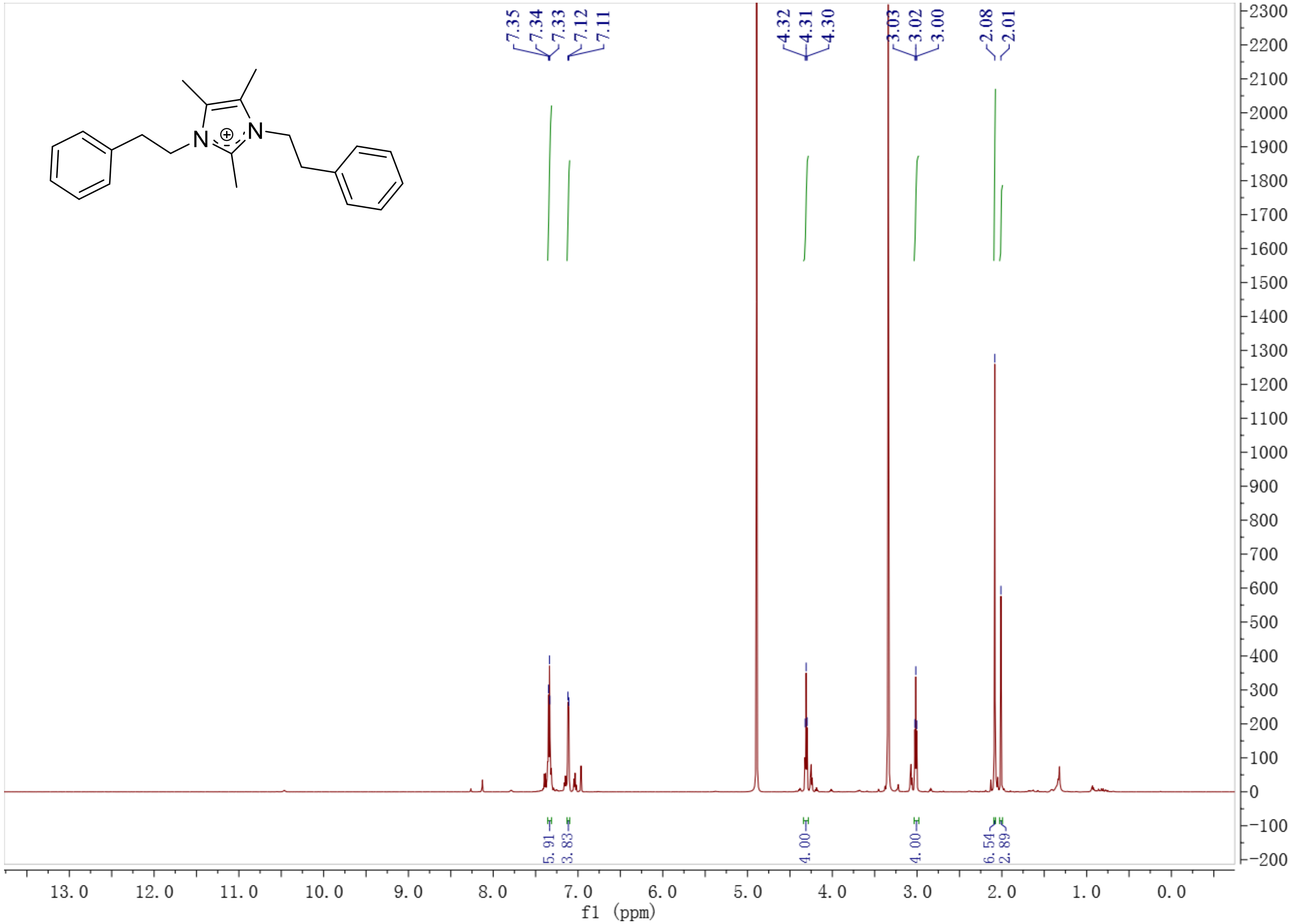

**Figure S13.** $^{13}$C NMR Spectrum of Bacillimidazole B (**2**, 125 MHz, $CD_3OD$)

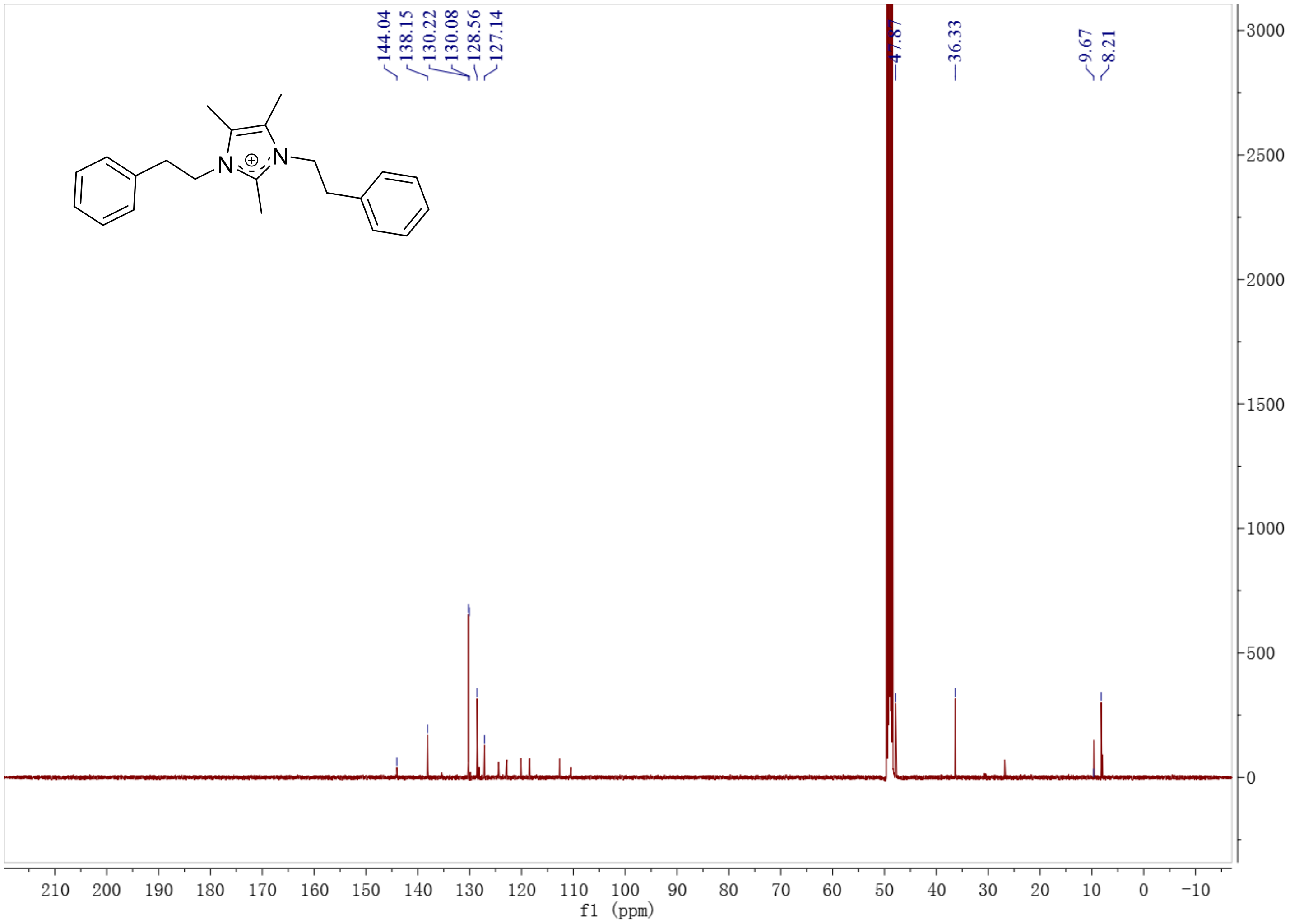

**Figure S14.** gCOSY Spectrum of Bacillimidazole B (**2**, 600 MHz, $CD_3OD$)

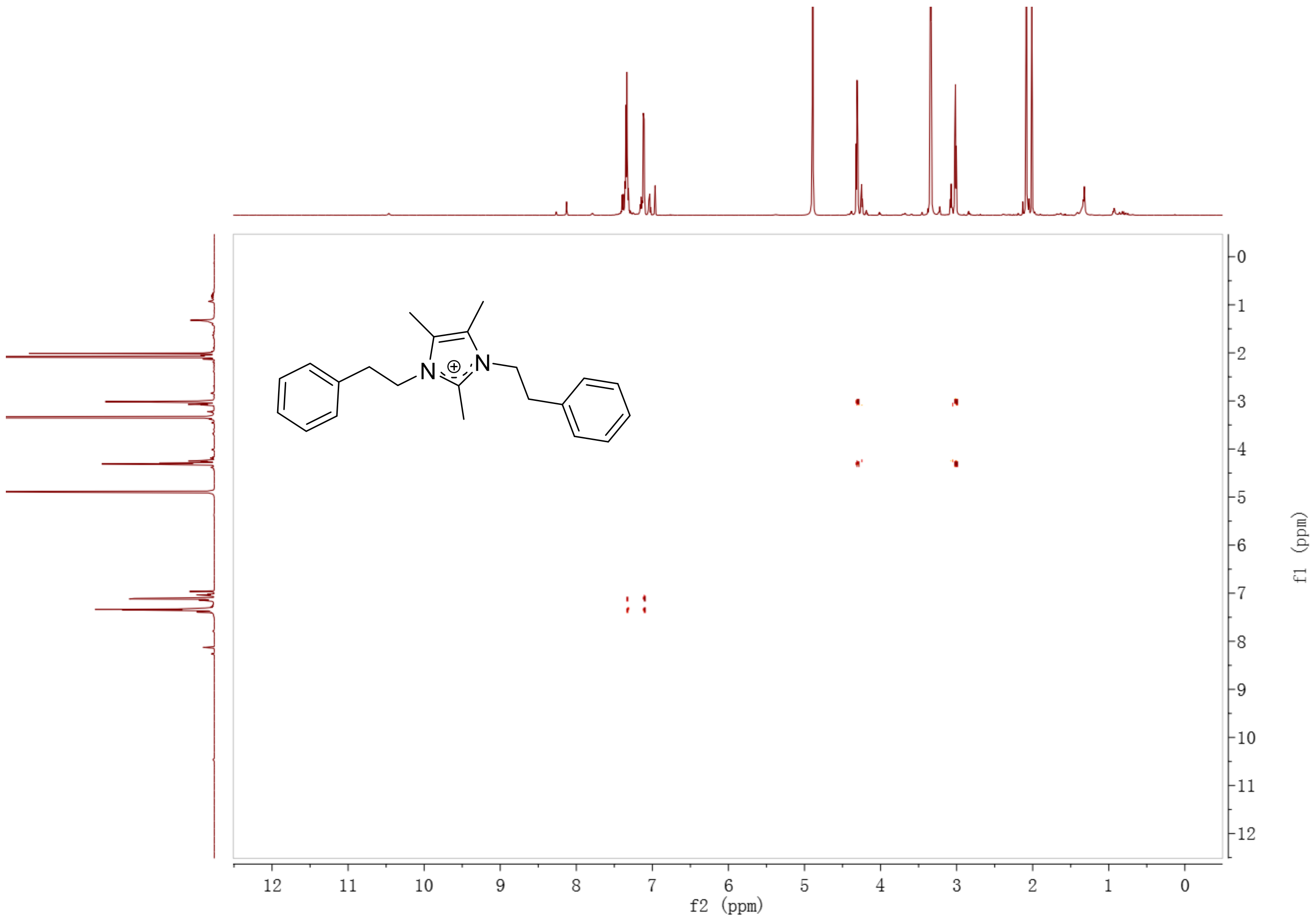

**Figure S15.** gHSQC Spectrum of Bacillimidazole B (**2**, 600 MHz, $CD_3OD$)

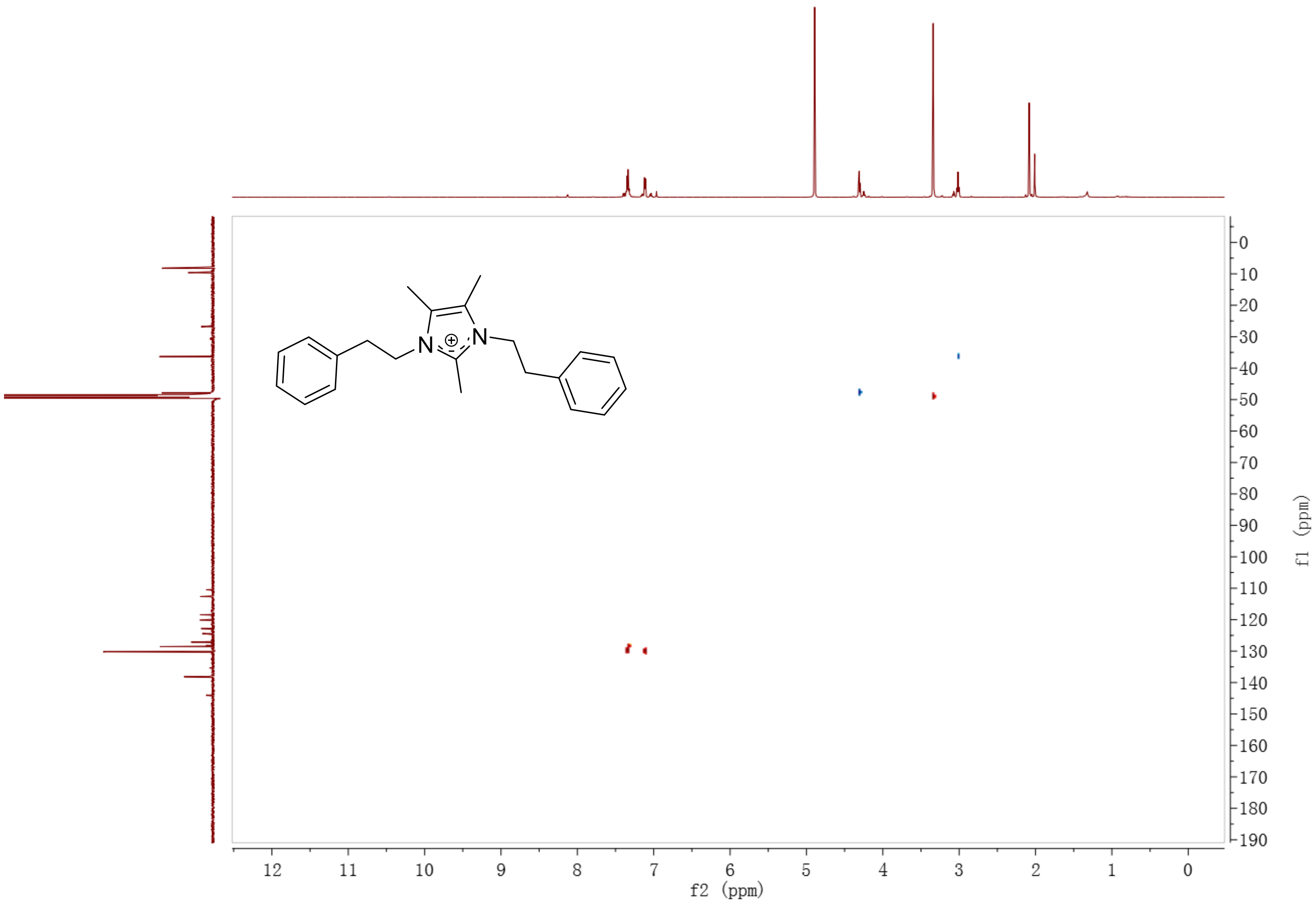

**Figure S16.** gHMBC Spectrum of Bacillimidazole B (**2**, 600 MHz, $CD_3OD$)

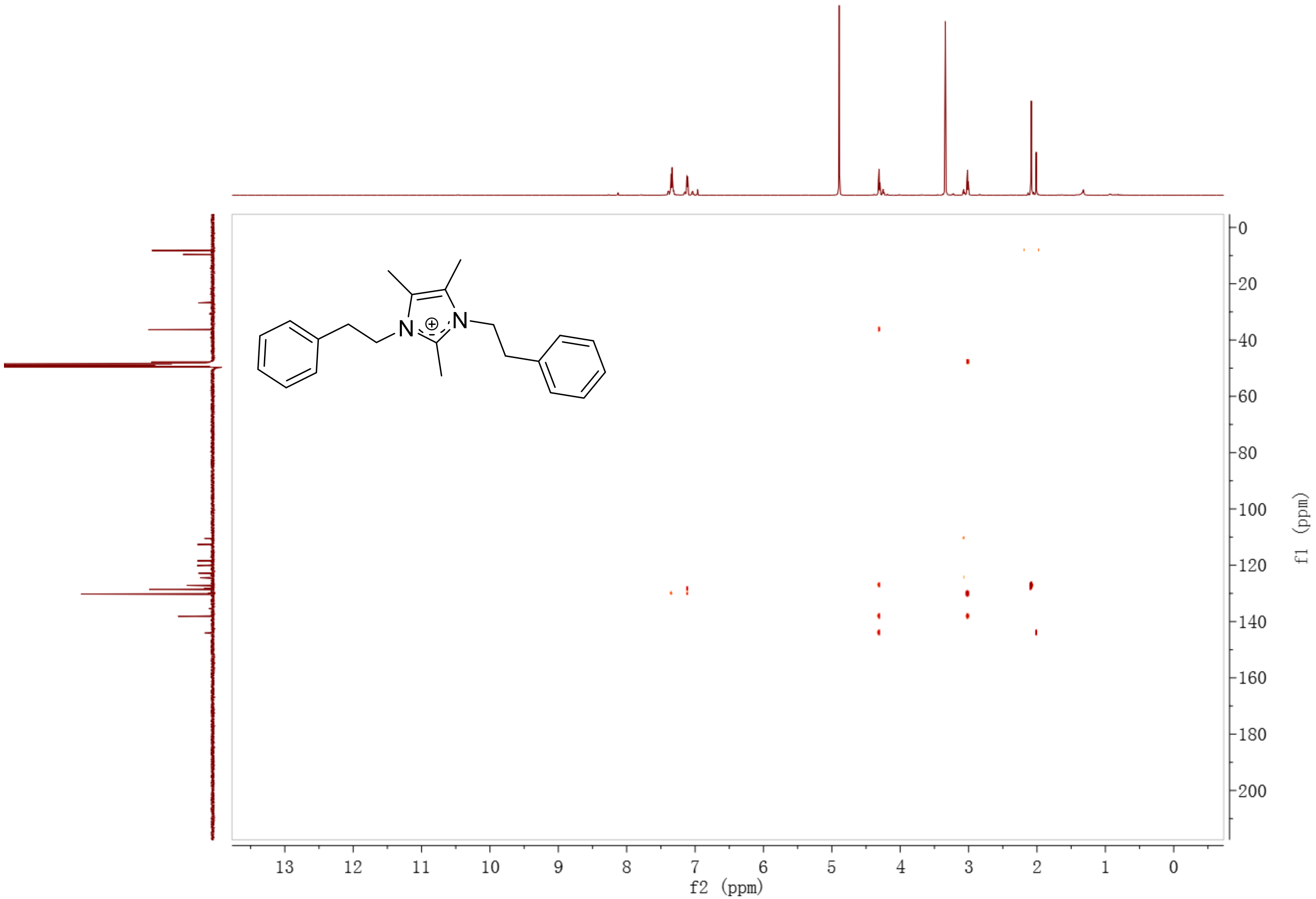

**Figure S17.** $^1$H NMR Spectrum of Bacillimidazole C (**3**, 600 MHz, $CD_3OD$)

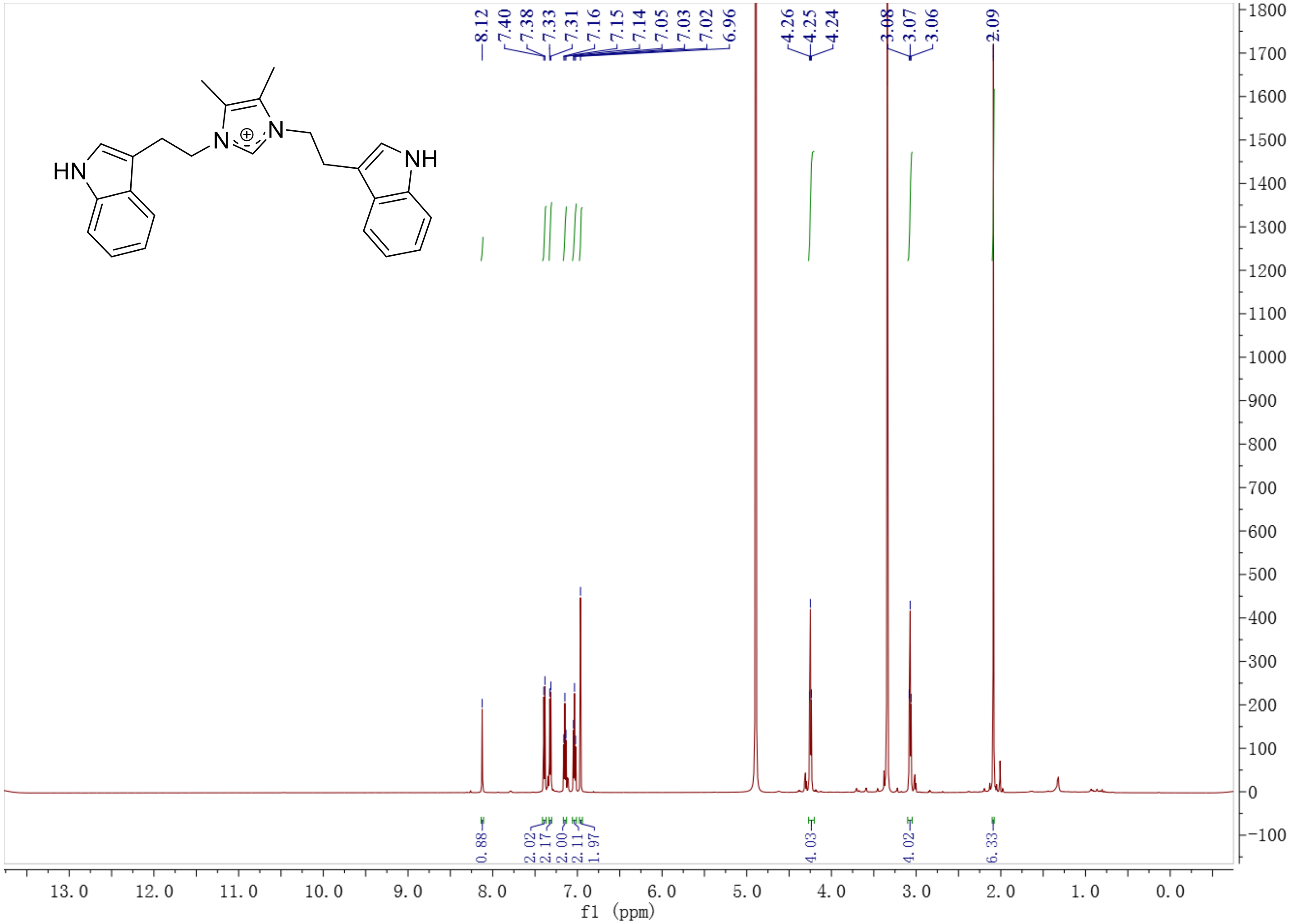

**Figure S18.** $^{13}$C NMR Spectrum of Bacillimidazole C (**3**, 125 MHz, $CD_3OD$)

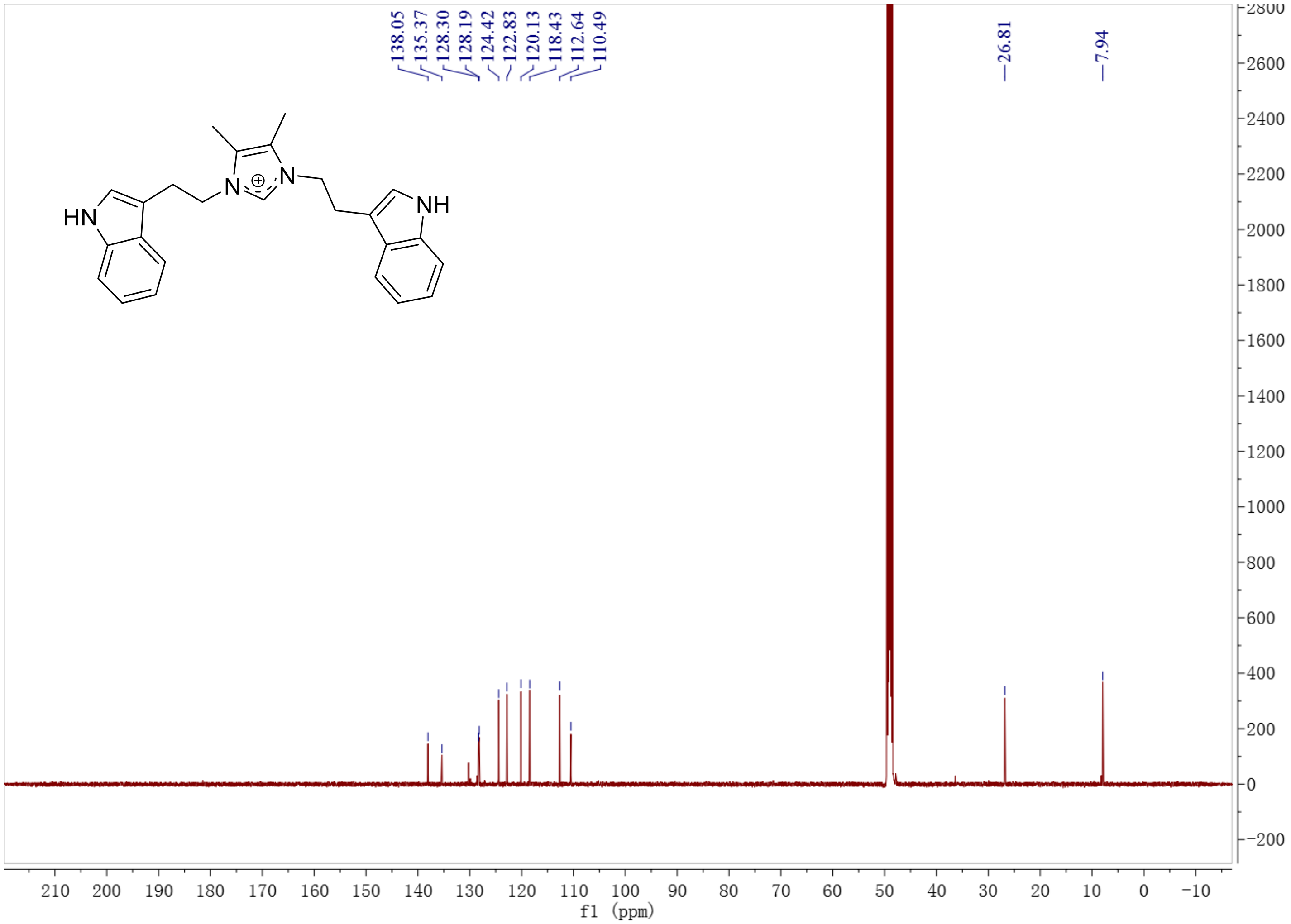

**Figure S19.** gCOSY Spectrum of Bacillimidazole C (**3**, 600 MHz, $CD_3OD$)

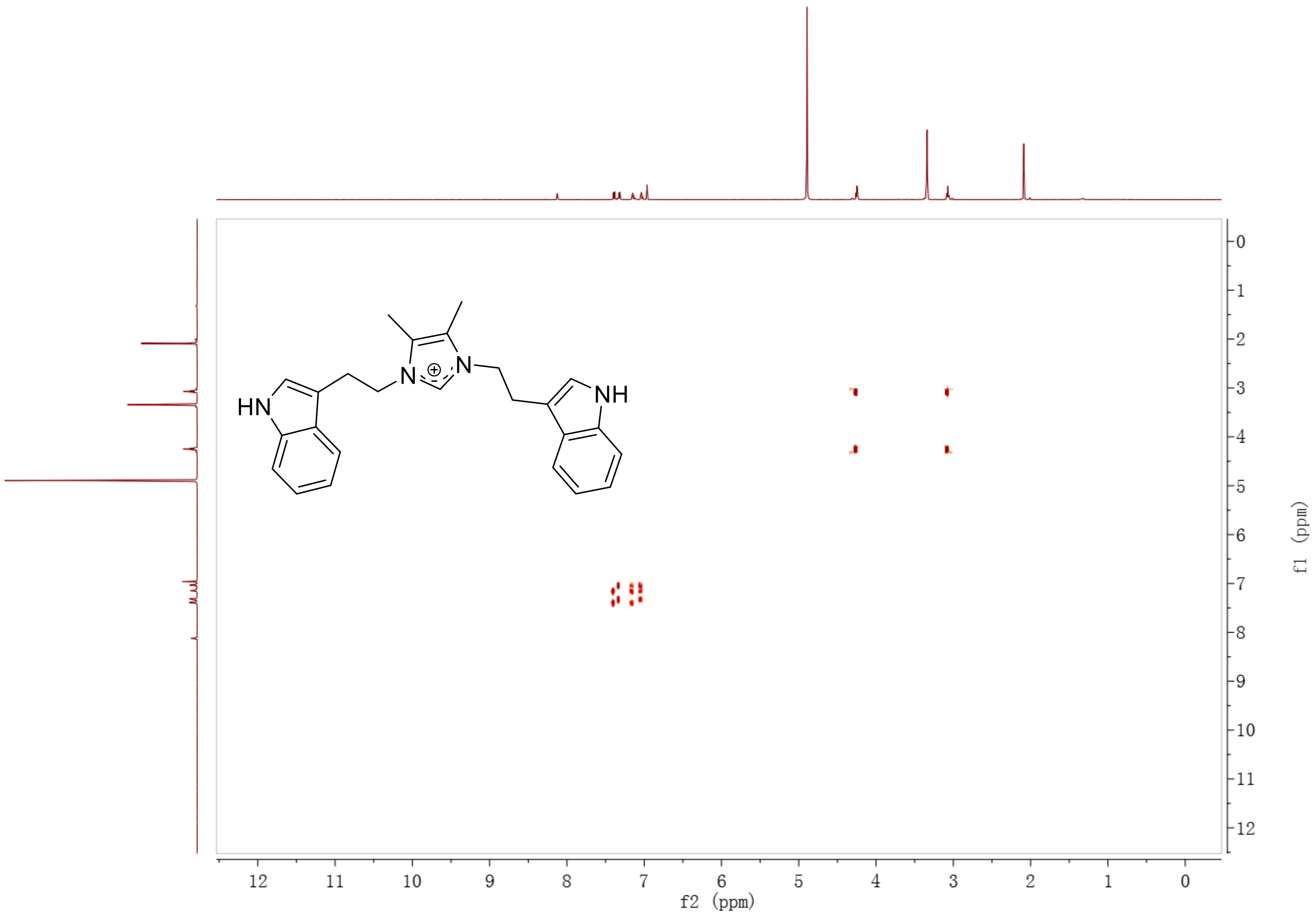

**Figure S20.** gHSQC Spectrum of Bacillimidazole C (**3**, 600 MHz, $CD_3OD$)

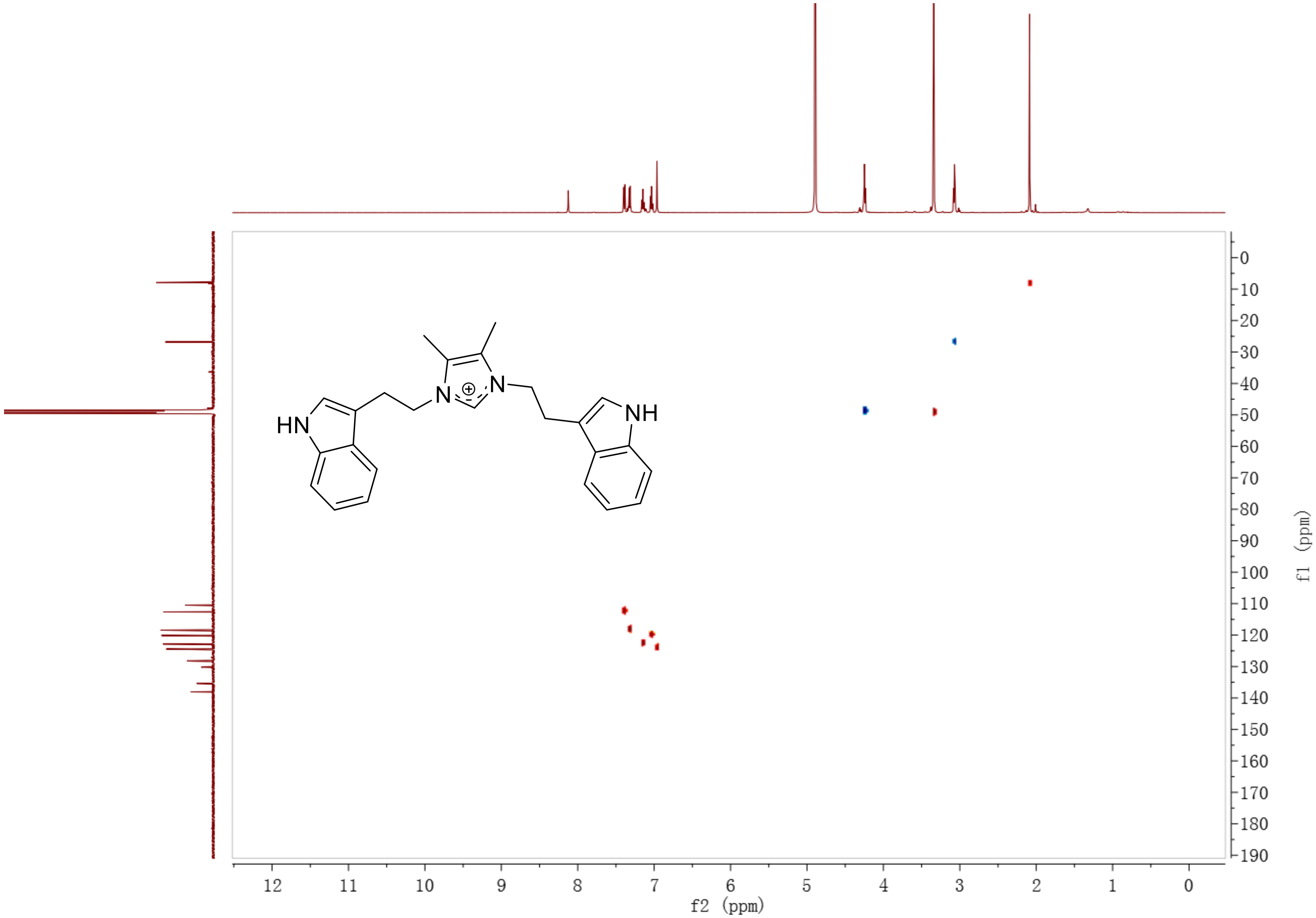

**Figure S21.** gHMBC Spectrum of Bacillimidazole C (**3**, 600 MHz, $CD_3OD$)

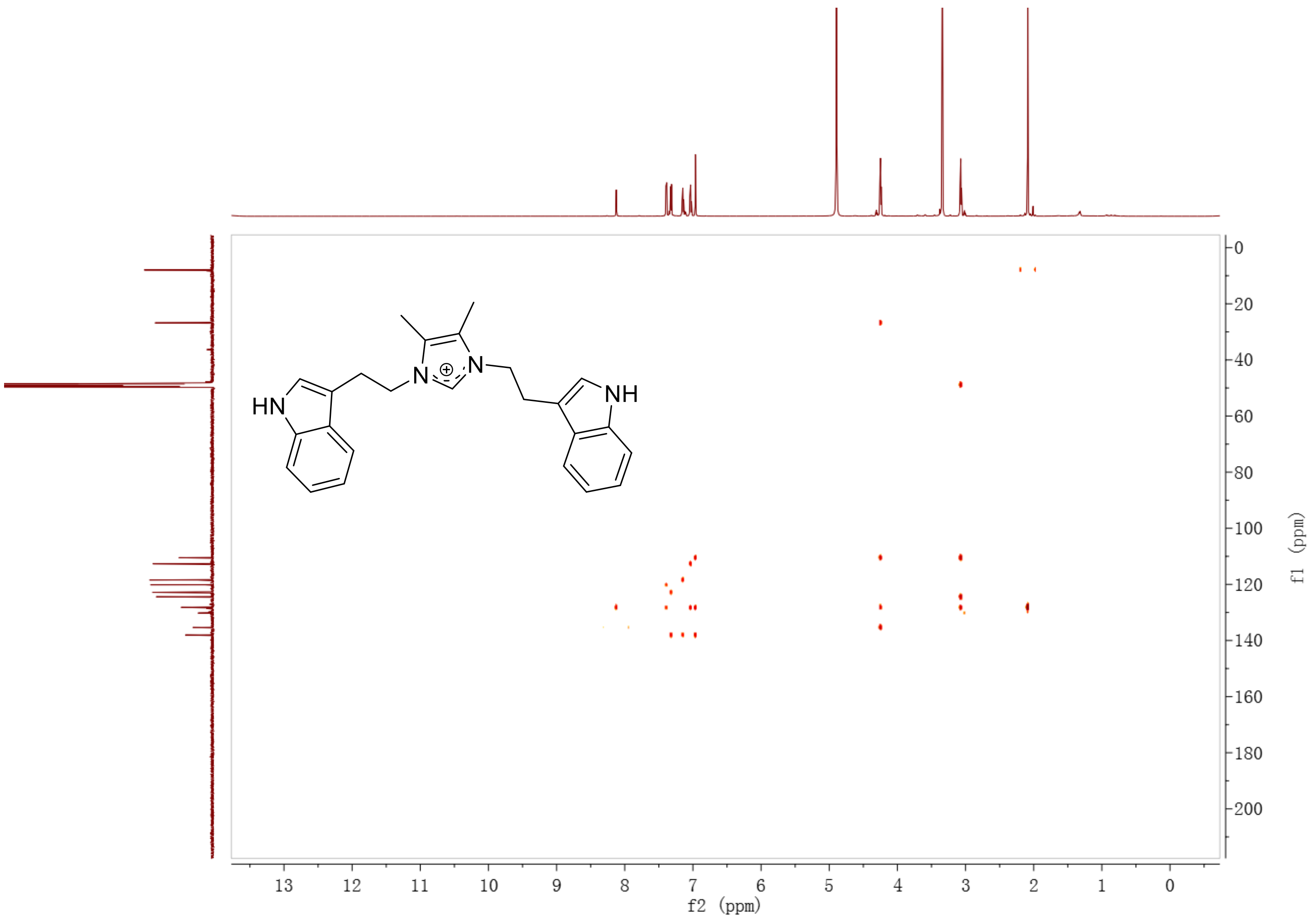

**Figure S22.** $^{1}$H NMR Spectrum of Bacillimidazole D (**4**, 500 MHz, $CD_3OD$)

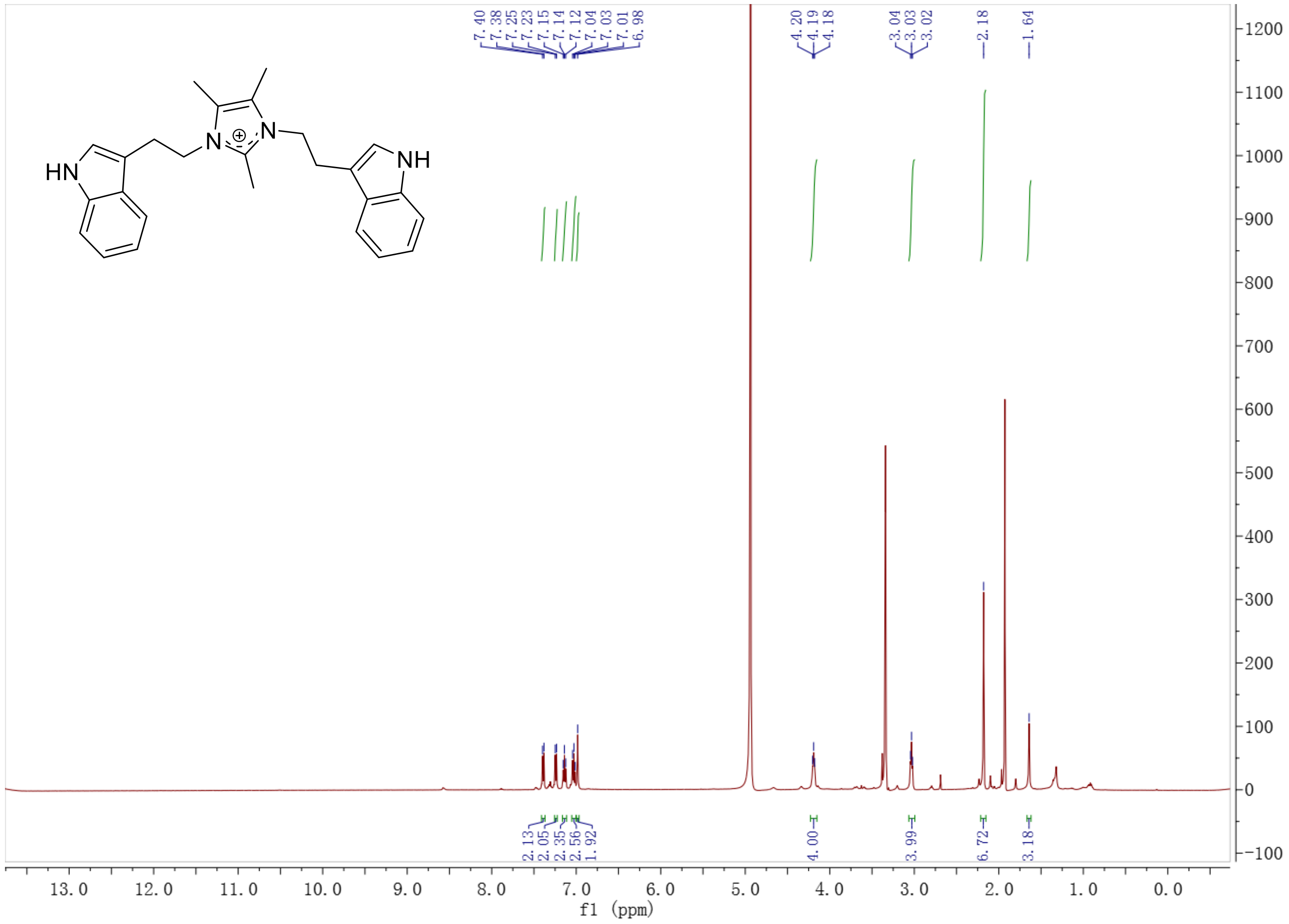

**Figure S23.** $^{13}$C NMR Spectrum of Bacillimidazole D (**4**, 125 MHz, $CD_3OD$)

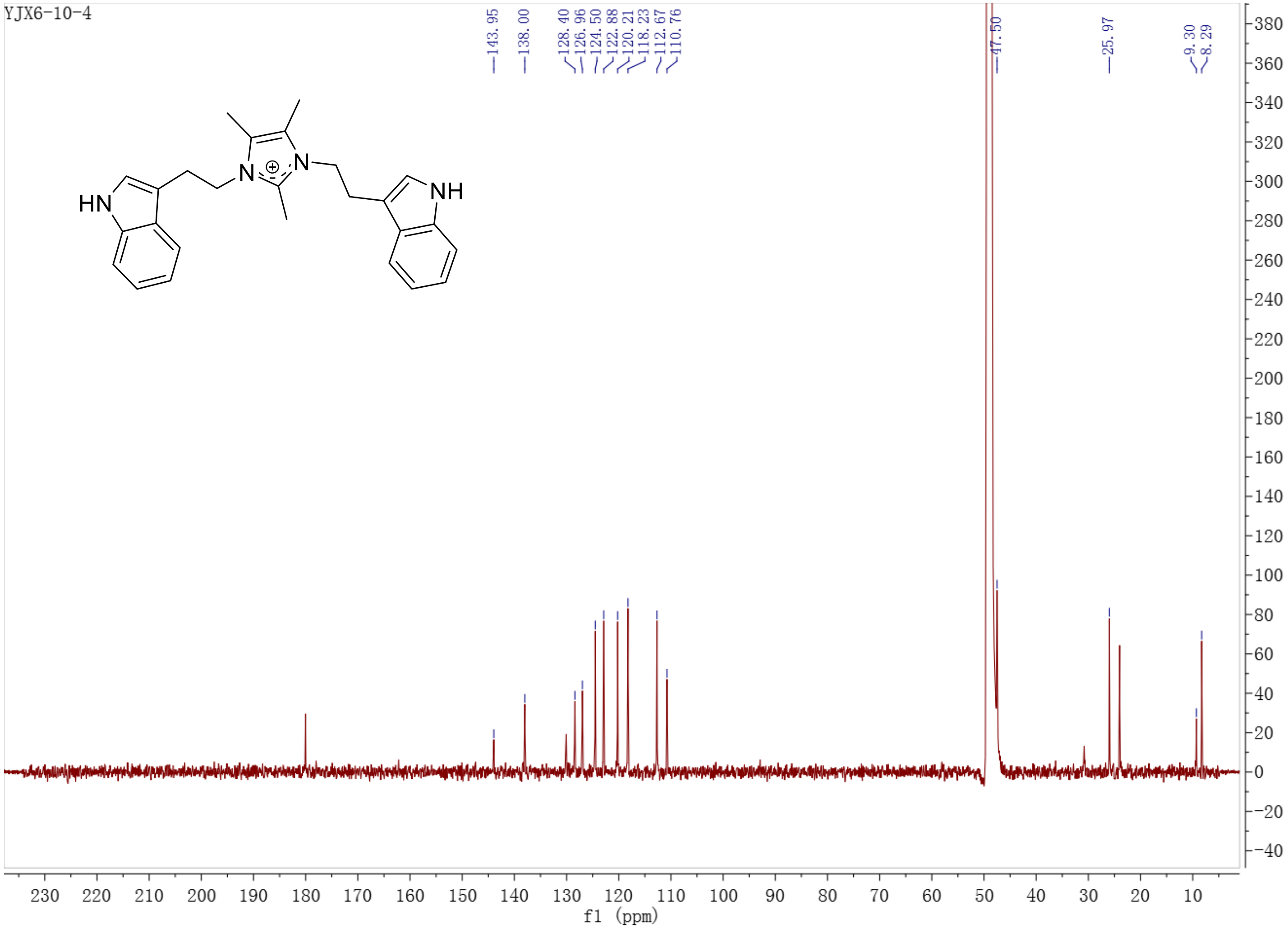

**Figure S24.** gCOSY Spectrum of Bacillimidazole D (**4**, 500 MHz, $CD_3OD$)

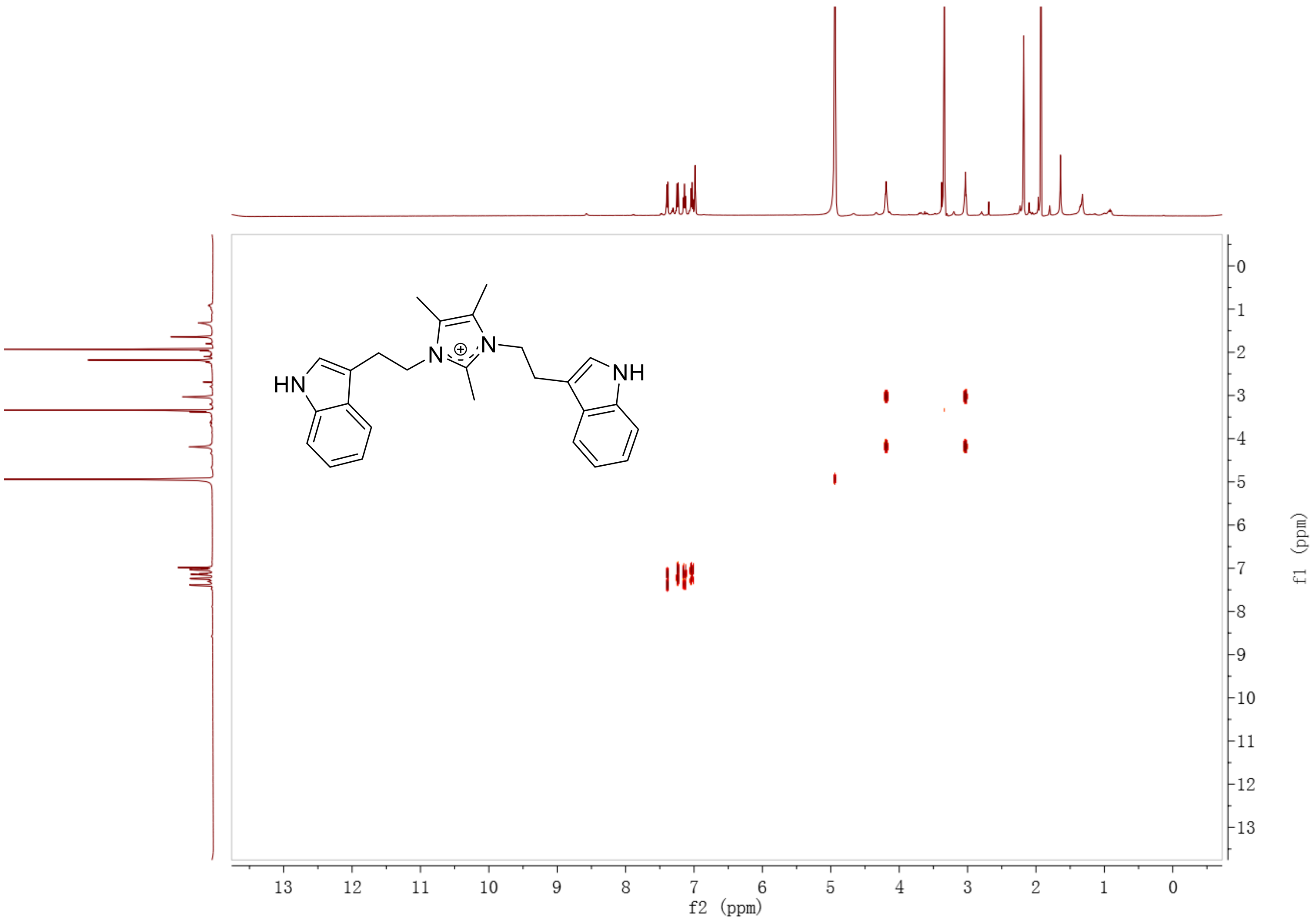

**Figure S25.** gHSQC Spectrum of Bacillimidazole D (**4**, 500 MHz, $CD_3OD$)

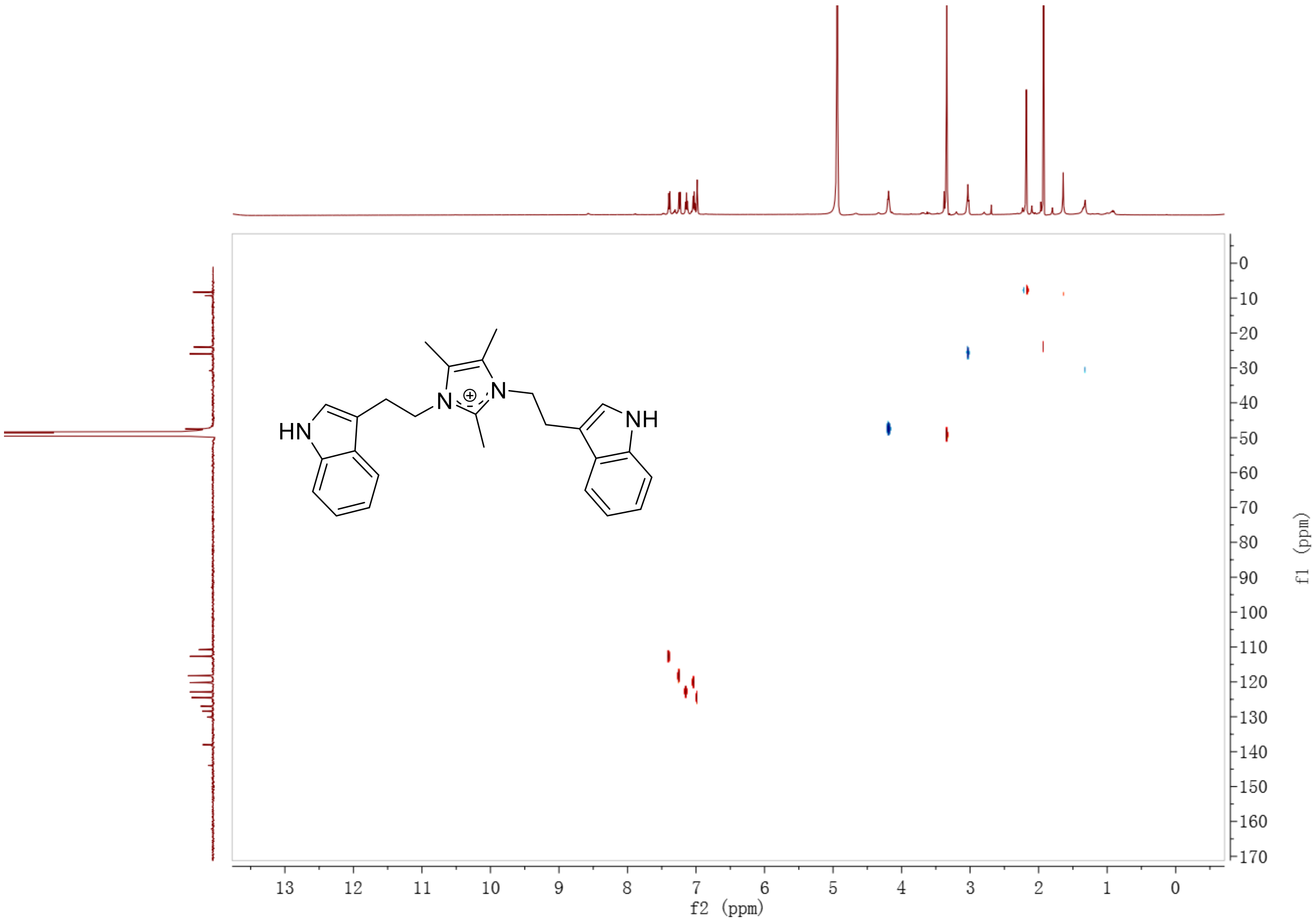

**Figure S26.** gHMBC Spectrum of Bacillimidazole D (**4**, 500 MHz, $CD_3OD$)

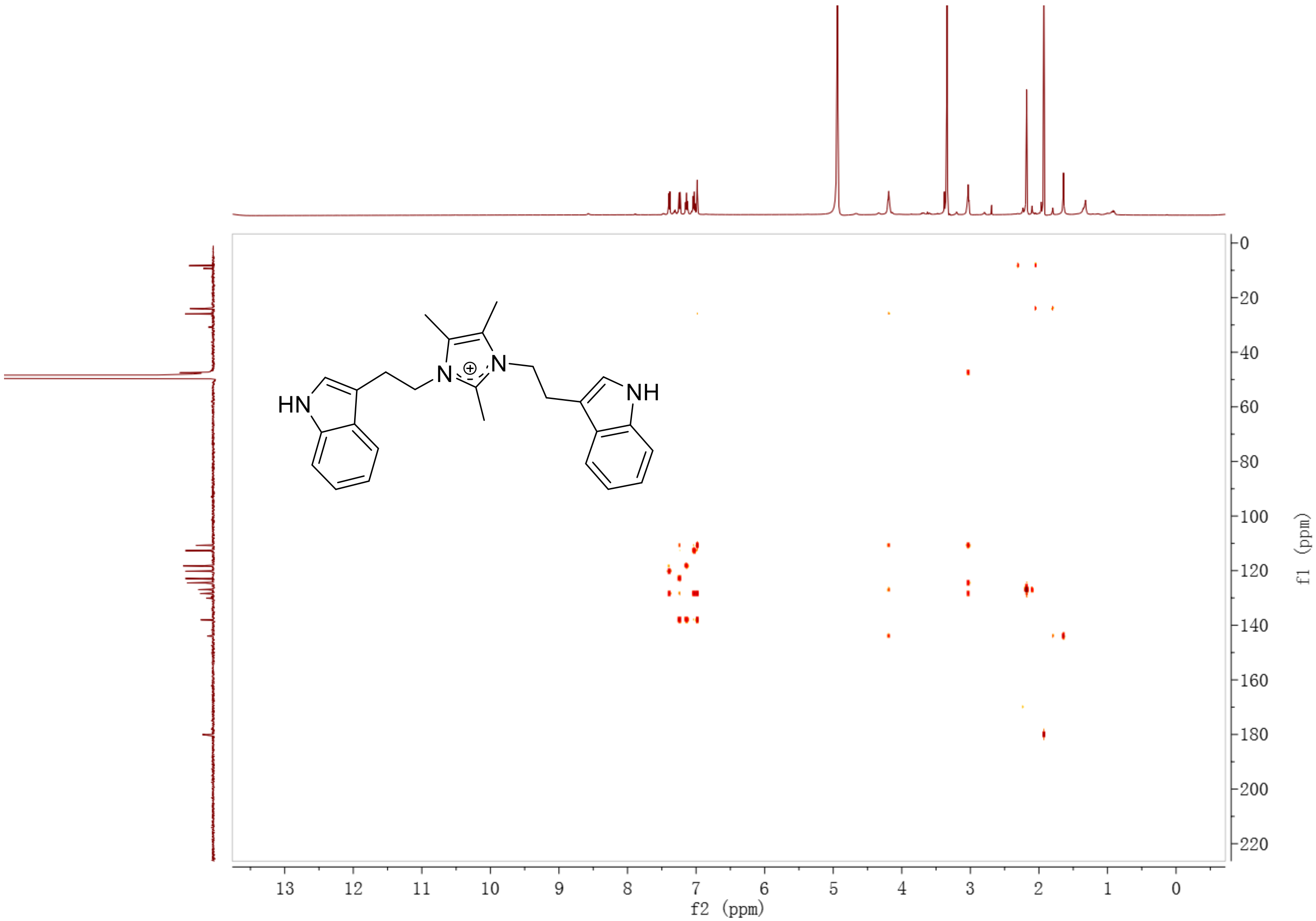

**Figure S27.** $^{1}$H NMR Spectrum of Bacillimidazole E (**5**, 600 MHz, $CD_3OD$)

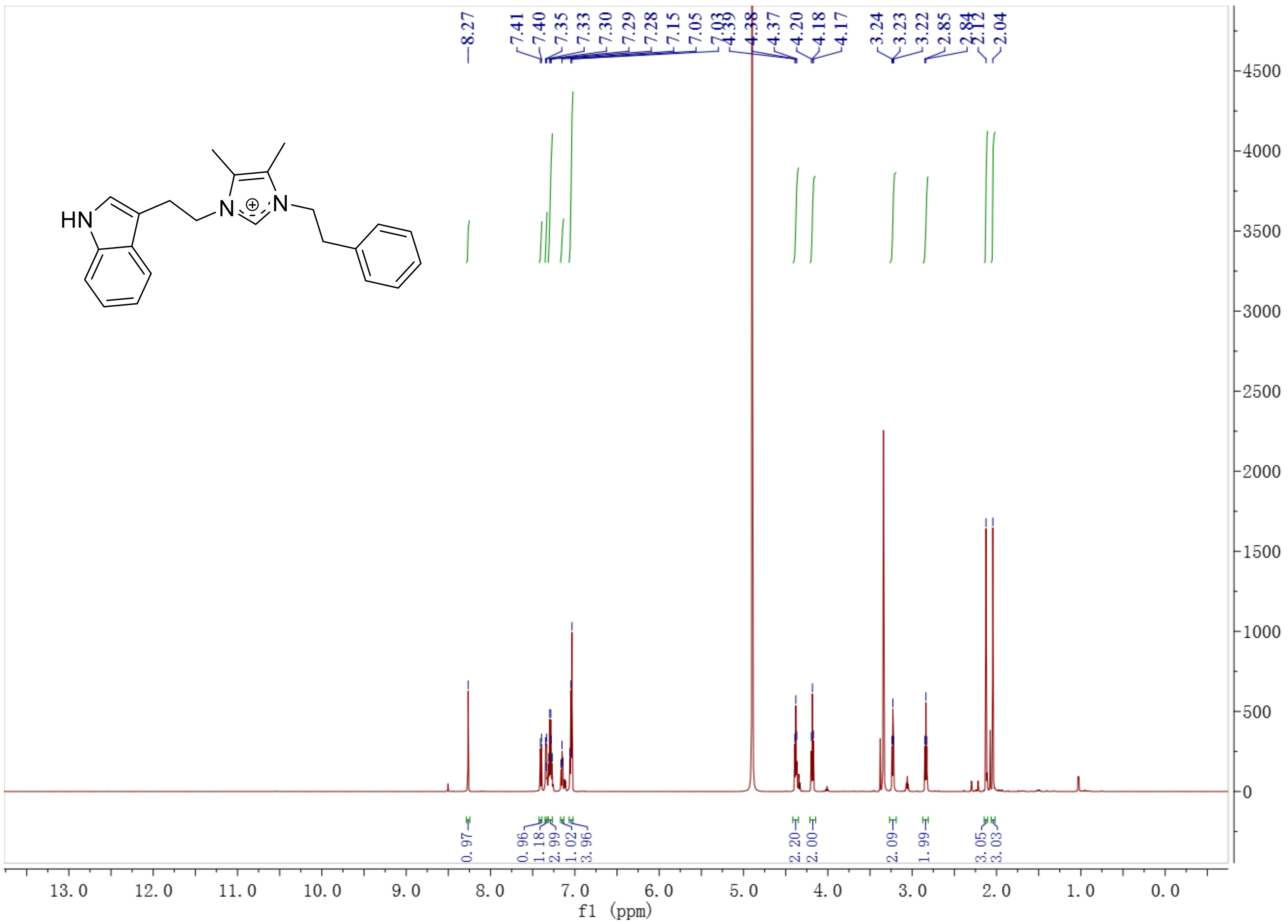

**Figure S28.** $^{13}$C NMR Spectrum of Bacillimidazole E (**5**, 125 MHz, $CD_3OD$)

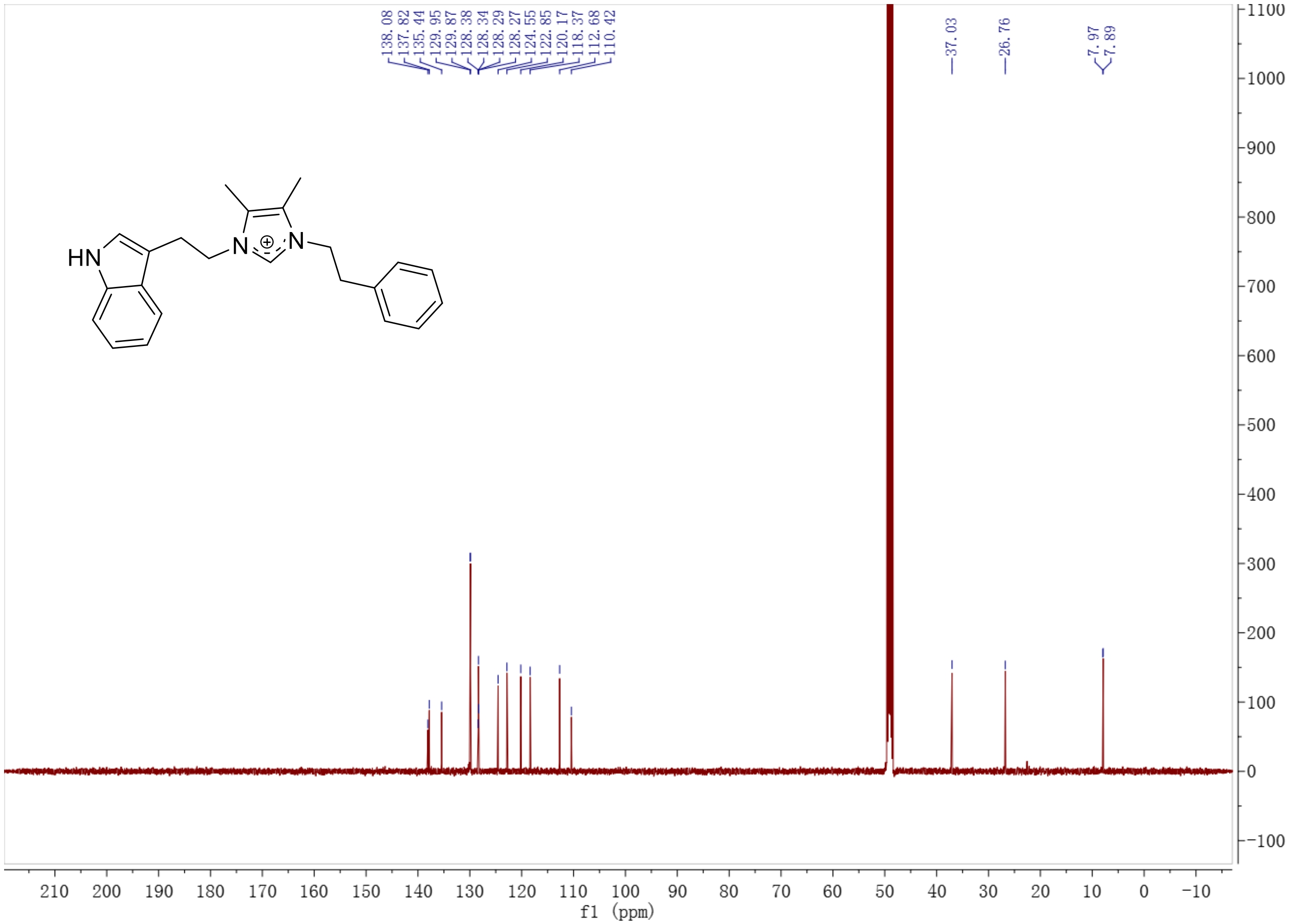

**Figure S29.** gCOSY Spectrum of Bacillimidazole E (**5**, 600 MHz, $CD_3OD$)

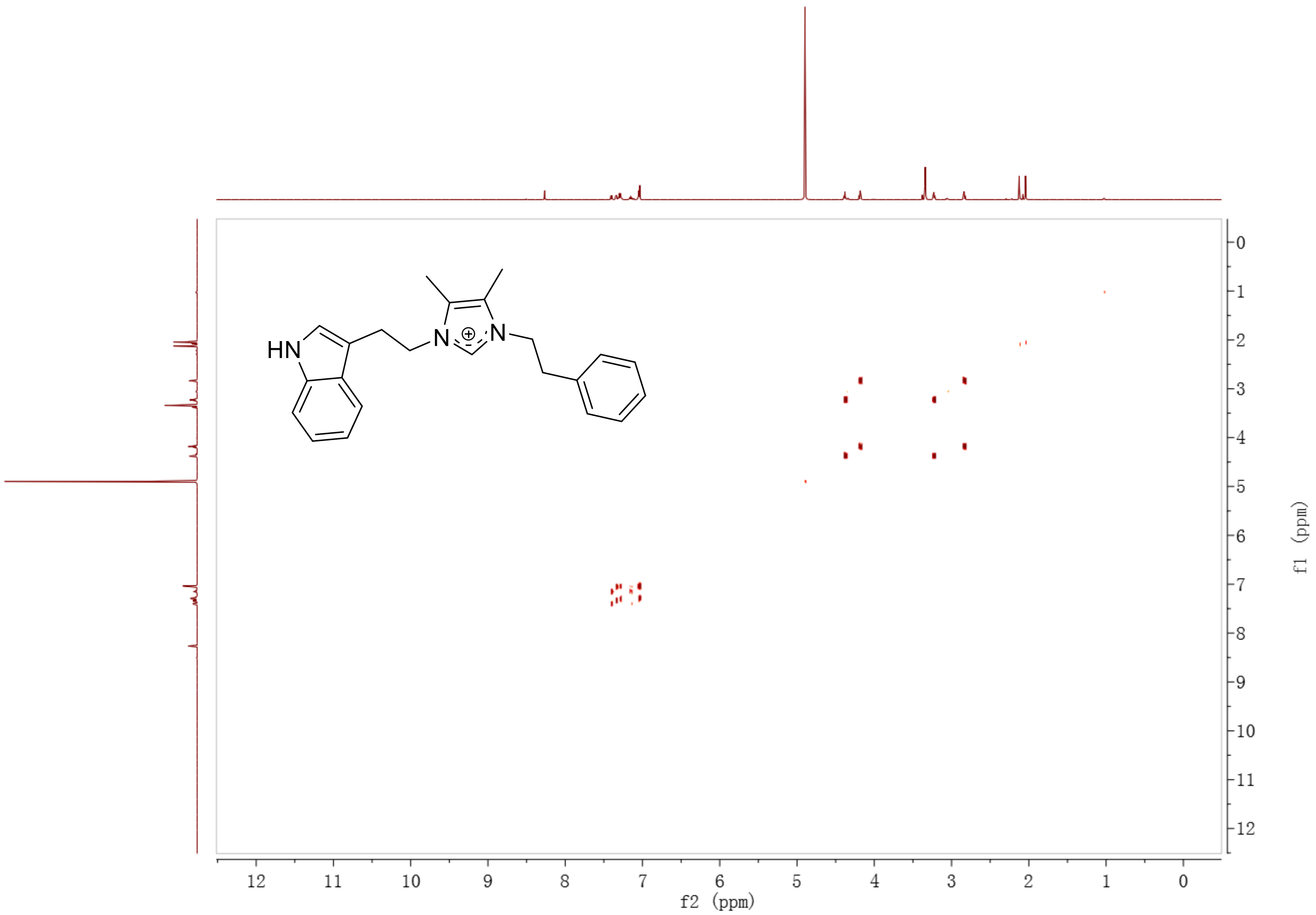

**Figure S30.** gHSQC Spectrum of Bacillimidazole E (**5**, 600 MHz, $CD_3OD$)

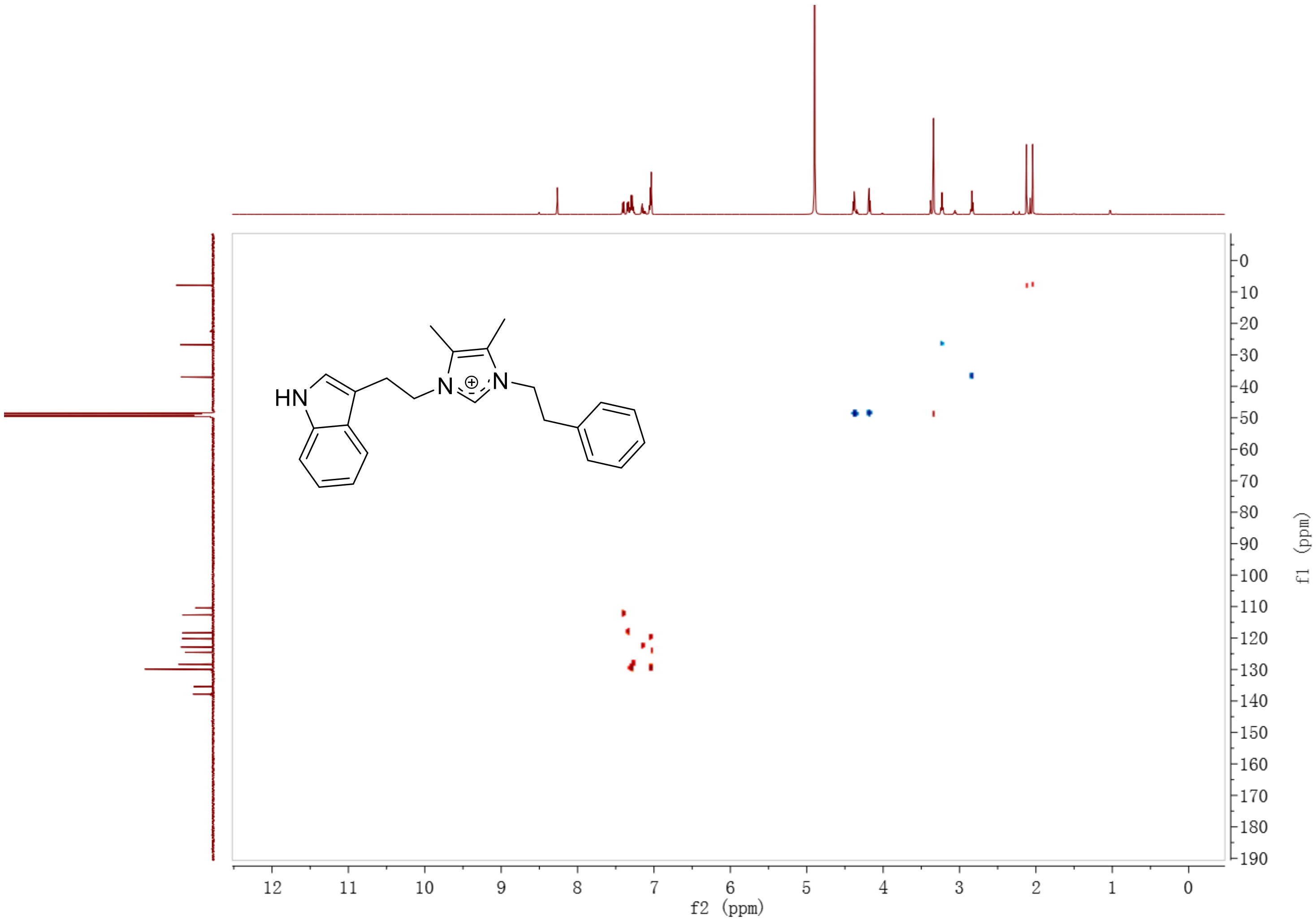

**Figure S31.** gHMBC Spectrum of Bacillimidazole E (**5**, 600 MHz, $CD_3OD$)

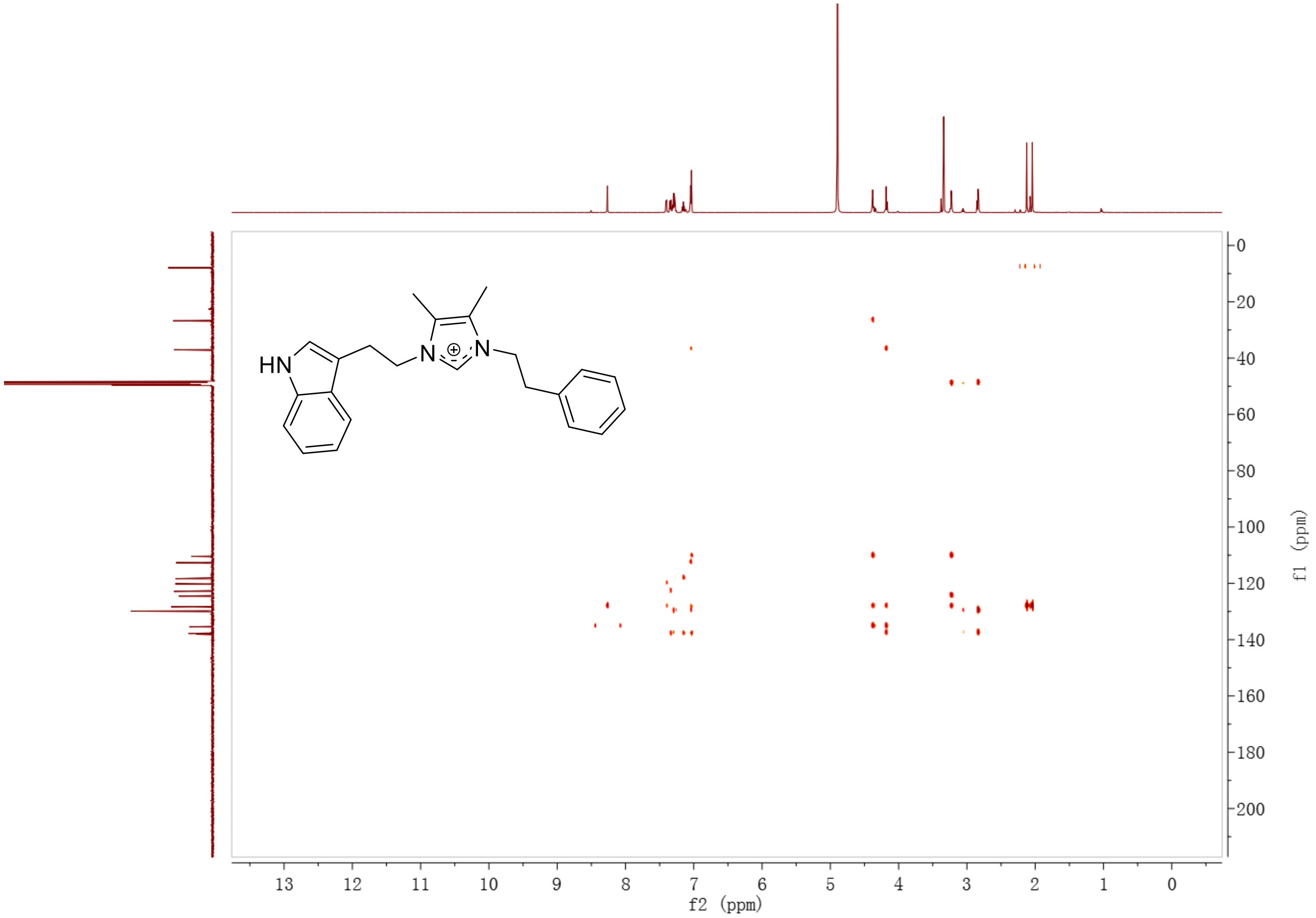

**Figure S32.** $^{1}H$ NMR Spectrum of Bacillimidazole F (**6**, 500 MHz, $CD_3OD$)

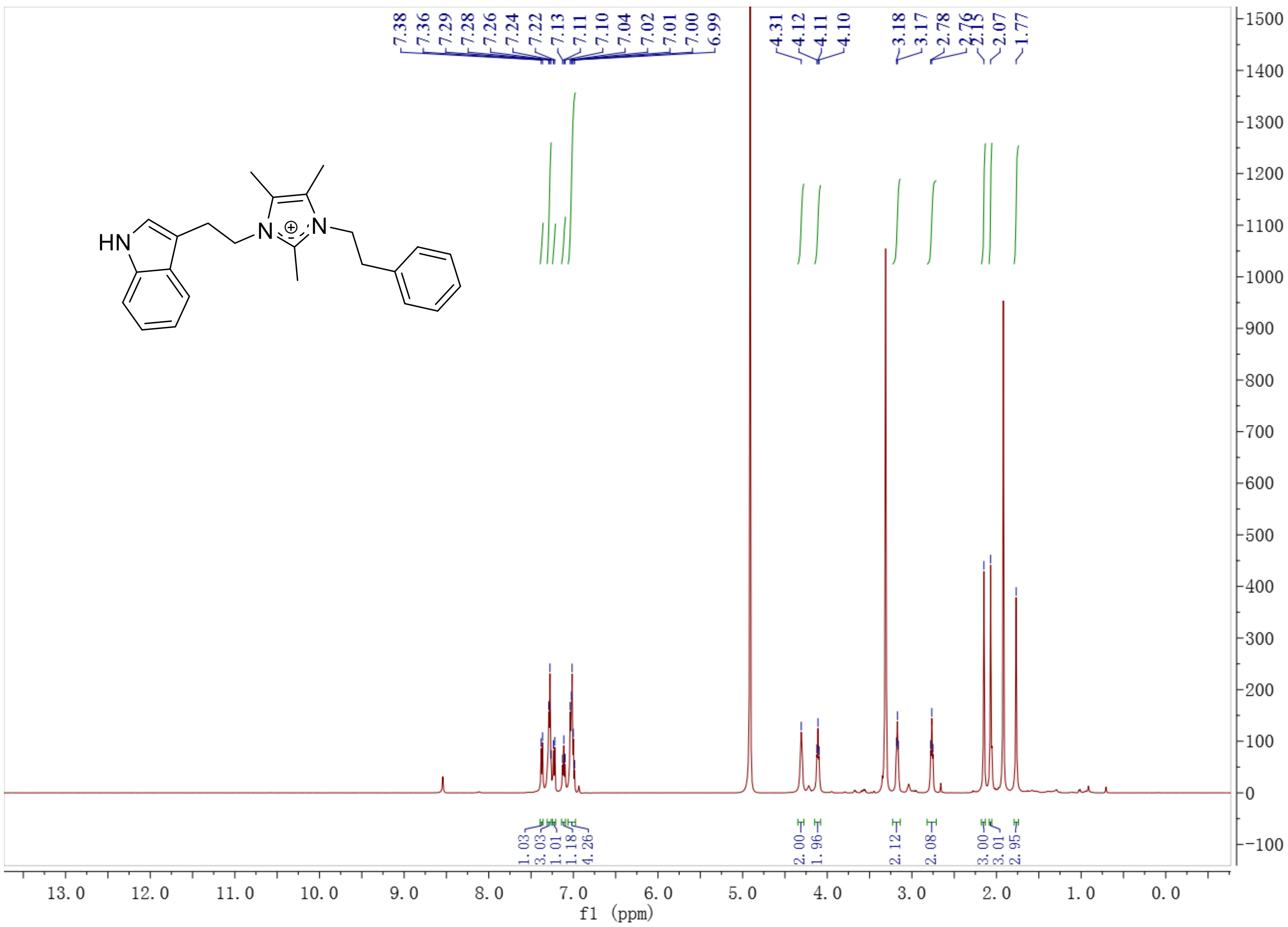

**Figure S33.** $^{13}$C NMR Spectrum of Bacillimidazole F (**6**, 125 MHz, $CD_3OD$)

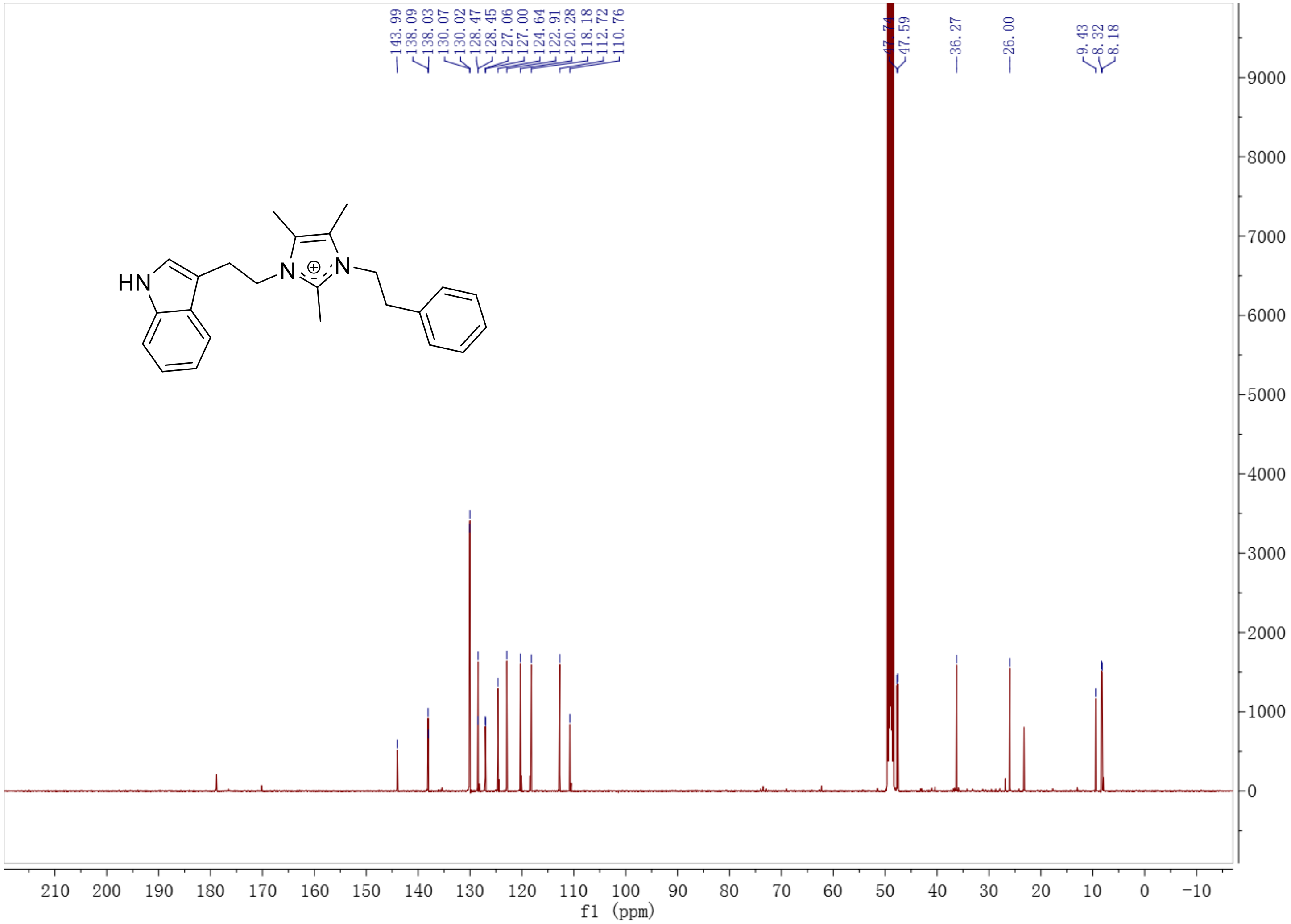

**Figure S34.** gCOSY Spectrum of Bacillimidazole F (**6**, 500 MHz, $CD_3OD$)

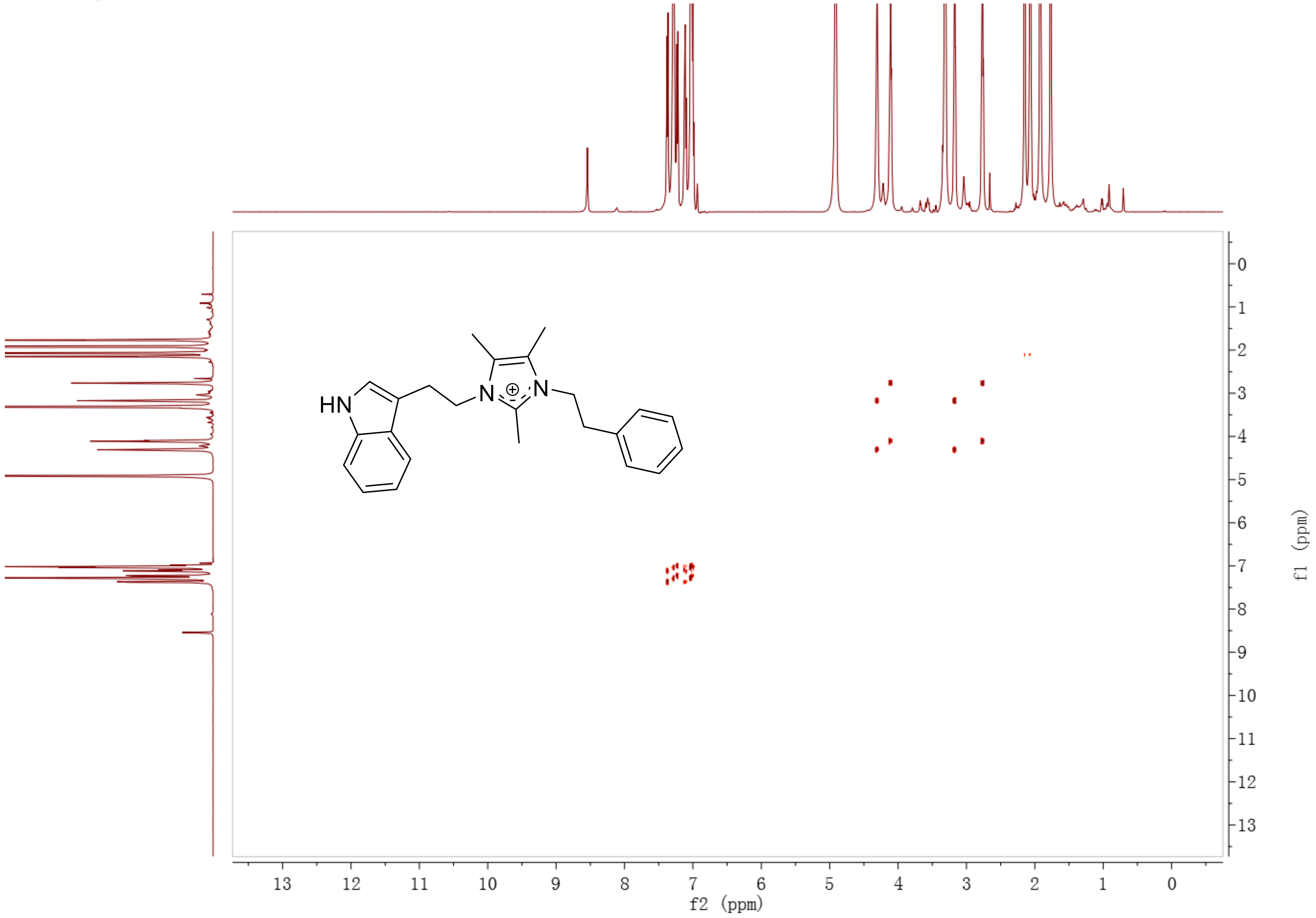

**Figure S35.** gHSQC Spectrum of Bacillimidazole F (**6**, 500 MHz, $CD_3OD$)

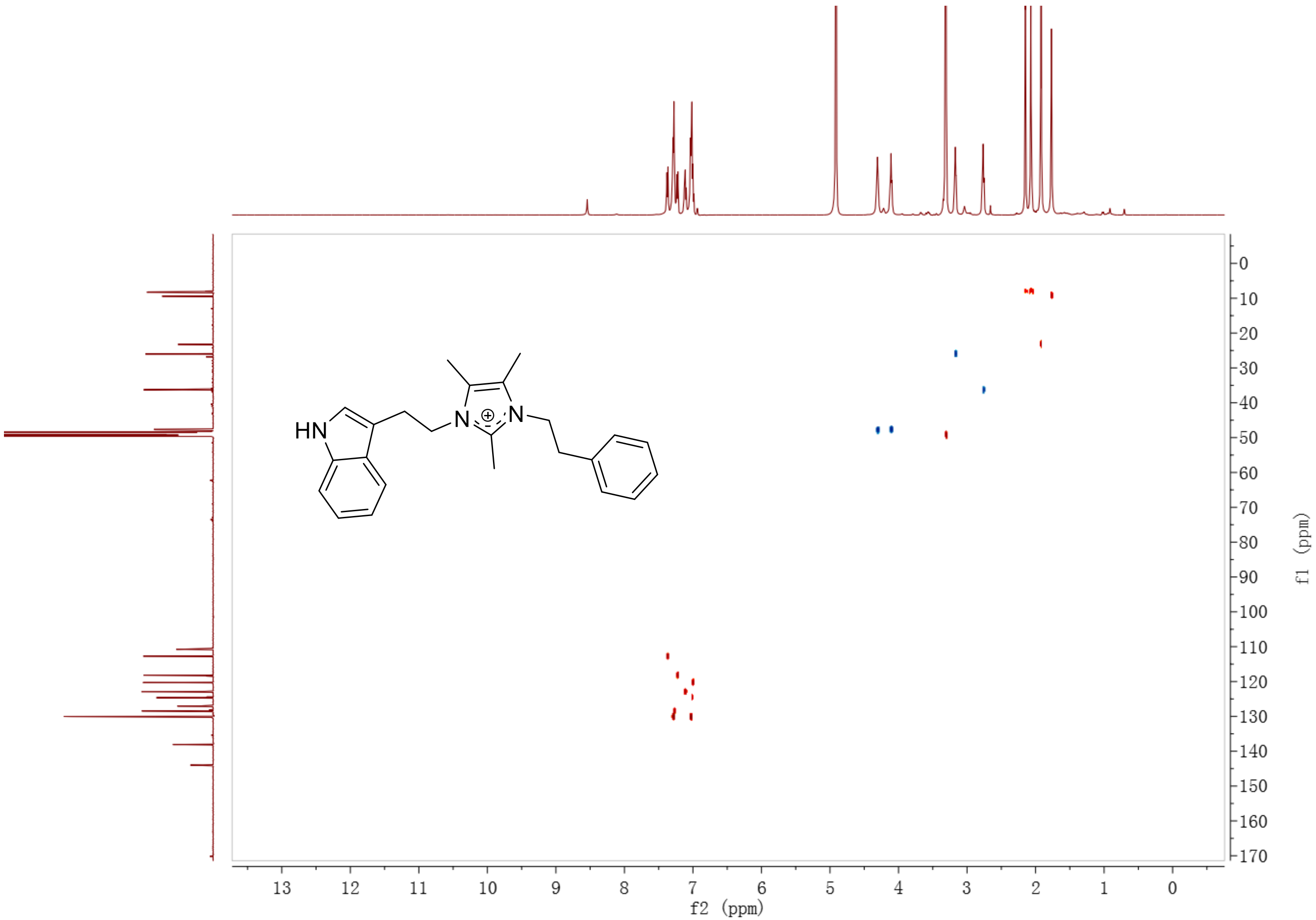

**Figure S36.** gHMBC Spectrum of Bacillimidazole F (**6**, 500 MHz, $CD_3OD$)

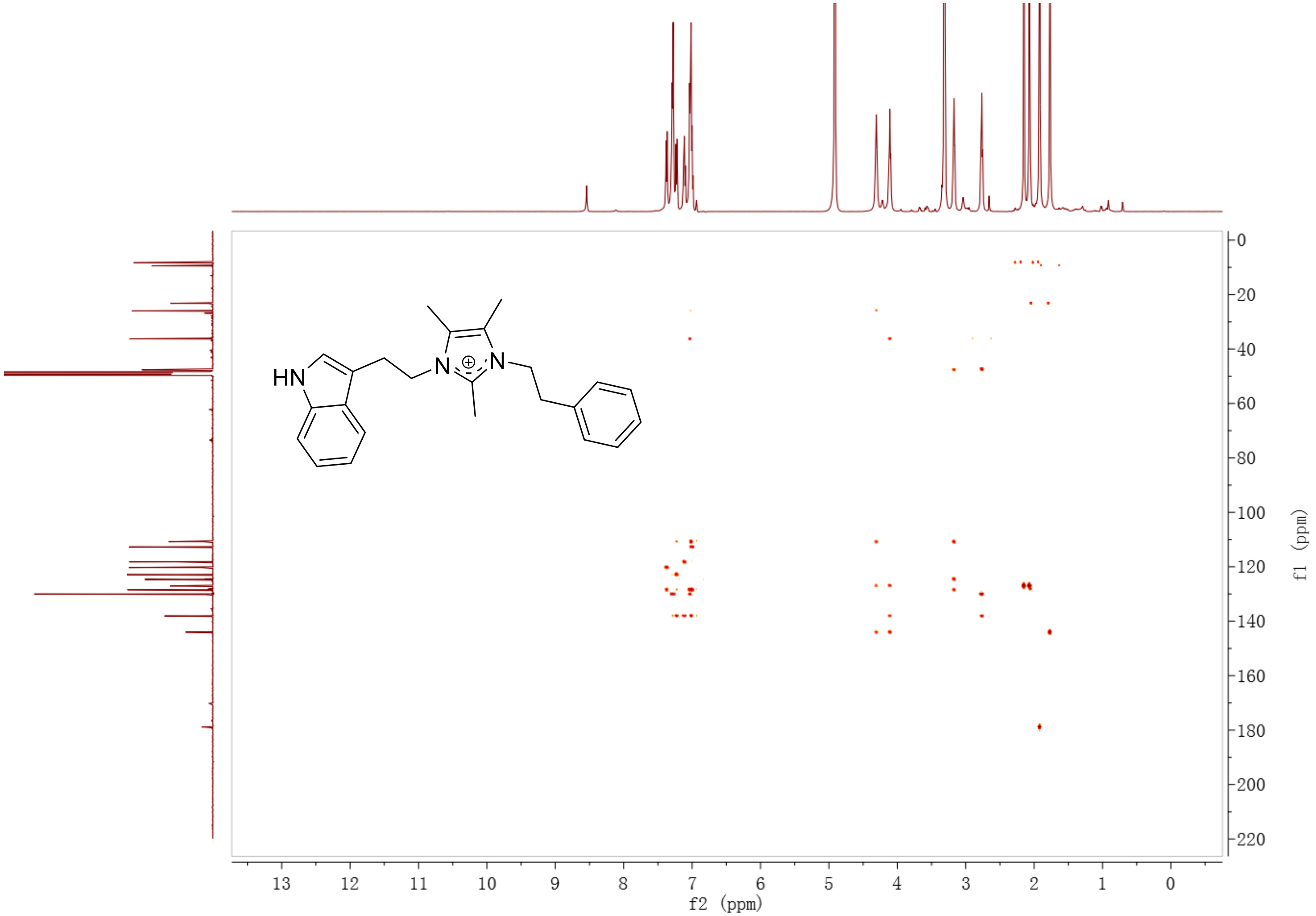

**Figure S37.** Positive Ion HRESIMS of Bacillimidazole A (**1**)

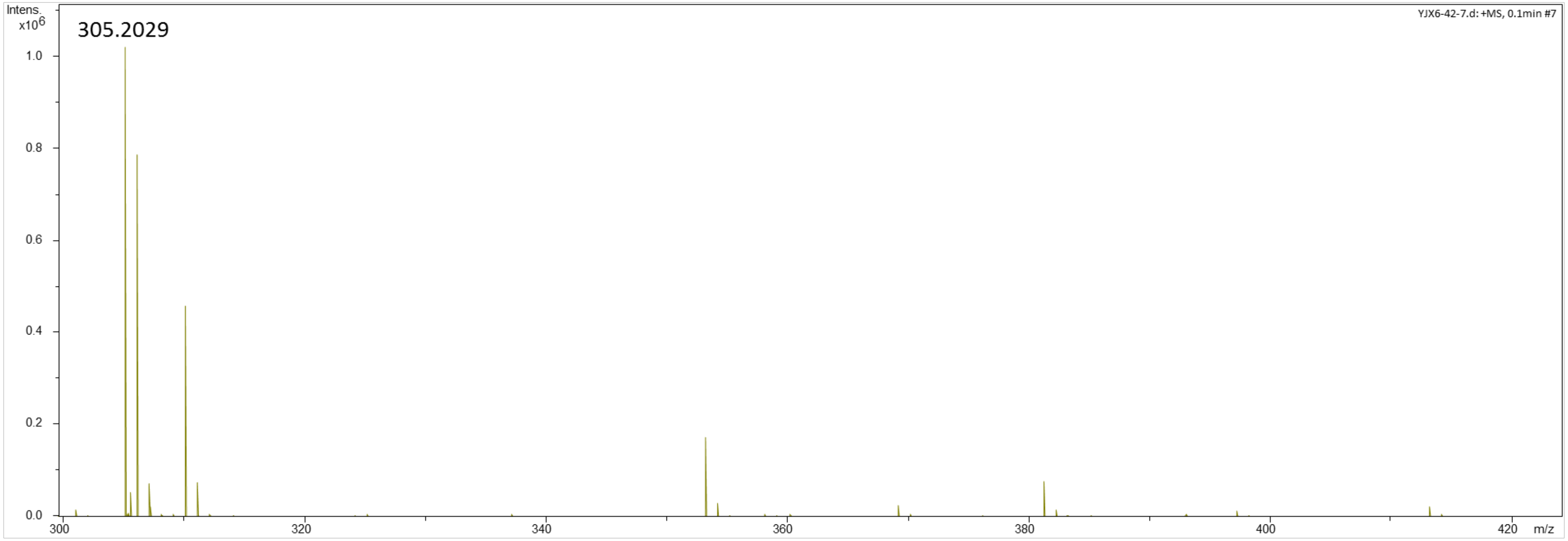

**Figure S38.** Positive Ion HRESIMS of Bacillimidazole B (**2**)

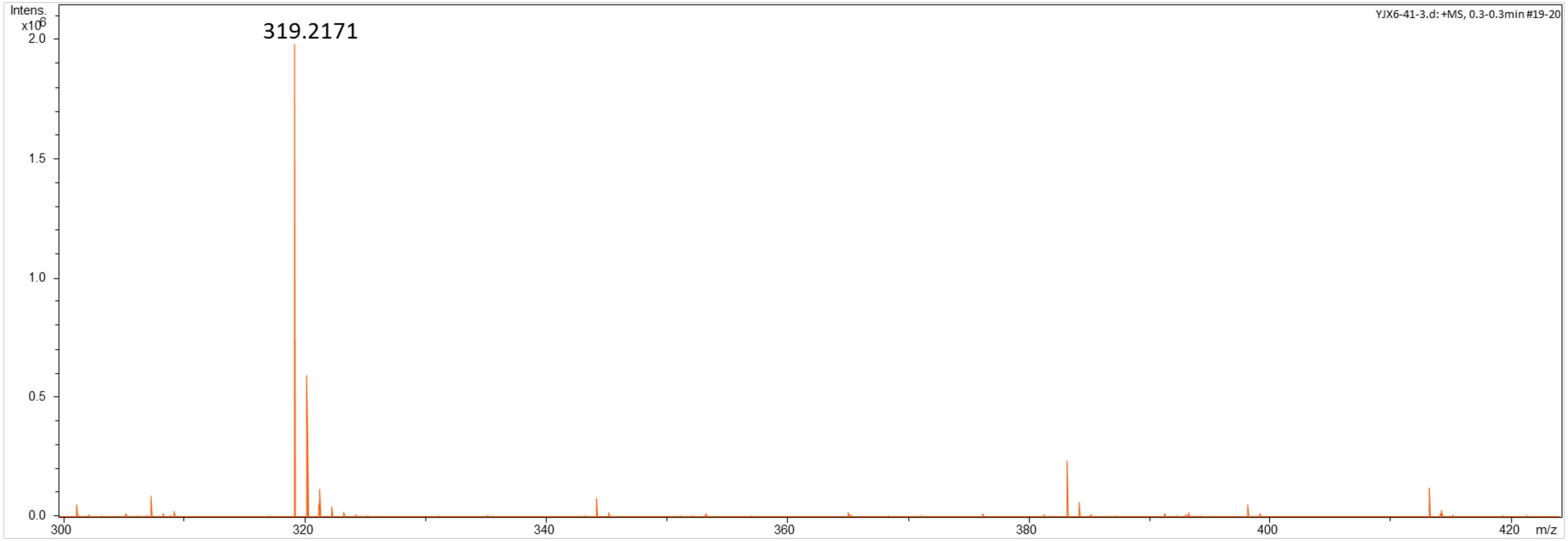

**Figure S39.** Positive Ion HRESIMS of Bacillimidazole C (**3**)

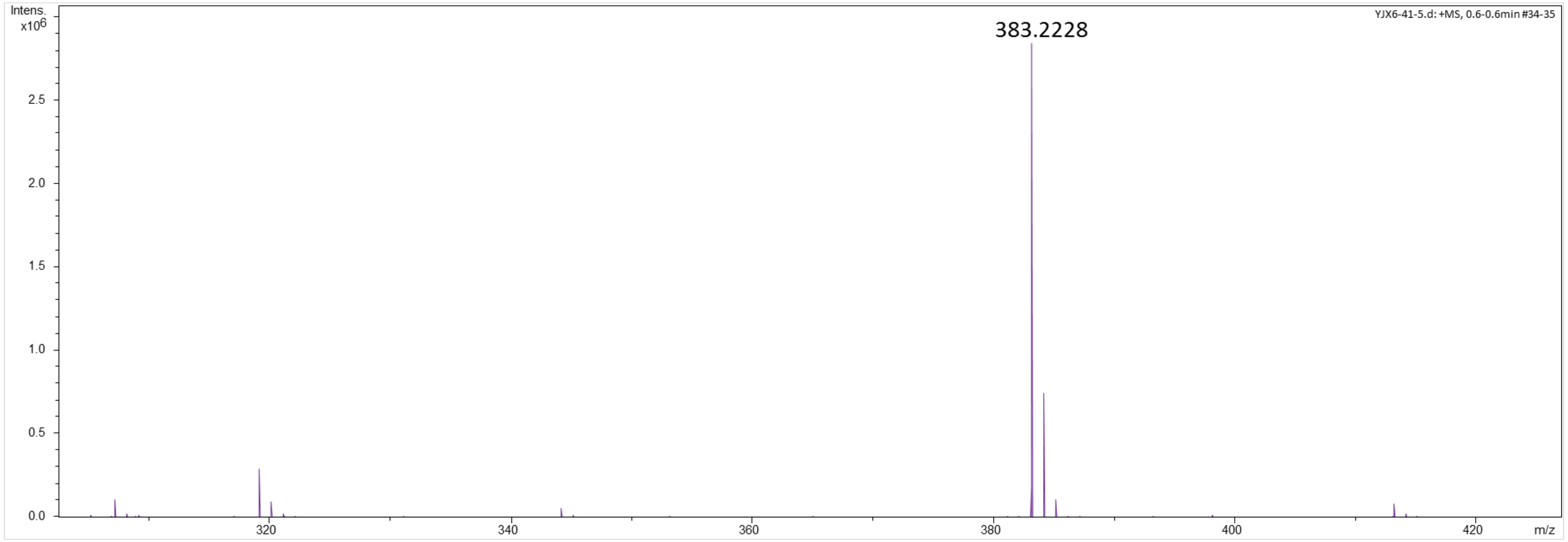

**Figure S40.** Positive Ion HRESIMS of Bacillimidazole D (**4**)

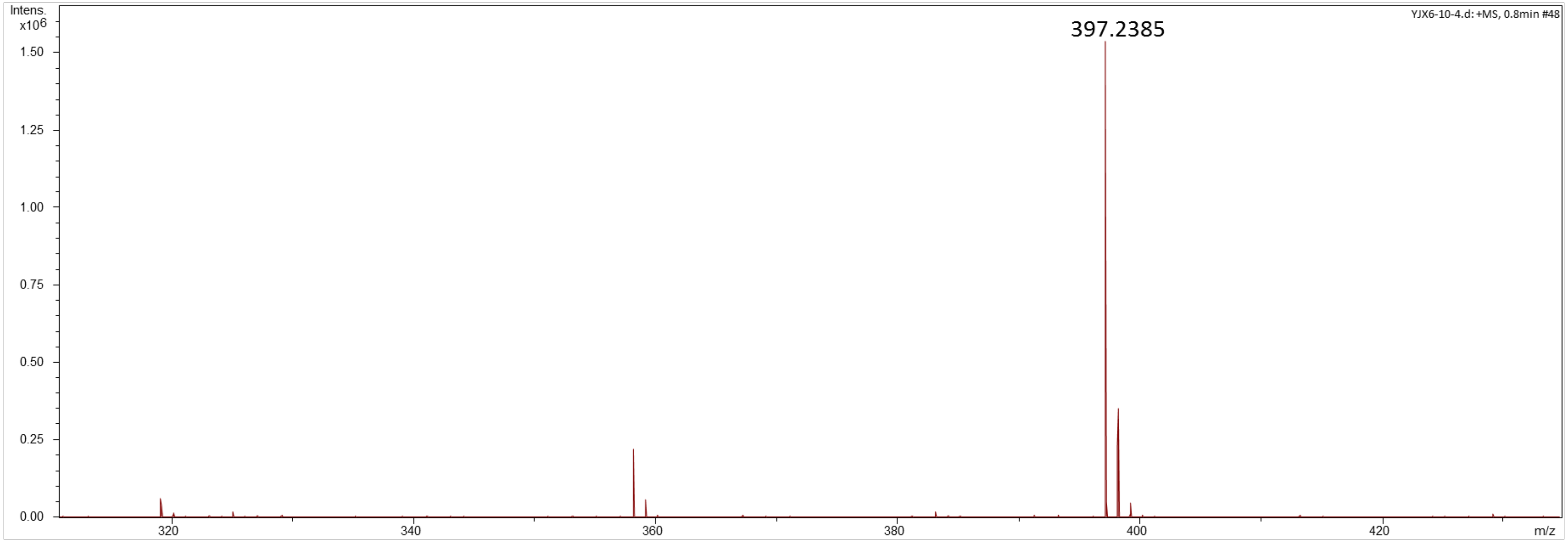

**Figure S41.** Positive Ion HRESIMS of Bacillimidazole E (**5**)

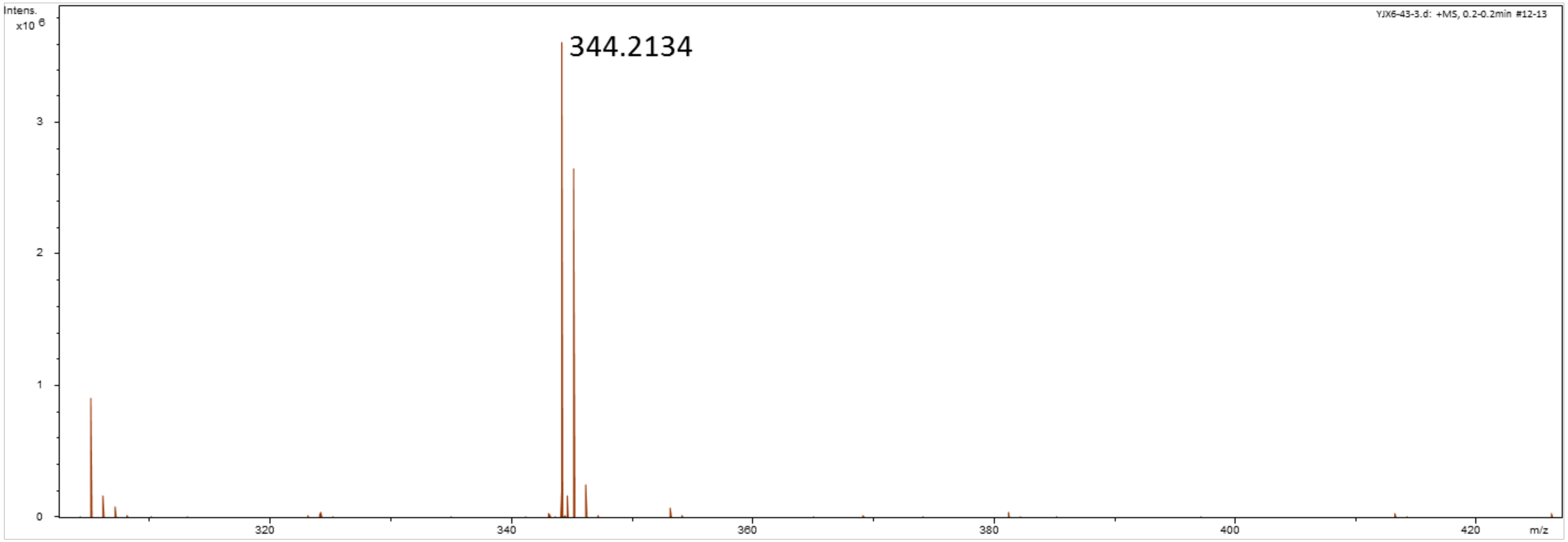

**Figure S42.** Positive Ion HRESIMS of Bacillimidazole F (**6**)

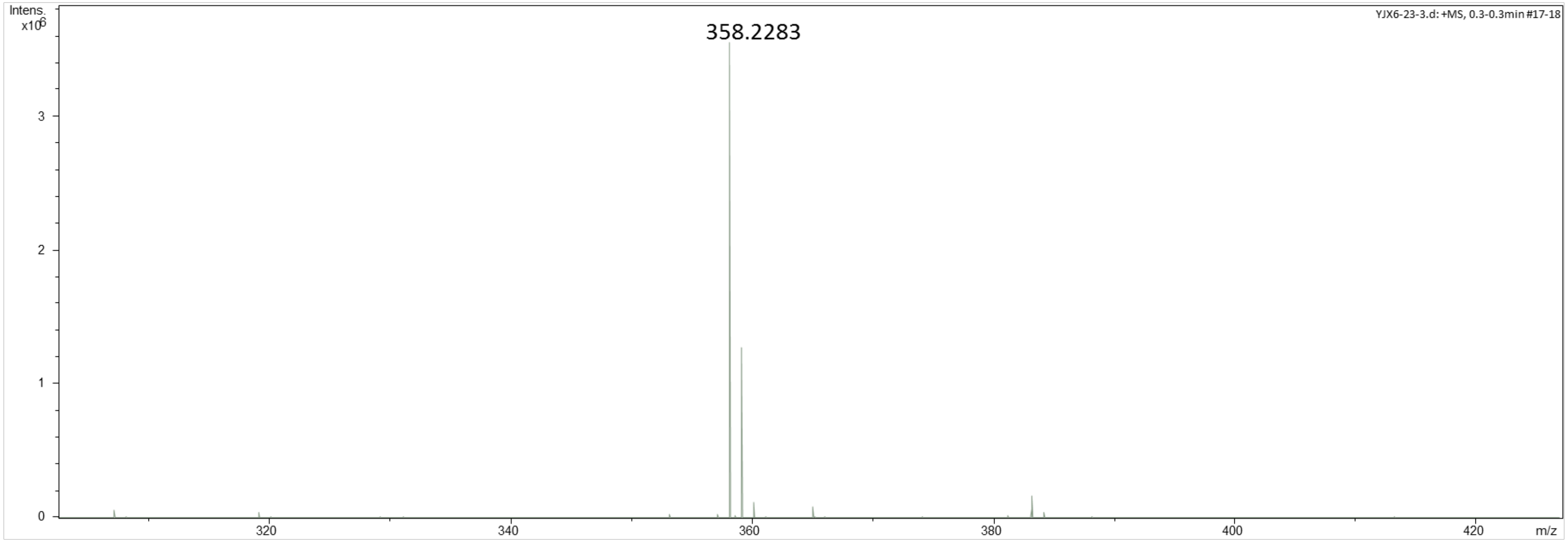

**Figure S43.** $^{1}$H NMR Spectrum of $^{13}$C Labeled Bacillimidazole C (**3**, 500 MHz, $CD_3OD$)

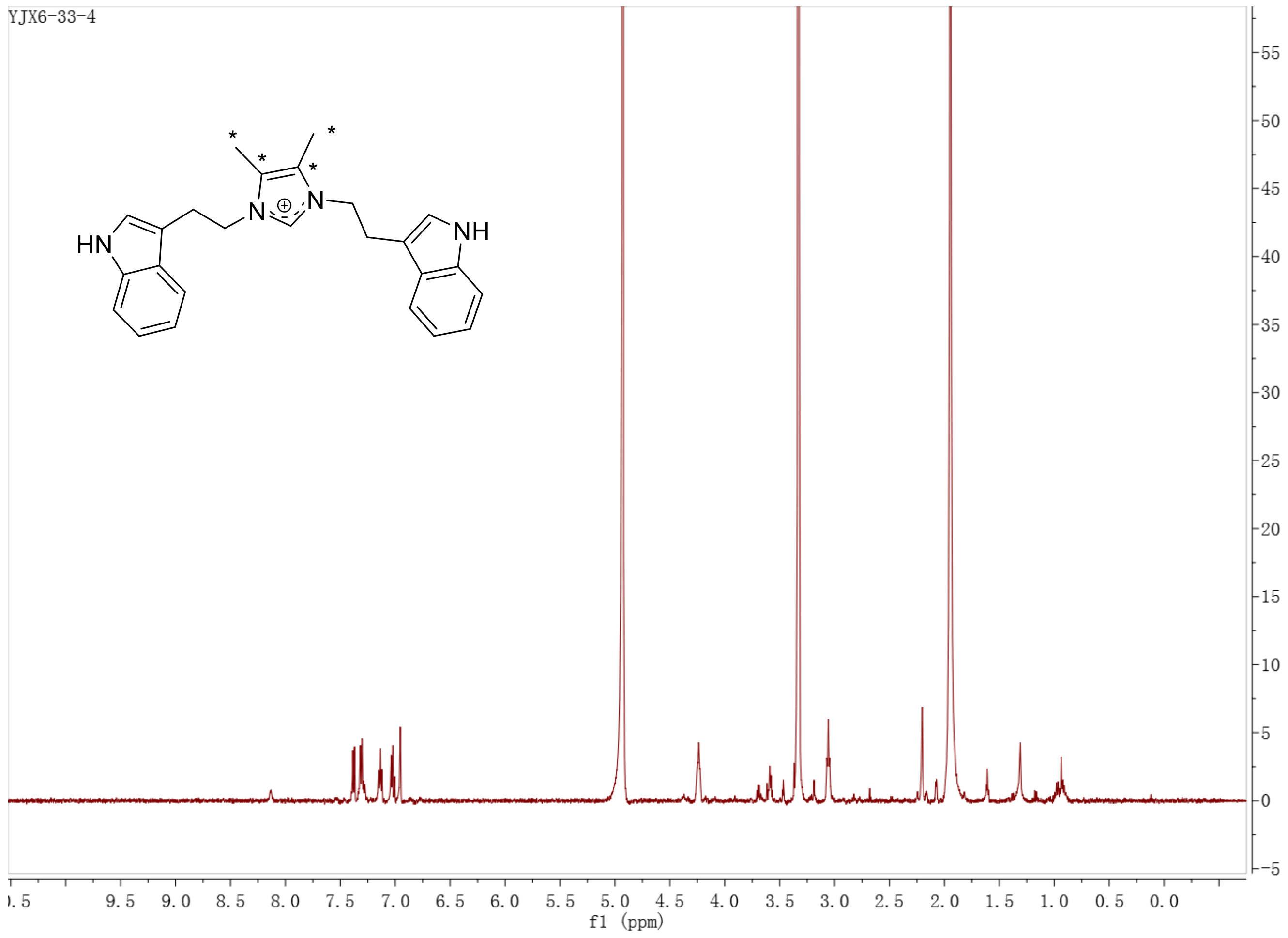

**Figure S44.** $^{13}$C NMR Spectrum of $^{13}$C Labeled Bacillimidazole C (**3**, 125 MHz, $CD_3OD$)

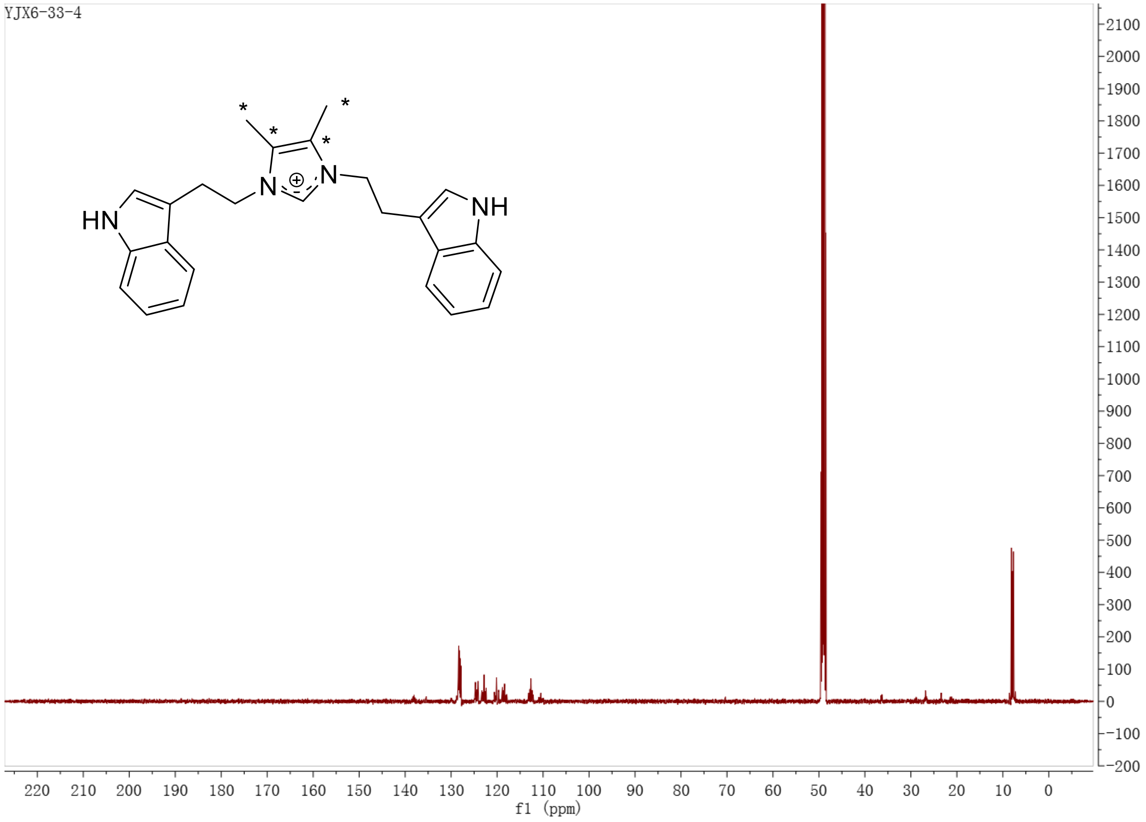

**Figure S45.** $^{1}H$ NMR Spectrum of $^{13}C$ Labeled Bacillimidazole E (**5**, 500 MHz, $CD_3OD$)

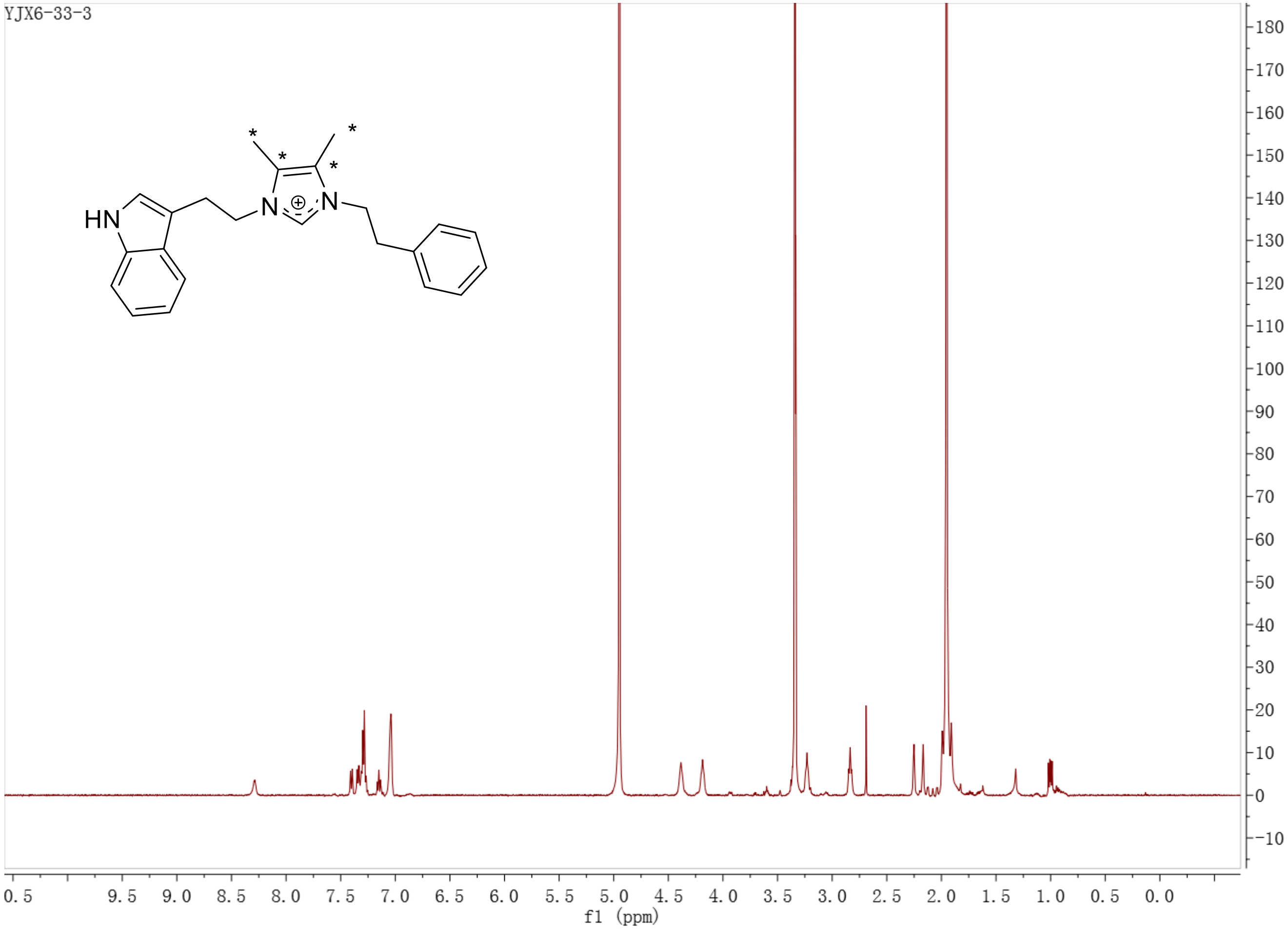

**Figure S46.** $^{13}$C NMR Spectrum of $^{13}$C Labeled Bacillimidazole E (**5**, 125 MHz, $CD_3OD$)

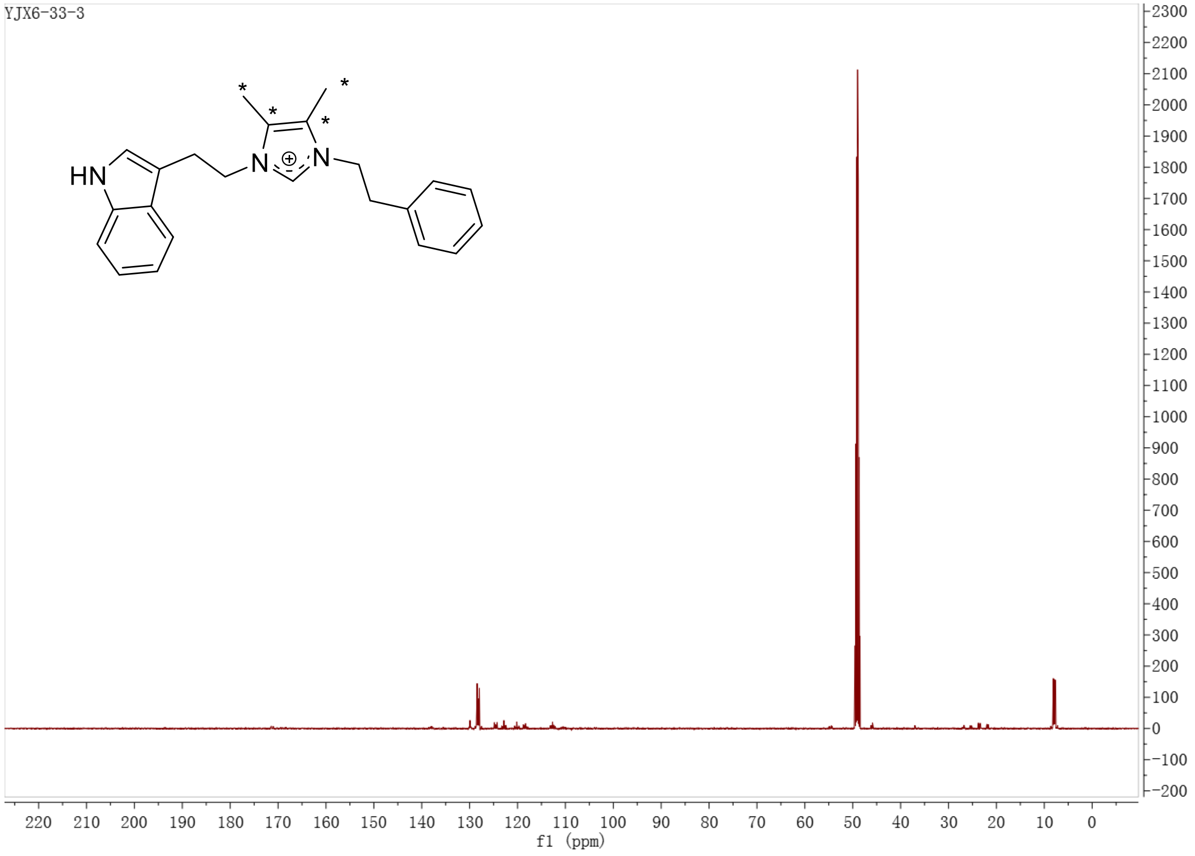

**Figure S47.** Positive Ion HRESIMS of $^{13}$C Enriched Bacillimidazole C (**3**)

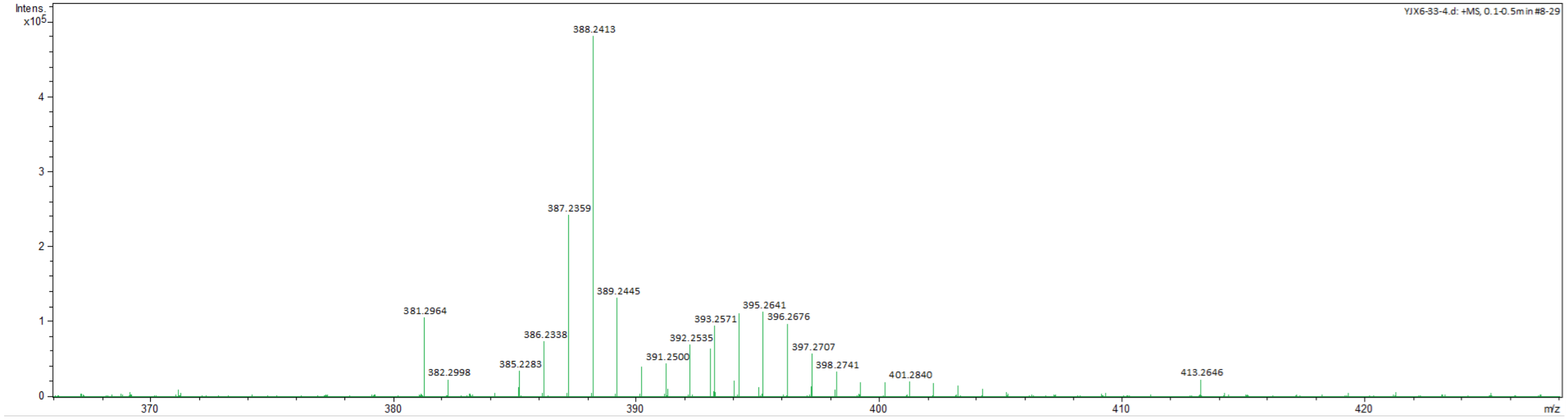

**Figure S48.** Positive Ion HRESIMS of $^{13}$C Enriched Bacillimidazole E (**5**)

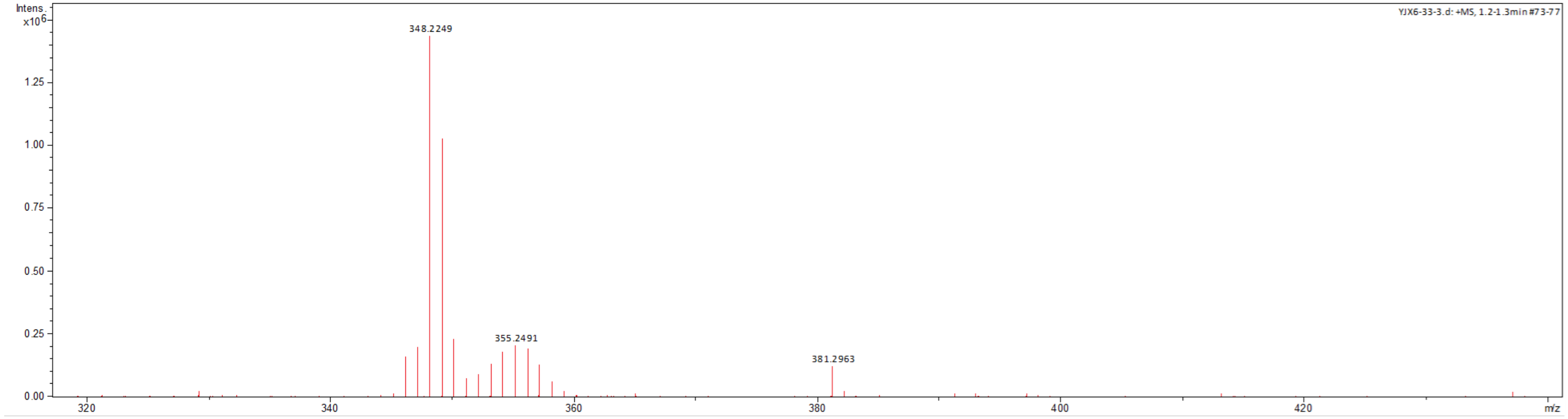

**Figure S49.** LC-MS analysis of strain *Bacillus* sp. WMMC325. EIC for bacillimidazoles A−F were 305, 319, 383, 397, 344, 358, respectively

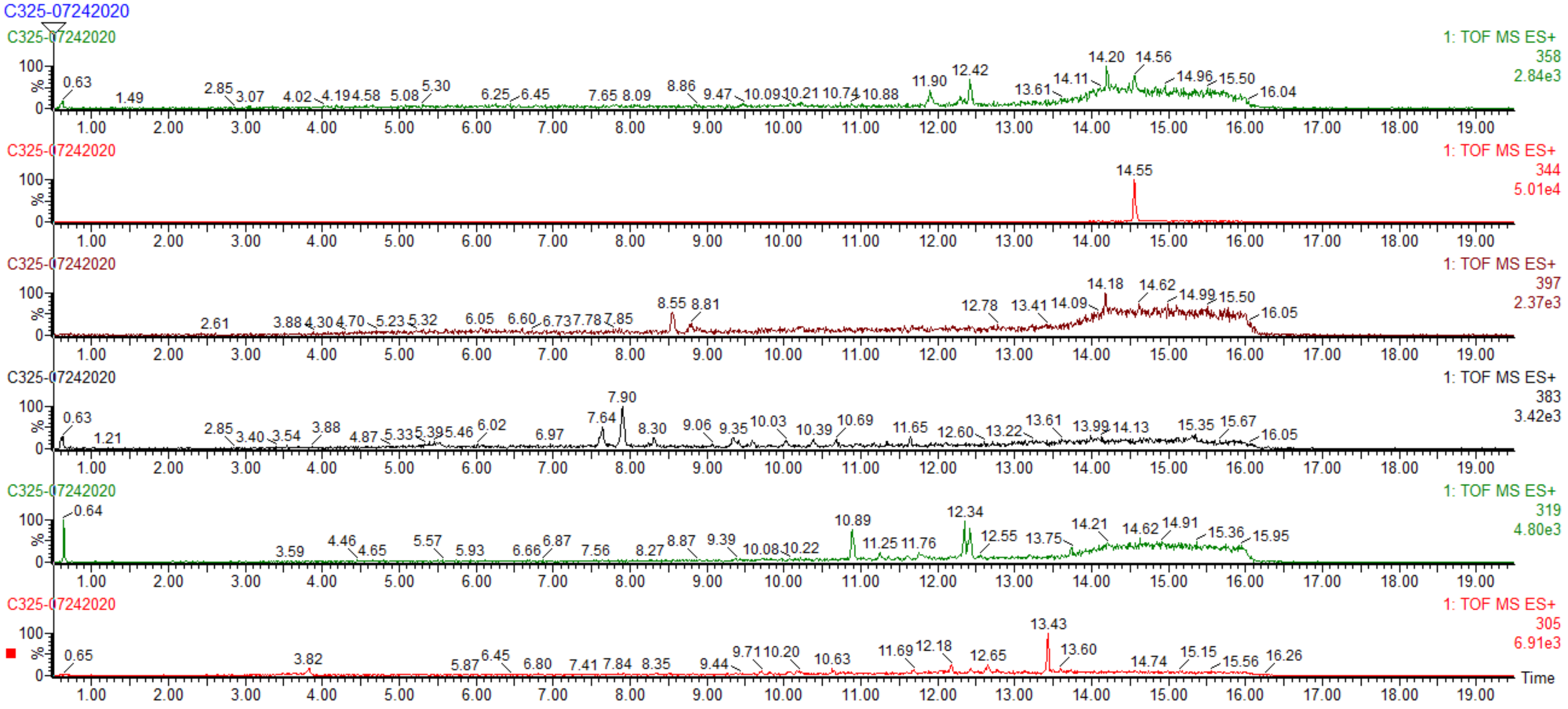

**Figure S50.** LC-MS analysis of strain *Bacillus* sp. WMMC331. EIC for bacillimidazoles A−F were 305, 319, 383, 397, 344, 358, respectively.

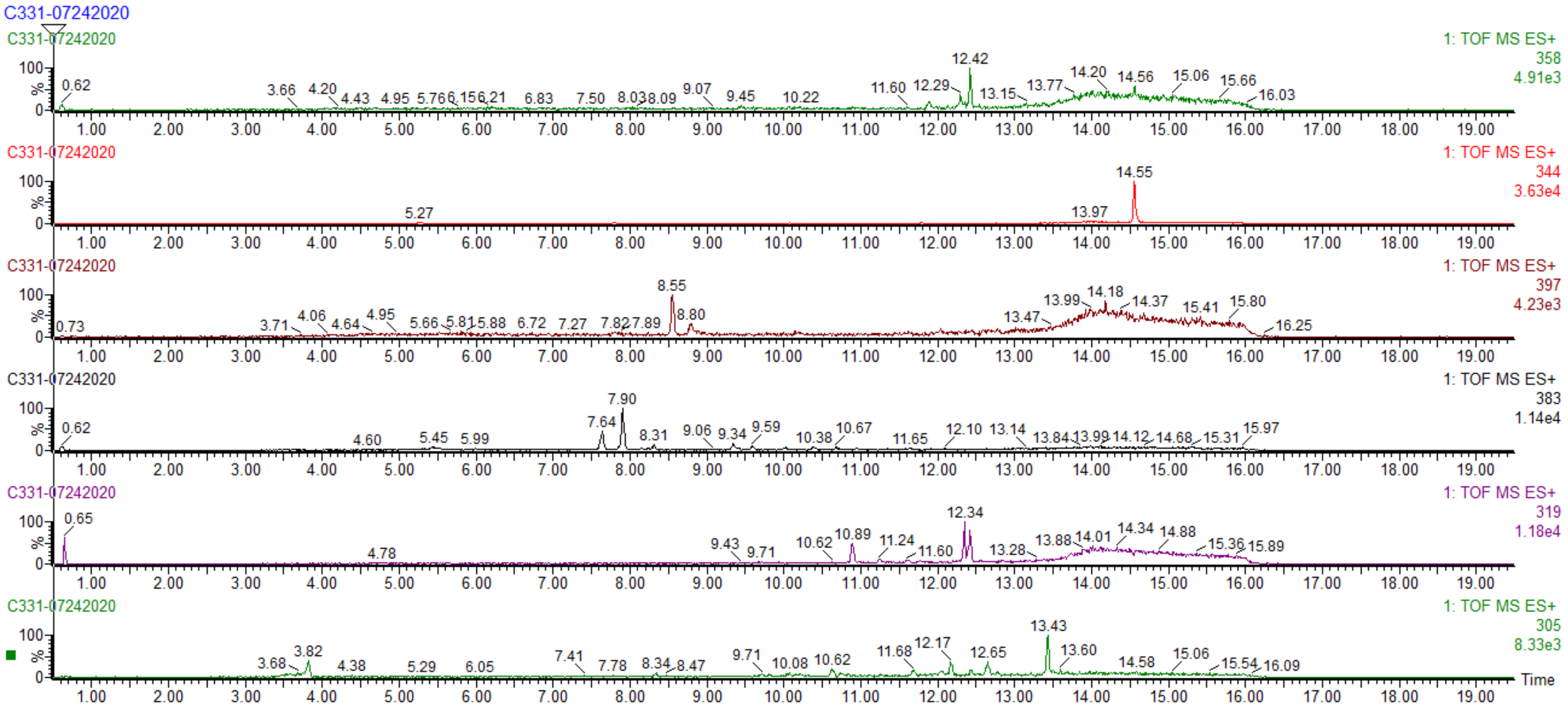

**Figure S51.** LC-MS analysis of strain *Bacillus* sp. WMMC1349. EIC for bacillimidazoles A−F were 305, 319, 383, 397, 344, 358, respectively.

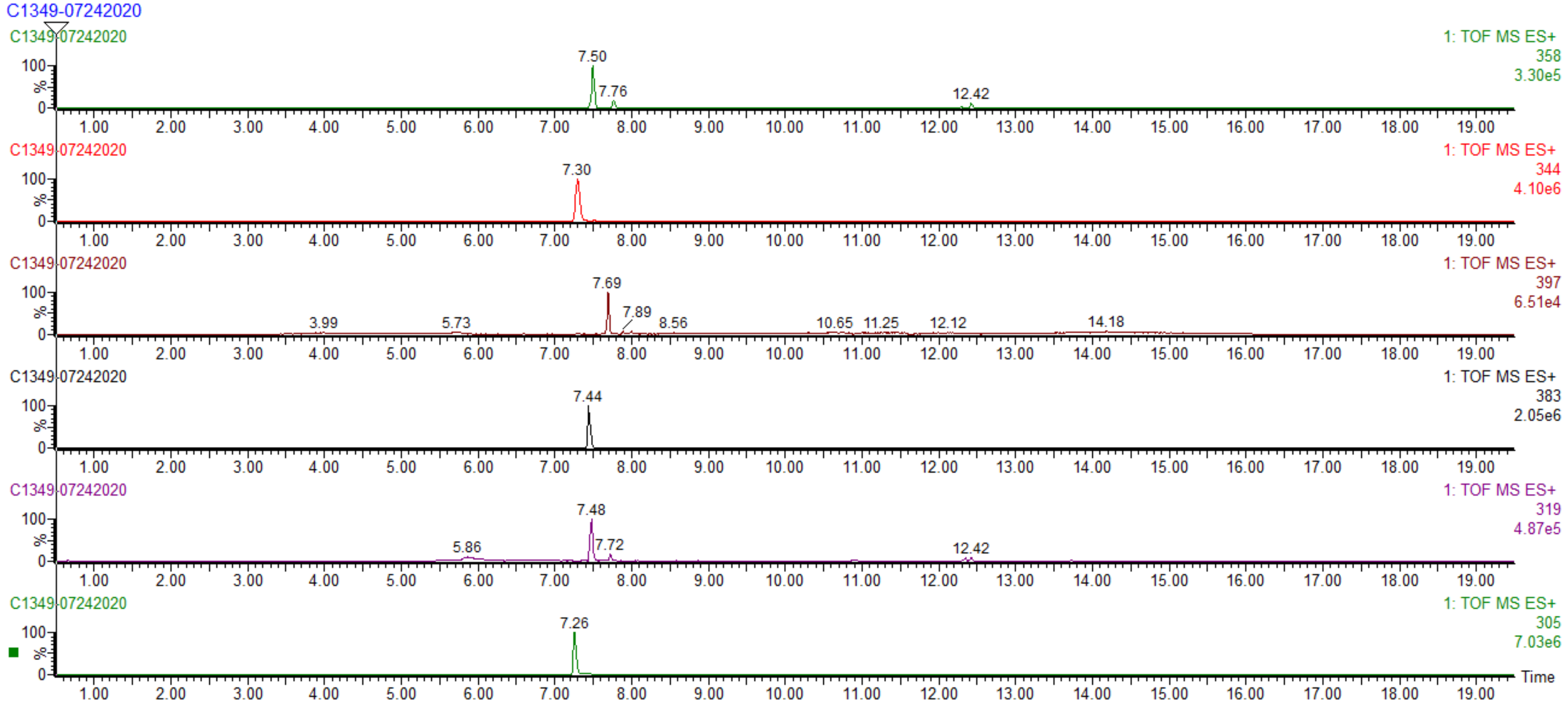

**Figure S52.** LC-MS analysis of strain *Bacillus* sp. WMMC1350. EIC for bacillimidazoles A−F were 305, 319, 383, 397, 344, 358, respectively.

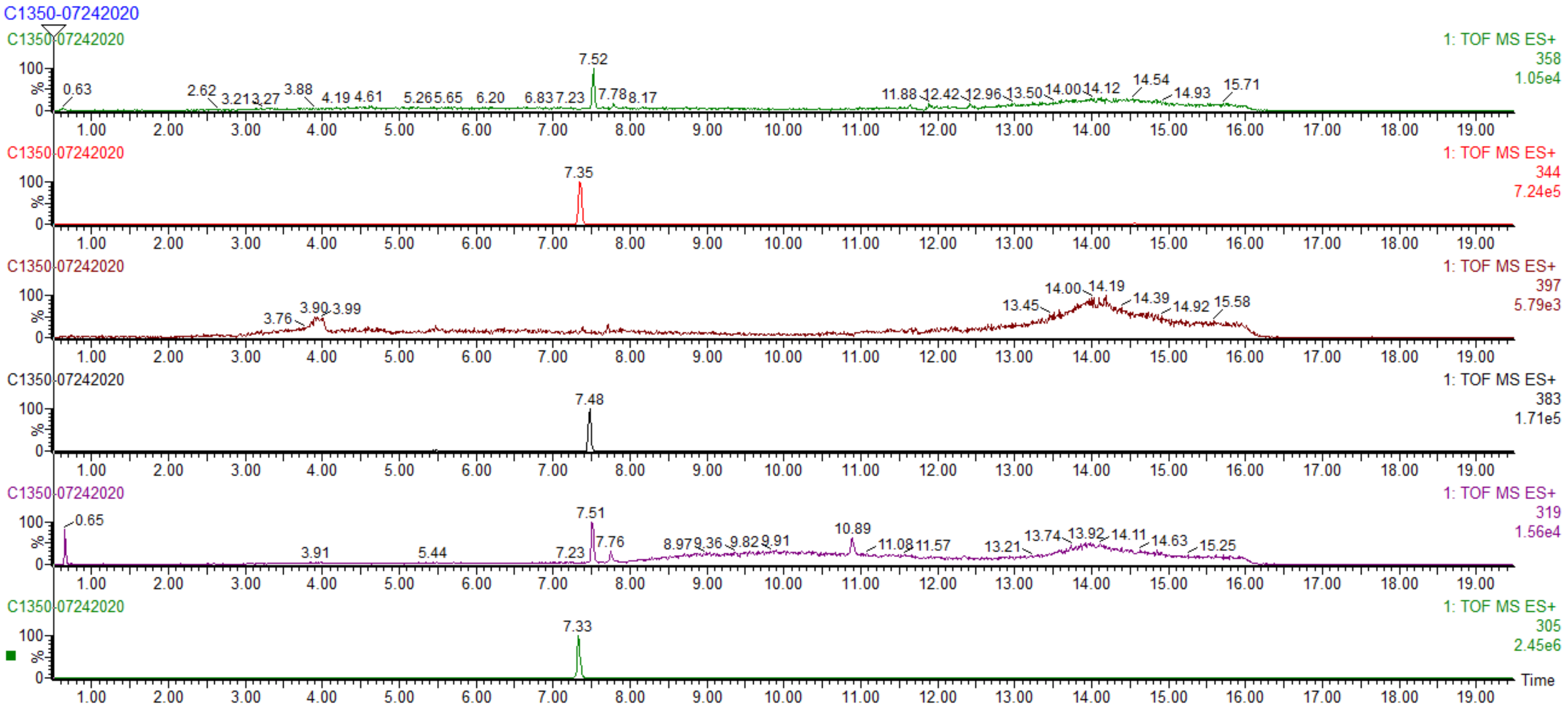

**Figure S53.** LC-MS analysis of strain *Bacillus* sp. WMMC1351. EIC for bacillimidazoles A−F were 305, 319, 383, 397, 344, 358, respectively.

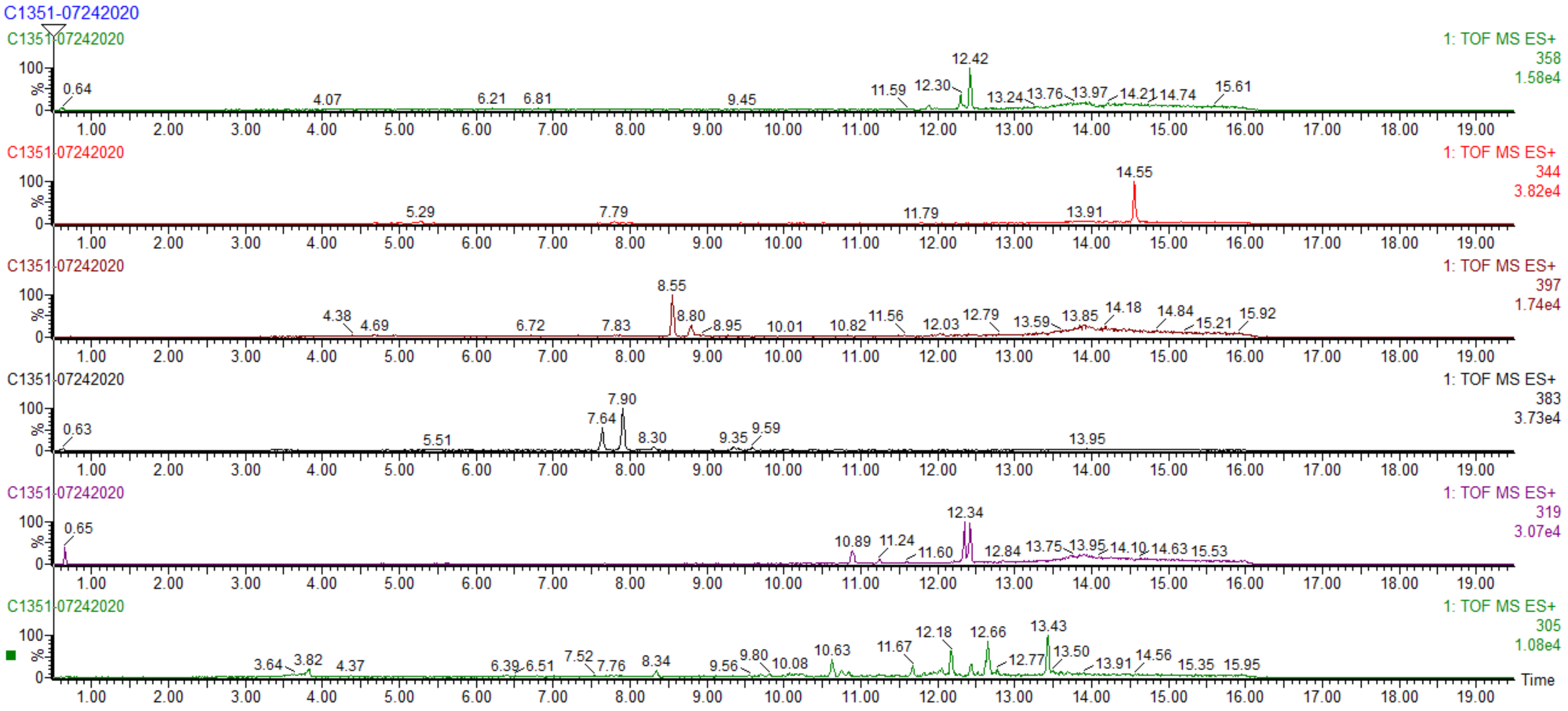

**Figure S54.** LC-MS analysis of strain *Bacillus* sp. WMMC1352. EIC for bacillimidazoles A−F were 305, 319, 383, 397, 344, 358, respectively.

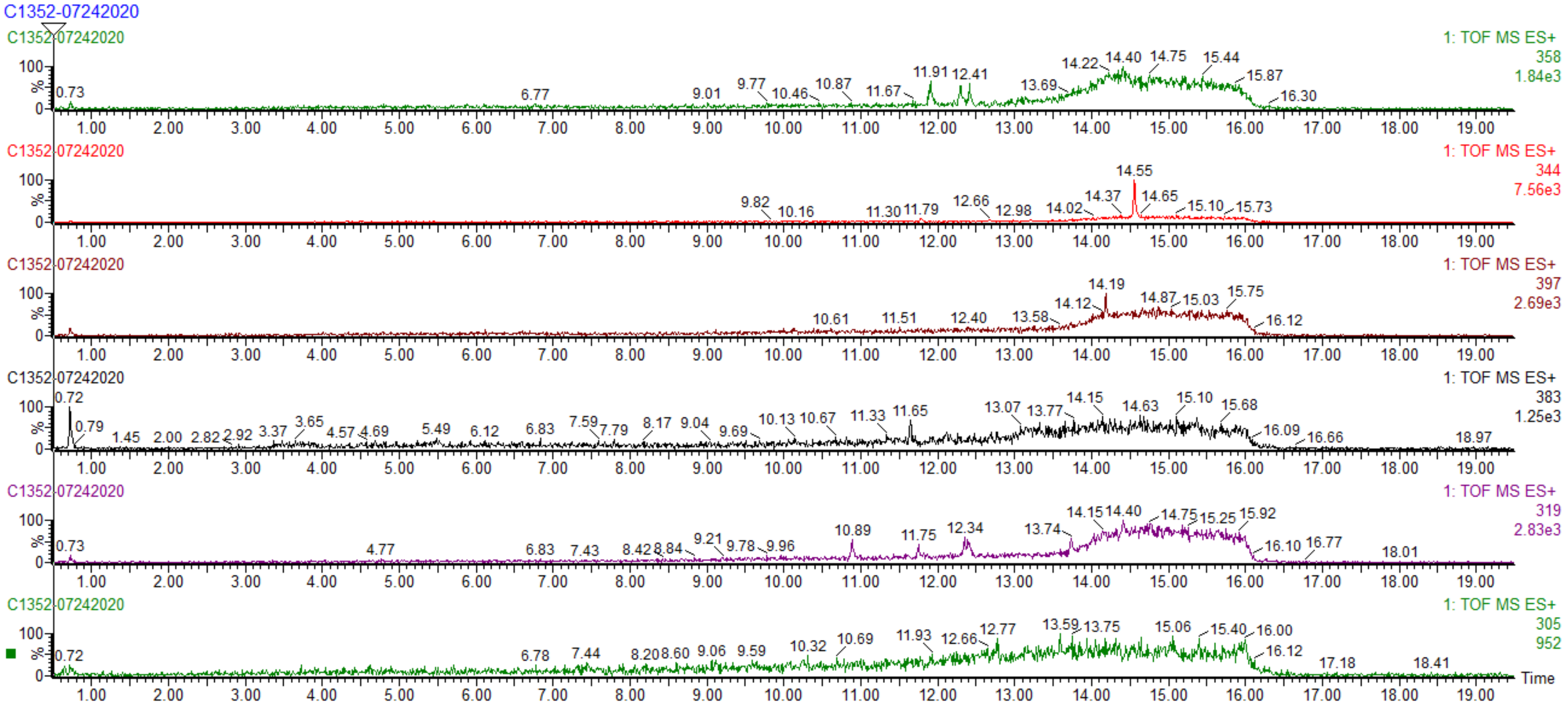

**Figure S55.** Culture broths for each of the six different marine *Bacillus* strains evaluated in this study.

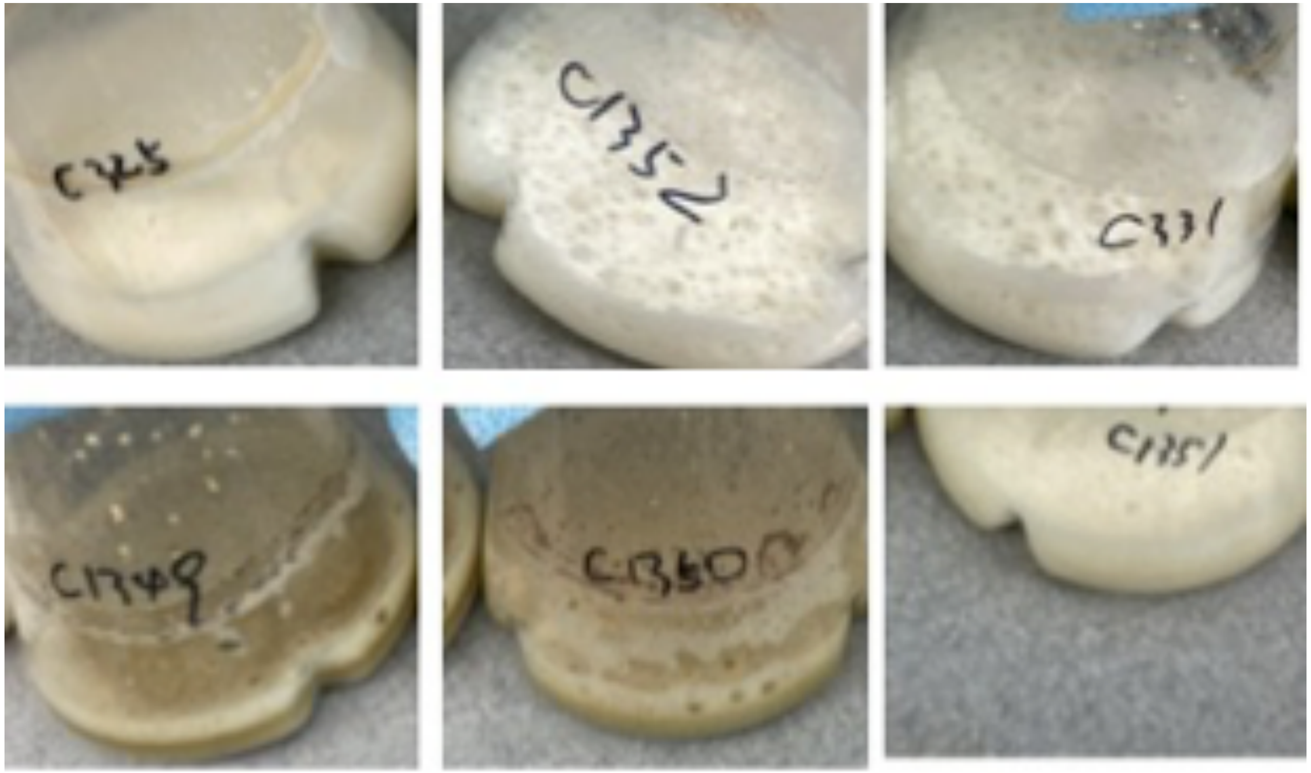

**Figure S56.** Pictures of marine sponge hosts for six different marine *Bacillus* strains evaluated in these studies.

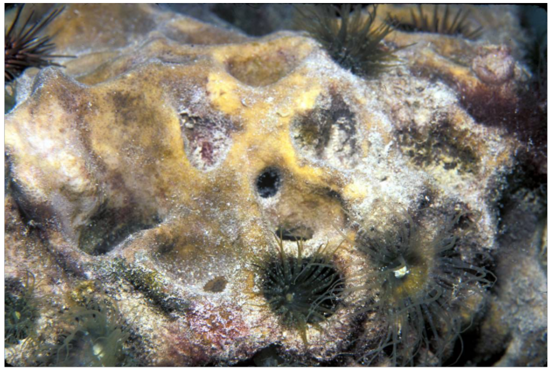

*Geodia gibberosa*

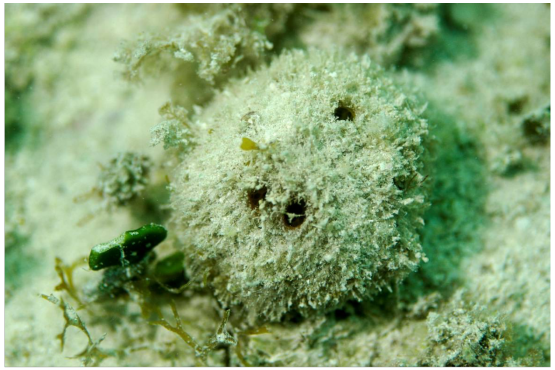

*Cinachyrella apion*

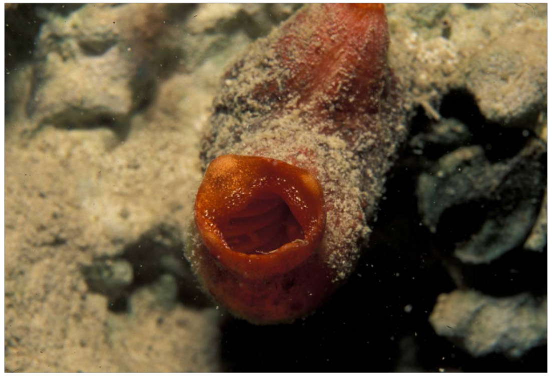

*Microcosmus exasperatus*

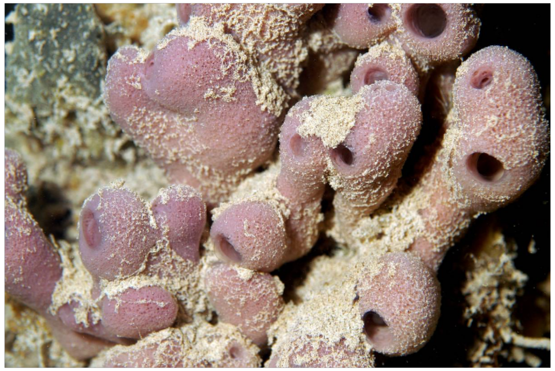

*Chalinula molitba*

## References


1. Quinlan, A. R.; Hall, I. M. *Bioinformatics*. **2010**, *26*, 841−842.

2. Koren, S.; Walenz, B. P.; Berlin, K.; Miller, J. R.; Bergman, N. H.; Phillippy, A. M. *Genome. Res*. **2017**, *27*, 722−736.

3. Blin, K.; Shaw, S.; Steinke, K.; Villebro, R.; Ziemert, N.; Lee, S. Y.; Medema, M. H.; Weber, T. *Nucleic. Acids. Res.* **2019**, *47*, 81−87.
